# Acid Strength Effects on Dimerization during Metal-Free Catalytic Dioxygen Reduction

**DOI:** 10.1021/jacs.4c05708

**Published:** 2024-08-29

**Authors:** Emma N. Cook, Luke A. Flaxman, Amelia G. Reid, Diane A. Dickie, Charles W. Machan

**Affiliations:** Department of Chemistry, University of Virginia, PO Box 400319, Charlottesville, Virginia 22904-4319, United States

## Abstract

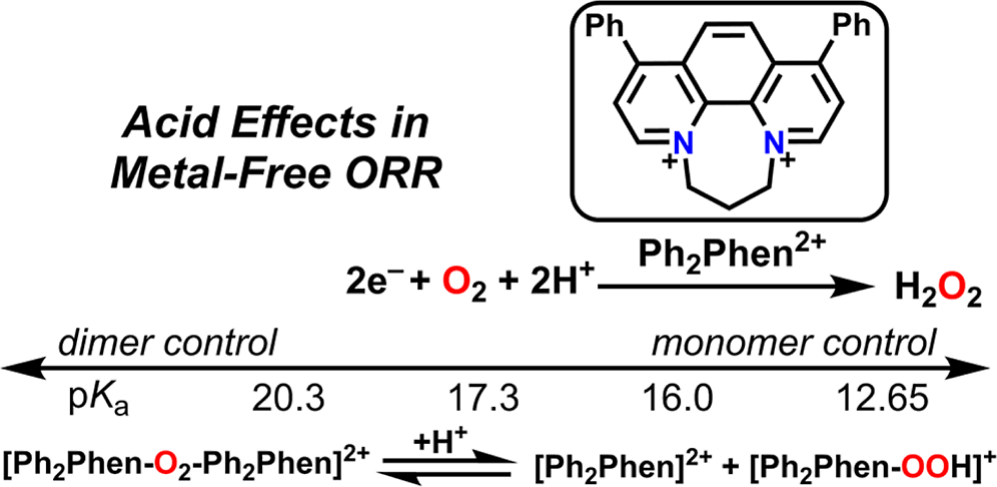

Development of earth-abundant catalysts for the reduction of dioxygen (ORR) is essential for the development of alternative industrial processes and energy sources. Here, we report a transition metal-free dicationic organocatalyst (**Ph**_**2**_**Phen**^**2+**^) for the ORR. The ORR performance of this compound was studied in acetonitrile solution under both electrochemical conditions and spectrochemical conditions, using halogenated acetic acid derivatives spanning a p*K*_a_ range of 12.65 to 20.3. Interestingly, it was found that under electrochemical conditions, a kinetically relevant peroxo dimer species forms with all acids. However, under spectrochemical conditions, strong acids diminish the kinetic contribution of this dimer to the observed rate due to lower catalyst concentrations, whereas weaker acids were still rate-limited by the dimer equilibrium. Together, these results provide insight into the mechanisms of ORR by organic-based, metal-free catalysts, suggesting that balancing redox activity and unsaturated character of these molecules with respect to the p*K*_a_ of intermediates can enable reaction tuning analogous to transition metal-based systems.

## Introduction

The oxygen reduction reaction (ORR) is central to the development of new alternative energy devices as well as a possible route to more environmentally friendly chemical oxidant production. Currently, the best catalysts for the ORR are platinum-based materials, however, the high cost and low earth abundance of Pt preclude it from being a sustainable option.^1^ Inspiration from nature, in combination with the perceived desirability of intrinsic redox flexibility and favorable open-shell ground state configurations, has led to significant focus on first–row transition metals as the basis for developing new electrocatalysts for the ORR.^2−7^ Substantial work has been done to better understand reactivity of dioxygen (O_2_) at molecular transition metal centers, including assessing the structure–function parameters which control selectivity and activity.^2−4,8−12^

Organic molecules are generally unstable in the presence of reactive oxygen species, however studies to improve their stability and reactivity have been of interest in a number of energy-relevant areas including the development of redox-air batteries and effective ORR catalysts.^13−16^ There have been a few reports on homogeneous ORR mediated by organic molecules, including methyl viologen^17^ and substituted methylacridinium salts.^18^ Mechanistic studies showed that both of these catalysts operate via an outer-sphere mechanism to selectively produce hydrogen peroxide (H_2_O_2_). More recently, ORR catalysis reliant on inner-sphere mechanisms has been reported for organic molecules. Gabbaï and co-workers reported a dicarbenium system where a bridging peroxide species was a crucial intermediate during catalysis.^19^ Protonation of this catalyst-bound intermediate resulted in the release of H_2_O_2_. Subsequently, Kiatisevi and co-workers studied an imidazole/benzimidazole-based system for the reduction of O_2_ to either H_2_O_2_ or water (H_2_O) based on the electron-donating or -withdrawing ability of the substituents.^20^ It is worth noting that there has also been significant work on heterogeneous carbon-based catalysts for the ORR,^2,21−24^ as well as a few examples of metal-free porphyrin and subporphyrin compounds that reduce O_2_.^25−29^

Recently, we reported an iminium-based organoelectrocatalyst (im^+^) whose accessible mechanistic pathway (and therefore reaction selectivity) was controlled by the electron source.^30^ Under electrochemical conditions, the concentration of reduced im^0^ was high enough relative to O_2_^•–^ in the reaction-diffusion layer to proceed via an inner-sphere mechanism to generate H_2_O. However, under spectrochemical conditions with decamethylferrocene (Cp*_2_Fe) as a chemical reductant, catalysis operated via an outer-sphere mechanism to form quantitative amounts of H_2_O_2_.

**Figure 1:**
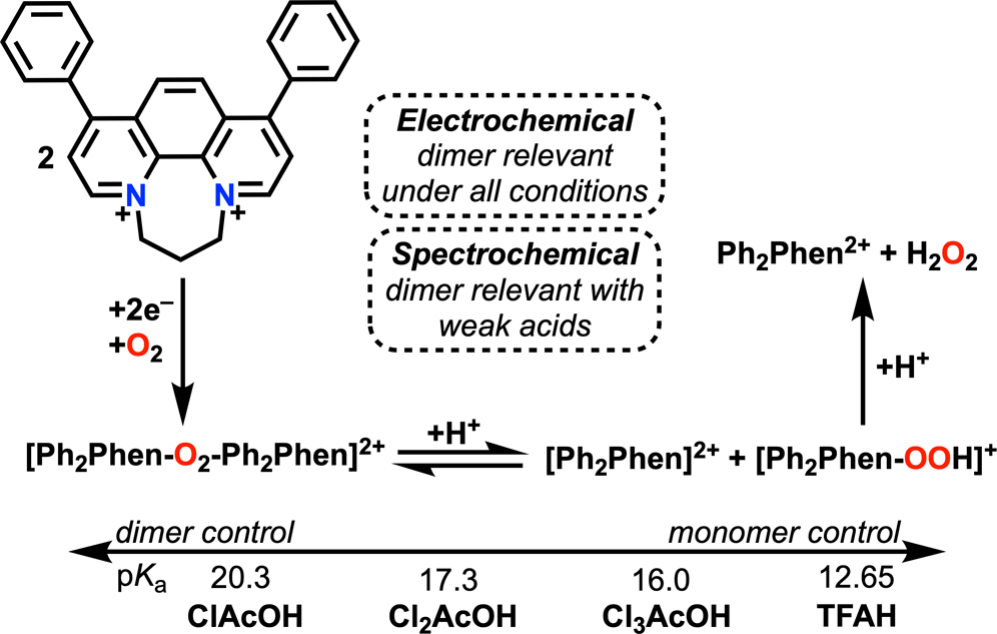
Summary of the work described here on ORR in MeCN, note that a simplified representation of the equilibrium dimerization reaction is depicted.

These previous studies suggested that cationic and unsaturated organic compounds could be viable precatalysts for the ORR. Based on this work, it was reasoned that extended aromaticity provided by a phenanthroline moiety could be used to further stabilize the active catalyst as well as provide framework for facile synthetic tunability in future studies.^31^ Phenanthroline is also readily alkylated to form a highly conjugated dication, or phenanthrolindiium,^32^ that offers interesting structural and reactivity comparisons to the previously studied methyl viologen and carbenium dications.^17,19^ Here, the synthesis and catalytic activity of a phenanthrolindiium salt (**Ph**_**2**_**Phen**^**2+**^) for the ORR with halogenated acetic acid derivates as proton sources is reported (Figure 1). Under both electrochemical and spectrochemical conditions (decamethylferrocene, Cp*_2_Fe as a chemical reductant) we find that the precatalyst **Ph**_**2**_**Phen**^**2+**^ can mediate O_2_ reduction via an initial one-electron outer-sphere mechanism to superoxide (O_2_^•–^), resulting in the formation of a radical–ion pair.^17,25,29^ Based on mechanistic and computational studies, it is proposed that a covalent dimer species containing a bridging peroxo unit between two equivalents of **Ph**_**2**_**Phen**^**2+**^ forms under all conditions. The kinetic contribution of the dimer to the catalytic rate expression under electrochemical and spectrochemical conditions with respect to acid strength suggests that protonation-induced dimer cleavage to a hydroperoxo species is under equilibrium control. Overall, these studies suggest that when factors of redox activity and unsaturated character are adjusted relative to the p*K*_a_ of superoxo and peroxo intermediates, organoelectrocatalytic activity can be tuned in a manner analogous to transition metal-based catalysts.^33^

## Results

### Synthesis and Characterization

**Figure 2:**
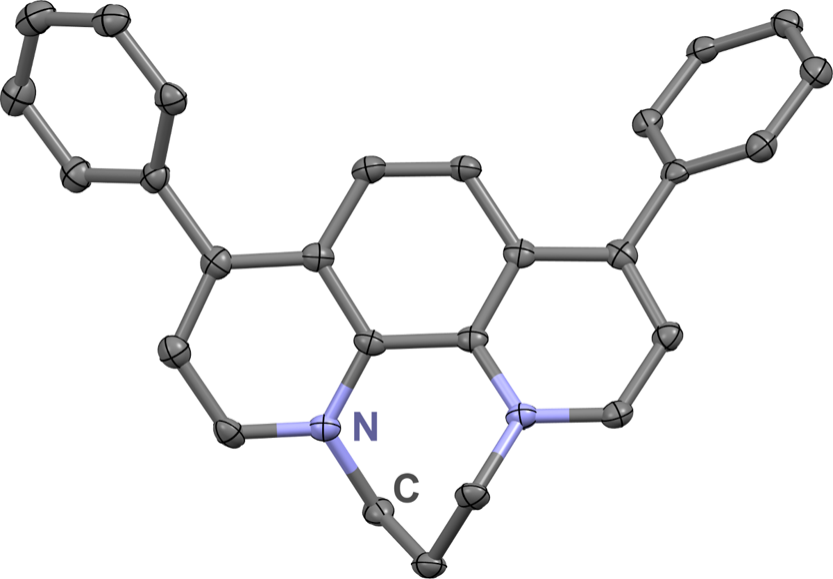
Molecular structure of **Ph**_**2**_**Phen**^**2+**^ obtained from single-crystal X-ray diffraction studies. H atoms and occluded Br counteranions have been removed for clarity. Gray = C, blue = N; thermal ellipsoids at 50%. CCDC 2346924.

The synthesis of 1,11-diphenyl-6,7-dihydro-5*H*-[1,4]diazepino[1,2,3,4-Imn][1,10]phenanthroline-4,8-diium dibromide (**Ph**_**2**_**Phen**^**2+**^; outer-sphere anions neglected from nomenclature) was achieved via a previously reported procedure.^34^ A solution of 4,7-diphenyl-1,10-phenanthroline was allowed to reflux in toluene with a stoichiometric amount of 1,3-dibromopropane until a precipitate formed. Recrystallization of the crude material from dichloromethane resulted in a spectroscopically pure bright orange solid of the dibromide salt of the phenanthrolindiium compound. The **Ph**_**2**_**Phen**^**2+**^ salt was characterized with elemental analysis, NMR and UV–vis spectroscopies (see Supporting Information). Single crystals suitable for X-ray diffraction studies were obtained by slow evaporation from MeCN-*d*_3_ (Figure 2).

### Electrochemical Analysis

**Ph**_**2**_**Phen**^**2+**^ was analyzed by cyclic voltammetry (CV) in MeCN with tetrabutylammonium hexafluorophosphate (TBAPF_6_) as supporting electrolyte. Under Ar saturation conditions there are two, one-electron reversible redox features at −0.73 V vs Fc^+^/Fc and −1.16 V vs Fc^+^/Fc (Figure S4). All further analysis focused on the more positive feature, due to its relevance to O_2_ reactivity. Variable scan rate studies under Ar saturation showed that the peak current density at the reversible reduction feature at −0.73 V vs Fc^+^/Fc had a linear dependence with the square root of the scan rate between 0.025 and 3 V/s, indicative of a diffusion-limited redox response (Figure S5); a diffusion coefficient of 1.14 × 10^–5^ cm^2^·s^–1^ was calculated from the slope.

Upon saturation with O_2_, the first reduction feature becomes irreversible and shifts to more positive potentials (*E*_p_ = −0.71 V vs Fc^+^/Fc), indicating an irreversible chemical reaction occurs following reduction. Evaluating peak potential with respect to scan rate and concentration for irreversible reactions can provide insight into the nature of the electrochemical mechanism.^30,35^ Evolution of peak potential with respect to scan rate revealed a slope of −24.3 mV/decade (Figure S6) while a slope of −12.3 mV/decade (Figure S7) was observed for variable **Ph**_**2**_**Phen**^**2+**^ concentration studies under comparable conditions. These values are intermediate to those expected for EC (reversible electron transfer followed by irreversible chemical reaction) and RSD-type reactions (radical substrate dimerization reactions), either of which could result from the binding of superoxide O_2_^•–^ to **Ph**_**2**_**Phen**^**2+**^ or **Ph**_**2**_**Phen**^**•+**^. Based on these data and experiments discussed in detail below, it is hypothesized that an initial outer-sphere electron transfer occurs from **Ph**_**2**_**Phen**^**•+**^ to produce O_2_^•–^ as part of a radical–ion pair and that a dimeric equilibrium involving [**Ph**_**2**_**Phen**^**2+**^•O_2_^•–^]^+^ and **Ph**_**2**_**Phen**^**•+**^ exists which generates [**Ph**_**2**_**Phen**–O_2_–**Ph**_**2**_**Phen**]^2+^.^30^

**Figure 3:**
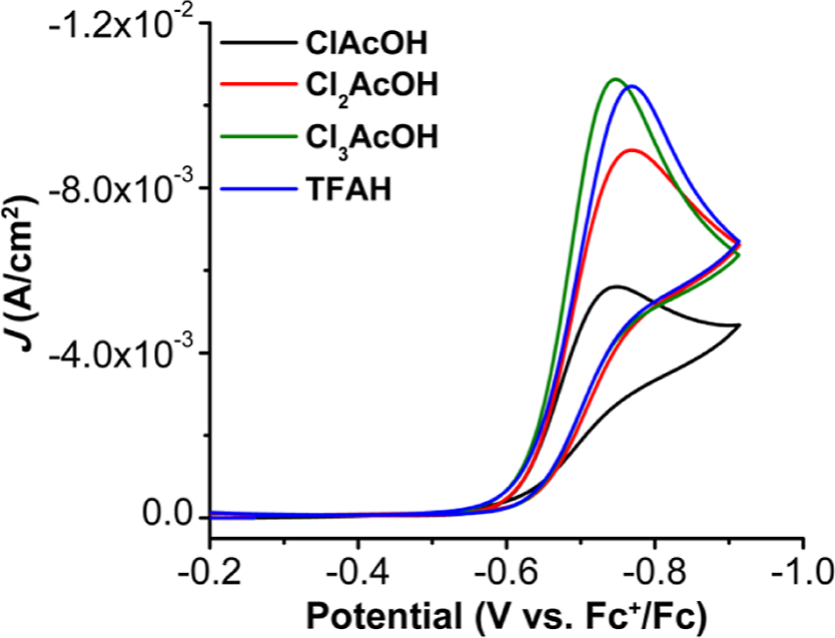
CVs of **Ph**_**2**_**Phen**^**2+**^ under catalytic conditions with ClAcOH (black), Cl_2_AcOH (red), Cl_3_AcOH (green), and TFAH (blue) as proton sources. Conditions: 1 mM **Ph**_**2**_**Phen**^**2+**^, 0.1 M AH, 0.1 M TBAPF_6_/MeCN; O_2_ saturation; glassy carbon working electrode, glassy carbon rod counter electrode, Ag/AgCl pseudoreference electrode; referenced to Fc^+^/Fc internal standard; 100 mV/s scan rate.

Upon addition of TFAH [p*K*_a_ (MeCN) = 12.65]^36^ under Ar saturation conditions, there is minimal change to the initial one-electron redox feature (Figure S9), suggesting that TFAH does not interact with **Ph**_**2**_**Phen**^**2+**^ or its reduced form **Ph**_**2**_**Phen**^**•+**^. However, upon saturation with O_2_ there is an increase in current suggestive of catalytic activity for the ORR (Figure 3, blue). Control rinse tests confirmed that the catalytic current response was due to homogeneous activity of **Ph**_**2**_**Phen**^**2+**^ (Figure S11). It should be noted that cross-tracing can be observed on the return sweep of the catalytic trace at a higher concentration of acid, however, this is overcome at higher scan rates (≥0.8 V/s). Therefore, it is proposed that the cross-tracing can be attributed to the accumulation of an intermediate of the catalytic reaction which is reduced at more positive potentials than the catalytic potential (Figure S10). Rotating-ring disk electrode (RRDE) methods with a glassy carbon disk and roughened gold ring^37^ were used to determine the electrochemical selectivity of ORR by **Ph**_**2**_**Phen**^**2+**^. Under air saturation, this system was found to be 97.3 ± 2.6% selective for H_2_O_2_ with TFAH as the proton source (Figure S16). The estimated overpotential (η_H2O2_) for H_2_O_2_ production is 0.78 V under these conditions (see Supporting Information).^36,38^

To better understand the mechanism of the ORR and its dependence on proton activity, we undertook analogous studies with a variety of acetic acid derivatives: Cl_3_AcOH, Cl_2_AcOH, ClAcOH, which have estimated p*K*_a_ values in MeCN of 16.0, 17.3, and 20.3, respectively (Table S1, see Supporting Information).^39^ In the presence of added proton donor and O_2_, there is a catalytic increase in current for all three (Figure 3). Again, cross-tracing is observed on the return sweep of the catalytic trace that can be overcome at high scan rates (Figures S19, S28, S37). RRDE methods were again used to determine the electrochemical selectivity of the ORR by **Ph**_**2**_**Phen**^**2+**^ with each acid. Under air saturation, Ph_2_Phen^2+^ was found to be 96.9 ± 0.85 and 98.2 ± 7.8% selective for H_2_O_2_ with Cl_3_AcOH and Cl_2_AcOH, respectively. However, under catalytic conditions with ClAcOH as a proton source, reducing current was observed at the Au ring (Figure S43), suggestive of the reduction of an intermediate produced at the disk, precluding selectivity assessment. Control studies with added H_2_O_2_ under aprotic electrochemical conditions revealed that H_2_O_2_ does not interact substantially with reduced **Ph**_**2**_**Phen**^**•+**^ on the electrochemical time scale (Figure S8). However, in the presence of acid (Figures S15, S24, S33, and S42) there is a modest increase in current at the **Ph**_**2**_**Phen**^**2+/•+**^ reduction event and the feature becomes irreversible, suggesting that there is minor activity for H_2_O_2_ reduction. Upon saturating the protic solution with O_2_ ORR catalytic current is recovered in all cases, which is consistent with the proposal of a catalytic reaction where H_2_O_2_ is not a discrete intermediate en route to further reduction under these conditions.

Variable concentration studies were performed in order to develop a better mechanistic picture of the ORR.^40^ Interestingly, for each added acid there is an observed half-order dependence on catalyst concentration (Figures S12, S21, S30, & S39). A first-order dependence on acid concentration was observed for TFAH (Figure S13), Cl_2_AcOH (Figure S31), and ClAcOH (Figure S40), while Cl_3_AcOH was in between half- and first-order (Figure S22). Finally, mixed-order dependence on O_2_ concentration was observed for all proton sources (Figures S14, S32, & S41), with the exception of Cl_3_AcOH (Figure S23), precluding definitive rate law expressions. Based on the half-order rate dependence on **Ph**_**2**_**Phen**^**2+**^ concentration and the general observance of mixed-order dependence on O_2_ concentration, it is proposed that a bridging dimer species forms in the presence of excess **Ph**_**2**_**Phen**^**•+**^ in the reaction-diffusion layer. Consistent with this interpretation, an assessment of turnover frequency (TOF) using CV methods showed an increase in rates as catalyst concentration was decreased for all acids (Table S2; Figures S44–S47) as well as a dependence of rate on acid strength. At the lowest catalyst concentration (ca. 0.2 mM), the use of TFAH (p*K*_a_ = 12.65) resulted in a TOF of 1.73 × 10^3^ s^–1^, which dropped to 5.78 × 10^2^ s^–1^ for ClAcOH (p*K*_a_ = 20.3).

### Spectrochemical Analysis

**Figure 4:**
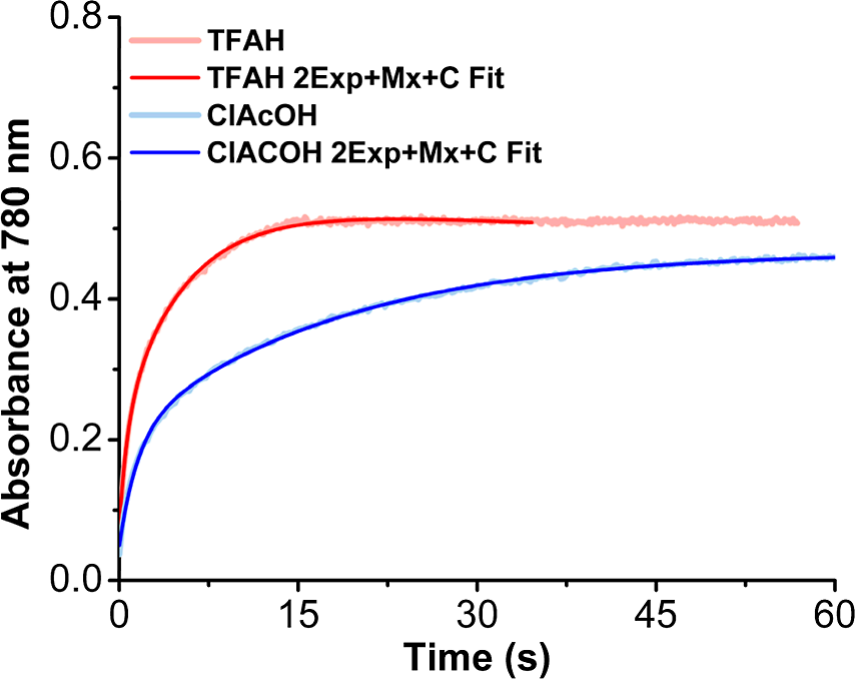
Change in absorbance at 780 nm over time as a result of the formation of [Cp*_2_Fe]^+^ by ORR catalyzed by **Ph**_**2**_**Phen**^**2+**^ with TFAH (red) and ClAcOH (blue). Conditions: **Ph**_**2**_**Phen**^**2+**^ = 40 μM, AH = 50 mM, O_2_ = 4.05 mM, Cp*_2_Fe = 1 mM; control: TFAH = 50 mM, O_2_ = 4.05 mM, Cp*_2_Fe = 1 mM; *R*^2^ = 0.999.

Catalytic ORR activity of **Ph**_**2**_**Phen**^**2+**^ with each acid was also studied by stopped-flow UV–vis methods using Cp*_2_Fe as a chemical reductant (Figure 4). The spectral handle of [Cp*_2_Fe]^+^ at 780 nm was used to monitor the progress of the reaction to extract kinetic parameters. The observed rate law for each system was determined by independently varying the concentration of **Ph**_**2**_**Phen**^**2+**^, acid, O_2_, and reductant (see Supporting Information). With TFAH, there is an observed first-order dependence on **Ph**_**2**_**Phen**^**2+**^, TFAH, and O_2_ concentration (eq 1). With Cl_3_AcOH, the rate law becomes independent of O_2_ concentration, and there is an observed first-order dependence on **Ph**_**2**_**Phen**^**2+**^ and acid concentration (eq 2). Interestingly, with weaker acids, Cl_2_AcOH and ClAcOH, there is a shift in rate law to a half-order dependence on **Ph**_**2**_**Phen**^**2+**^ and first-order dependence on acid (eq 3).
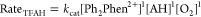
1
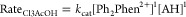
2
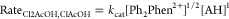
3

A Ti(O)SO_4_ colorimetric assay was used to determine the selectivity for the ORR by **Ph**_**2**_**Phen**^**2+**^ with each acid, as previously reported.^8,12,41−43^ It was found that the selectivity of **Ph**_**2**_**Phen**^**2+**^ for H_2_O_2_ shows an apparent inverse dependence on acid activity, going from 93.2 ± 1.4% selectivity with TFAH to 24.0 ± 6.2% selectivity for H_2_O_2_ with ClAcOH (Table 1, Figures S72–S76) after all Cp*_2_Fe is consumed. H_2_O_2_ selectivity was evaluated over the course of the ORR with ClAcOH to determine if hydrogen peroxide reduction (H_2_O_2_RR) was a competing process (Figure S76, Table S4). After 30 s there was an observed 67.4 ± 5.9% selectivity for H_2_O_2_, however, after 5 min H_2_O_2_ selectivity diminished to 22.0 ± 5.2%. This suggests that H_2_O_2_ is being produced during catalysis before being further reduced to H_2_O.

**Table 1:** Summary of Spectrochemical Activity and Selectivity of ORR by **Ph**_**2**_**Phen**^**2+**^ with Each Proton Donor

acid (p*K*_a_ (MeCN))	electrochemical *k*_obs_ (×10^2^ s^–1^)^a^	electrochemical % H_2_O_2_ selectivity (*n*_cat_)	spectrochemical *k*_obs_ (×10^2^ s^–1^)	spectrochemical % H_2_O_2_ selectivity (*n*_cat_)
TFAH (12.65)	10.1	97.3 ± 2.6 (2.11)	173	93.2 ± 1.4 (2.14)
Cl_3_AcOH (16.0)	6.98	96.9 ± 0.85 (2.12)	115	84.8 ± 5.8 (2.30)
Cl_2_AcOH (17.3)	6.34	98.2 ± 7.8 (2.07)	1.06	82.3 ± 3.2 (2.35)
ClAcOH (20.3)	3.40	n/a	0.431	24.0 ± 6.2 (3.52)

a Data obtained at catalyst concentrations of ca. 0.8 mM.

Analysis of H_2_O_2_RR with all acids under spectrochemical conditions showed slight activity (Figures S53, S59, S65, & S71), however, the difference in apparent activity between the apparent rates of the ORR and the H_2_O_2_RR was the smallest in the case of ClAcOH. It is also important to emphasize that all spectrochemical conditions are limited by the amount of reductant by experimental design, so that they can be run to completion without being limited in other substrate. Therefore, it is proposed that the apparent lower selectivity for H_2_O_2_ with the ClAcOH proton donor is the result of competitive H_2_O_2_ reduction while reductant is available; for stronger acids the chemical reductant is almost completely consumed by the relatively faster ORR process, given the greater difference in rate with H_2_O_2_RR. Consistent with this interpretation, stability tests showed that H_2_O_2_ was stable in the presence of **Ph**_**2**_**Phen**(2)^+^ with each of the acids (Figures S77–S80), with quantitative recovery of added H_2_O_2_ (Table S5) verifying that reductant must be present for this reaction to occur.

### Computational Analysis

**Figure 5:**
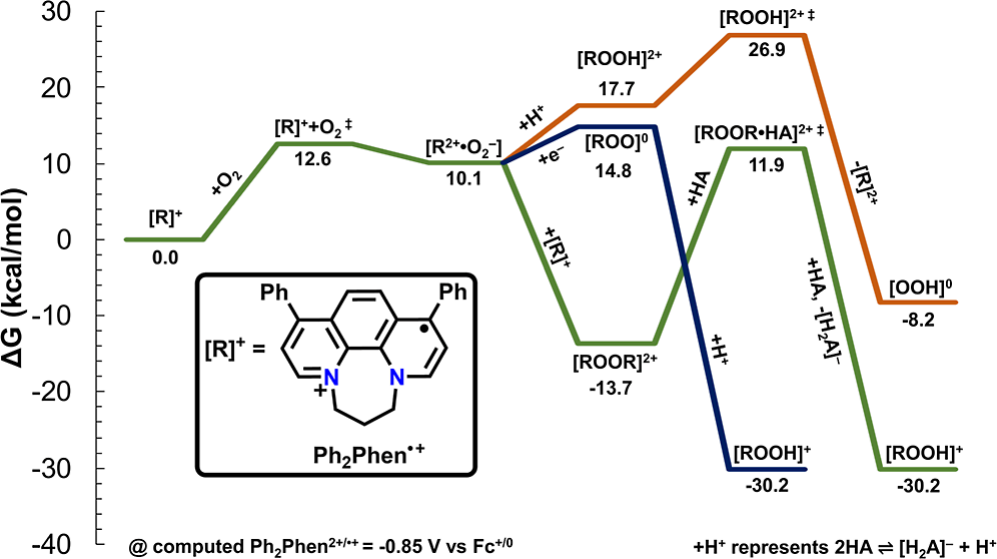
Reaction free energy diagram comparing possible pathways for O_2_ reduction by **Ph**_**2**_**Phen**^**2+**^ obtained from computational methods considering TFAH as the acid and considering homoconjugation during proton transfer.

In order to better understand the relevance of dimerization to the mechanistic data, computational studies were undertaken using the approach previously used for the iminium-based catalyst.^30^ Geometries were optimized and concentration-corrected thermochemical data were obtained using Gaussian 16 at the B3LYP-D3(BJ)/def2-TZVP level of theory before the energies were refined using ORCA 5.0 at the DLPNO–CCSD(T1)/cc-pVTZ level (see Supporting Information).^44−58^ All proton transfer thermochemistry consider the effect of homoconjugation, where proton transfer is accompanied by the favorable equilibrium association of the resultant conjugate base with a second equivalent of the acid (A^–^ + HA ⇌ HA_2_^–^).^59^ A reduction potential of −0.85 V vs Fc^+^/Fc was estimated for **Ph**_**2**_**Phen**^**2+/•+**^, in good agreement with the experimental value of −0.73 V. A transition state for outer-sphere electron transfer from **Ph**_**2**_**Phen**^**•+**^ to generate O_2_^•–^ was found to be uphill by +12.6 kcal/mol (Figure 5) and the corresponding imaginary mode indicated that the 4-position of a pyridyl subunit participated in the reaction. Note that in Figure 5, the **Ph**_**2**_**Phen**^**2+**^ core is abbreviated as **[R]**^**2+**^. A potential energy surface scan revealed a well on the other side of this saddle point, with a distance between the O_2_ subunit and 4-position of the pyridyl moiety which was too long for a formal single bond (1.58 Å) and could not be optimized directly as an adduct. Based on literature precedent,^17,25,29^ it was reasoned that this could attributed to the stabilizing effect of a radical–ion pair between O_2_^•–^ and **Ph**_**2**_**Phen**^**2+**^, abbreviated as **[R**^**2+**^**•O**_**2**_^**–**^**]** in Figure 5. An optimization constrained only at this carbon–oxygen distance produced a representative minimum energy for the radical–ion pair. Within the limits imposed by this constrained approximation, the formation of the radical–ion pair is estimated to be endergonic by +10.1 kcal/mol.

**Figure 6:**
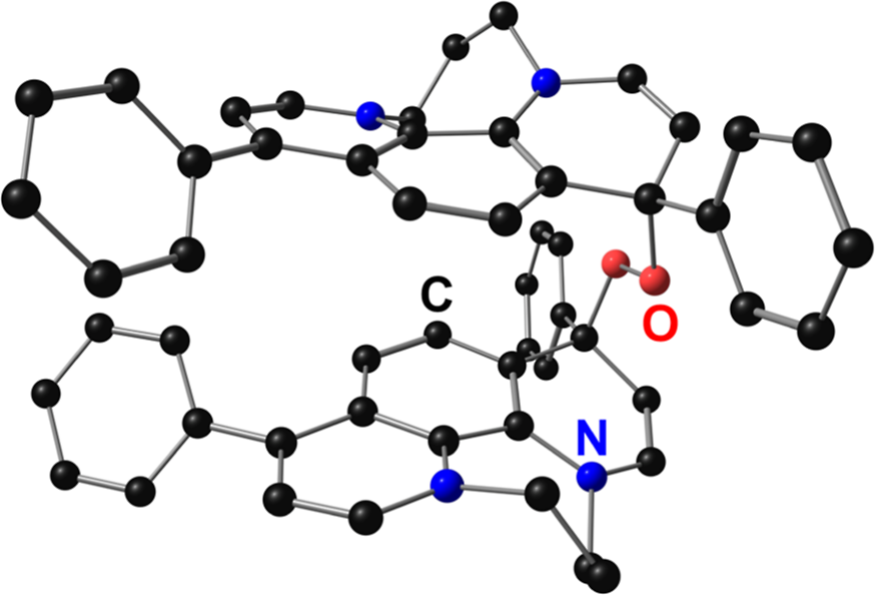
Proposed dimer species implied by mechanistic data. A peroxo subunit bridges two equiv of **Ph**_**2**_**Phen**^**2+**^ at the 4-position of a pyridine subunit. Gray = C, blue = N, red = O.

The radical–ion pair represents the primary reaction pathway branching point to generate H_2_O_2_. A comparison of reactions where **[R**^**2+**^**•O**_**2**_^**–**^**]** undergoes protonation, reduction, or dimerization involving **Ph**_**2**_**Phen**^**•+**^ suggests that the last of these is the most likely pathway (Figure 5, green). Although no barrier was identified, the dimerization of **[R**^**2+**^**•O**_**2**_^**–**^**]** with **[R]**^**+**^ to generate **[ROOR]**^**2+**^ (Figure 6) is downhill by −23.8 kcal/mol (Figure 5, green). In this structure, a pyridyl ring on each equivalent of **[R]**^**2+**^ is dearomatized by the insertion of a peroxo subunit at the 4-position. Reduction of **[R**^**2+**^**•O**_**2**_^**–**^**]** was found to be uphill by +4.7 kcal/mol, while protonation was +7.6 kcal/mol uphill (Figure 5, blue and orange). Although a concerted proton–electron transfer reaction to convert **[R**^**2+**^**•O**_**2**_^**–**^**]** to **[ROOH]**^**+**^ is very favorable, the kinetic relevance of the dimer under reaction conditions suggests that only asynchronous proton and electron transfer reactions are possible. From these results it can be concluded that dimerization is rapid and favorable, particularly under electrochemical conditions where an excess of **Ph**_**2**_**Phen**^**•+**^ is present.

Although the transition state corresponding to **[ROOR]**^**2+**^ dimer cleavage for the strongest acid (TFAH; +25.6 kcal/mol) was only slightly lower than the weakest acid (ClAcOH; +26.3 kcal/mol), ΔΔ*G*^‡^ = 0.7 kcal/mol, the reaction thermodynamics greatly favored cleavage by TFAH, ΔΔ*G* = 7.5 kcal/mol. Analysis of the TS structure **[ROOR•HA]**^**2+‡**^ for both acids showed slight elongation of the carboxylic OH bonds for the acids: TFAH 0.977 to 1.052 Å and ClAcOH 0.975 to 1.012 Å. By comparison, carbon–oxygen bond distances connecting one of the pyridyl subunits to the peroxo bridge at the 4-position were greatly elongated from 1.466 Å in the symmetric **[ROOR]**^**2+**^ (Figure 6) to 1.950 Å for TFAH and 1.854 Å for ClAcOH. Based on these bonding metrics, proton transfer in transition state appears to be very early in the overall reaction coordinate, suggesting that the dimer cleavage is mediated primarily by the hydrogen bonding effect of the acid. This would explain the relatively minor difference in the reaction barriers between acids with significantly different proton activities (Δp*K*_a_ = 7.7) and the high degree of equilibrium control observed on the rate of catalysis. The **[ROOR]**^**2+/+**^ reduction potential was estimated to be −1.74 V vs Fc^+^/Fc, too negative to be relevant to dimer cleavage under reaction conditions.

The formation of **[ROOH]**^**+**^ from **[ROOR]**^**2+**^ via **[ROOR•HA]**^**2+‡**^ with TFAH as the proton source and **[R]**^**2+**^ as the co-product is exergonic by −16.4 kcal/mol (Figure 5). Protonation of the hydroperoxo **[ROOH]**^**+**^ to cleave the carbon–oxygen bond, rearomatize the catalyst to its precursor phenanthrolindiium state **[R]**^**2+**^, and release H_2_O_2_ was found to have a reaction barrier of +17.1 kcal/mol **[ROOH•HA]**^**+‡**^ and to be downhill overall by −19.5 kcal/mol with TFAH as the acid (Figure S81). The comparable barrier with the weaker acid ClAcOH was +20.4 kcal/mol and the reaction downhill by −12.0 kcal/mol (Figure S82). The kinetic relevance of this step is presumably observed experimentally as the difference in the observed rates with the different acids, despite the lower predicted barrier than dimer cleavage, which is consistent with regarding the dimerization as under equilibrium control. Although overall these data qualitatively agree with the conclusions drawn from the mechanistic experiments, the barrier for the rate-determining step (dimer cleavage, +25.6 kcal/mol with TFAH), is estimated to be higher than might be expected based on the experimentally determined TOFs (Table 1). Since dimer cleavage appears to rely heavily on hydrogen-bonding interactions, vide supra, it is possible that multiple equivalents of acid could be involved during the reaction. Further, considering the favorability of homoconjugation during proton transfer for these carboxylic acids (A^–^ + HA ⇌ HA_2_^–^), it can also be speculated that the participation of multiple acid equivalents could shift the reaction coordinate further from hydrogen bonding to proton transfer, which may also have a beneficial effect and modeling such possibilities warrants future study.

## Discussion

**Scheme 1:**
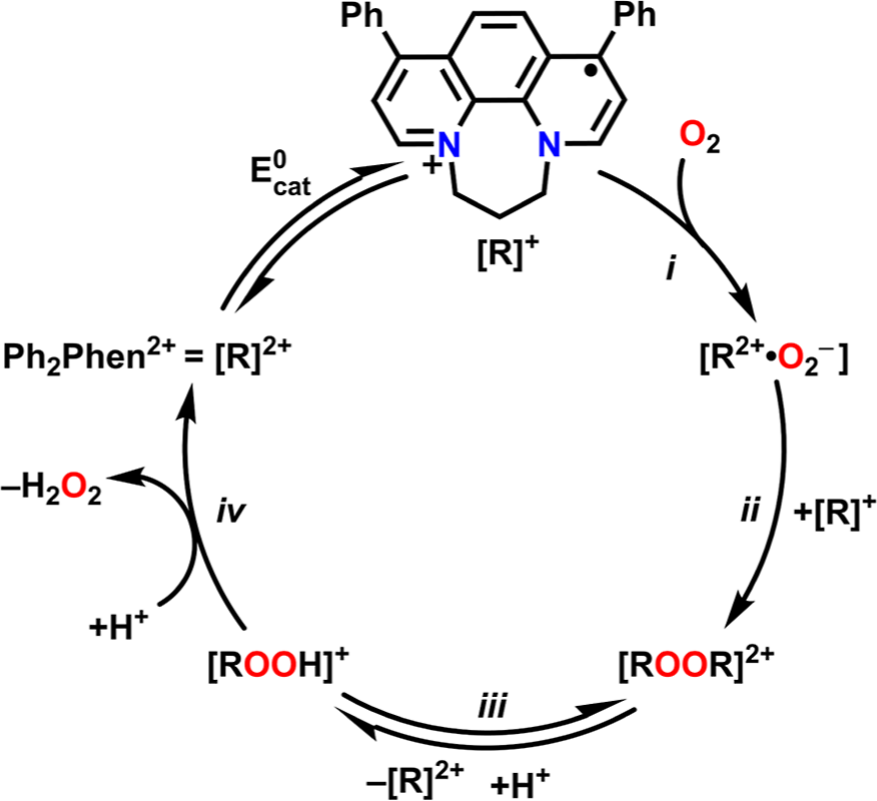
Proposed Mechanism for the ORR Catalyzed by **Ph**_**2**_**Phen**^**2+**^

Based on both electrochemical and spectrochemical results, we are able to propose a catalytic cycle for the ORR by **Ph**_**2**_**Phen**^**2+**^ with TFAH, Cl_3_AcOH, Cl_2_AcOH, and ClAcOH (Scheme 1). Starting from **[R]**^**2+**^, a single-electron reduction results in the formation of **[R]**^**+**^, which transfers an electron to O_2_ via an outer-sphere mechanism to form superoxide (O_2_^•–^) (i) before forming a radical–ion pair **[R**^**2+**^**•O**_**2**_^**–**^**]**. In addition to aligning with the mechanistic and computational data described above, the proposals of outer-sphere electron transfer and the stabilizing effect of a radical–ion pair are also consistent with reports on similar systems.^17,18,30,60^ Subsequently, the radical–ion pair **[R**^**2+**^**•O**_**2**_^**–**^**]** can dimerize (ii) with another equivalent of the reduced precatalyst **[R]**^**+**^ to generate a bridging peroxo dimer species **[ROOR]**^**2+**^. In the presence of acid, this dimer species exists in equilibrium (iii) with a hydroperoxo species **[ROOH]**^**+**^ and the precatalyst **[R]**^**2+**^. Acid strength alters the barrier of this equilibrium reaction involving dimer cleavage very little (estimated ΔΔ*G*^‡^ = 0.7 kcal/mol for Δp*K*_a_ = 7.7), but reaction thermodynamics change significantly over the same range (ΔΔ*G* = 7.5 kcal/mol). The subsequent protonation step to generate H_2_O_2_***iv*** shows a greater sensitivity to acid p*K*_a_ in its reaction barrier and comparable differences in reaction thermodynamics. Since this step is irreversible and proton donor activity controls the position of equilibrium (iii), a kinetic rate enhancement is observed based on acid p*K*_a_.

It has previously been established in MeCN that the second-order rate constant for disproportionation of HO_2_^•^ is on the order of 10^6^–10^7^ M^–1^ s^–1^.^60−63^ A reasonable reaction pathway was identified for the disproportionation mechanism via computational methods (Figure S81), as were monomeric pathways **[ROOH]**^**+**^ involving stepwise (Figure 5, blue) or concerted proton and electron transfer. However, kinetic analysis is consistent with a bridging peroxo dimer species sensitive to the available concentration of **[R]**^**2+**^ and **[R]**^**+**^. Under electrochemical conditions, there is an excess of reduced **[R]**^**+**^ in the reaction-diffusion layer, which pushes the equilibrium toward dimer formation, Scheme 1(i). Further, the concentration of the precatalyst **[R]**^**2+**^ under electrochemical conditions is at least an order of magnitude greater than under spectrochemical conditions, which would favor the reactants in acid-sensitive dimer cleavage equilibrium (iii). This is supported by the observed half-order dependence on catalyst concentration under electrochemical conditions for all acids as well as the mixed-order dependence on O_2_ concentration.^64,65^

Under spectrochemical conditions only the two weakest acids, Cl_2_AcOH and ClAcOH, show a half-order dependence of activity on catalyst concentration. Unlike the reaction-diffusion layer of the electrode under electrochemical conditions, lower loading of **[R]**^**2+**^ would favor the forward direction of the acid-mediated dimer cleavage equilibrium (iii). Indeed, the presented data show that the kinetic relevance of the peroxo dimer species is dependent on acid strength under spectrochemical conditions: sufficiently strong acids will drive the reaction pathway toward the irreversible formation of H_2_O_2_. Interestingly, this proposed mechanism shares similarities with that for the dicarbenium catalyst reported by Gabbaï and co-workers, which invoked a comparable bridging peroxo species.^19^

Consistent with the proposal of an inner-sphere interaction, ^1^H NMR studies of **Ph**_**2**_**Phen**^**2+**^ in the presence of Cp*_2_Fe and O_2_ demonstrated the appearance of a new species along with a small amount of the precursor **Ph**_**2**_**Phen**^**2+**^ (Figure S83). Further, when a solution of **Ph**_**2**_**Phen**^**2+**^ and Cp*_2_Fe is exposed to O_2_ and a slight excess TFAH, full conversion back to **Ph**_**2**_**Phen**^**2+**^ is observed (Figure S84). When a slight excess of ClAcOH is instead used as a proton source, some **Ph**_**2**_**Phen**^**2+**^ is recovered but there remains a mixture of products (Figure S85). Although we were unable to characterize the intermediate produced with reductant and O_2_ under aprotic conditions further (Figure S83), it is worth noting that this species formed with a 1:1 ratio of reductant to **Ph**_**2**_**Phen**^**2+**^ and that there is minimal **Ph**_**2**_**Phen**^**2+**^ present. Based on our computational and mechanistic work, the species could be either **[R**^**2+**^**•O**_**2**_^**–**^**]**, **[ROO]**^**0**^, or **[ROOR]**^**2+**^ (Figure 5). Only **[R**^**2+**^**•O**_**2**_^**–**^**]** and **[ROOR]**^**2+**^ have the required ratio of reductant to **Ph**_**2**_**Phen**^**2+**^ and the latter seems most likely from indirect experimental evidence. Based on literature precedent, experimental evidence of a similar radical–ion pair has only been directly obtained through low temperature quenching experiments.^17,25,29^ Considering this tentative assignment of dimerization under these conditions, the incomplete conversion of this intermediate species with ClAcOH is consistent with the observation that the weaker acid cannot completely convert back to **[Ph**_**2**_**Phen]**^**2+**^ at low acid concentrations (Figure S85).

These observations under stoichiometric conditions are generally consistent with the observed half-order concentration dependence on catalyst when weaker acids are used as the proton donor under spectrochemical conditions. Under electrochemical conditions, a half-order concentration dependence observed for all acids because of the greater concentration of the precatalyst **Ph**_**2**_**Phen**^**2+**^ and the cationic radical species **Ph**_**2**_**Phen**^**•+**^ in the reaction-diffusion layer. As described above, TOF values obtained by CV methods showed increased values for all acids as the concentration of **Ph**_**2**_**Phen**^**2+**^ was lowered. It is also worth noting that proton donors with greater activity than ClAcOH are required to quantify H_2_O_2_ production by RRDE methods, since these have sufficient driving force to exit the acid-mediated dimer cleavage equilibrium under these conditions.

As described above, the low observed selectivity for H_2_O_2_ when ClAcOH is used as a proton donor can be explained by the relatively enhanced role of H_2_O_2_RR during the progress of ORR, which can consume some of the H_2_O_2_ produced by ORR. By comparison, for stronger acids, ORR is significantly more rapid than H_2_O_2_RR, minimizing its impact on the observed % H_2_O_2_ selectivity. ^1^H NMR studies of **Ph**_**2**_**Phen**^**•+**^ with added urea•H_2_O_2_ suggest an inner-sphere interaction with H_2_O_2_ in the presence of a chemical reductant. Again, **Ph**_**2**_**Phen**^**2+**^ is reformed with a sufficiently strong acid, as was observed under analogous conditions with O_2_ (Figure S86). Importantly, the loss of molecular symmetry in the NMR data imply that it is again the 4-position of a pyridyl subunit that participates in an inner-sphere reaction. The presence of an inner-sphere reaction between H_2_O_2_ and **Ph**_**2**_**Phen**^**•+**^ suggests that there is a possibility of accessing selectivity for H_2_O in future studies, with proper synthetic modification of the phenyl group appended here.

Overall, the rate of ORR with **Ph**_**2**_**Phen**^**2+**^ is comparable to that of our previously reported iminium-based systems, as well as more rapid than the previously reported dications (Table S7). The mechanistic pathway presented here implies that future studies exploring the substituents on the phenanthrolindiium core can be used to tune reaction activity and selectivity similar to transition metal-based catalyst systems. Provocative conceptual parallels can be drawn with the mechanisms of Cu-based homogeneous catalysts for the ORR, which are generally proposed to undergo only single-electron transfers and dimerize in a manner analogous to the purely organic system described here.^33^ The limits of this analogy are currently being explored in ongoing experiments.

## Conclusions

Here, a new organic-based catalyst for the ORR under both electrochemical and spectrochemical conditions that reaches, and in some cases surpasses, reported activity of other cation-based metal-free electrocatalysts for the ORR^17,18,30^ is described. We found that the activity and mechanism of the ORR by **Ph**_**2**_**Phen**^**2+**^ can be tuned by acid strength. Using acetic acid derivates with p*K*_a_ values ranging from 12.65 to 20.3, it was found that a bridging peroxo dimer species formed in the presence of O_2_ and reduced catalyst, **Ph**_**2**_**Phen**^**•+**^. Further, acid strength can tune the kinetic relevance of this dimer, shifting the distribution of the equilibrium involving its acid-mediated cleavage. Excitingly, these data imply a comparable tunability of the ORR activity of metal-free electrocatalysts to that known for transition metal-based systems. The implications of these mechanistic observations are currently being explored under additional reaction conditions and through the modification of the phenanthrolindiium framework.

## Supporting Information Available

Description of experimental details and methods, electrochemical data, and stopped-flow data (PDF)
Computational coordinates (XYZ)

## Supplementary Material

ja4c05708_si_001.pdf

ja4c05708_si_002.xyz
