# Supplementary material for: Acid Strength Effects on Dimerization during Metal-Free Catalytic Dioxygen Reduction
Source: J Am Chem Soc. 2024 Aug 29;146(36):24892–900. doi: 10.1021/jacs.4c05708 (PMC11403605; doi:10.1021/jacs.4c05708)
Supplement: Supplementary file 1 — ja4c05708_si_001.pdf [file ja4c05708_si_001.pdf]

SUPPLEMENTARY INFORMATION for

Acid Strength Effects on Dimerization During Metal-Free Catalytic Dioxygen Reduction

Emma N. Cook, Luke A. Flaxman, Amelia G. Reid, Diane A. Dickie, and Charles W. Machan*

*machan@virginia.edu; ORCID 0000-0002-5182-1138

E.N.C. ORCID 0000-0002-0568-3600
L.A.F. ORCID 0009-0002-5315-4727
A.G.R. ORCID 0000-0002-2868-4091
D.A.D. ORCID 0000-0003-0939-3309

Department of Chemistry, University of Virginia,
PO Box 400319, Charlottesville, VA 22904-4319, United States of America

Table of Contents

Experimental Methods 8

Synthesis and Characterization 8

Synthesis of 1,11-diphenyl-6,7-dihydro-5H-[1,4]diazepino[1,2,3,4-lmn][1,10]phenanthroline-4,8-diium bromide (**Ph_2Phen^{2+}**). 8

Figure S1. (A) UV-vis serial dilution absorbance data from **Ph_2Phen^{2+}** in MeCN 8

Figure S2. ^{1}H-NMR spectrum of **Ph_2Phen^{2+}** in MeOD-d_4; Varian 600 MHz. 9

Figure S3. ^{13}C{^{1}H} NMR of **Ph_2Phen^{2+}** in MeOD-d_4; Varian 150 MHz. 10

Synthesis of 1,11-diphenyl-6,7-dihydro-5H-[1,4]diazepino[1,2,3,4-lmn][1,10]phenanthroline-4,8-diium hexafluorophosphate (**$Ph_2Phen(PF_6)_2$**). 11

Estimation of pK_a Values for Cl_3AcOH, Cl_2AcOH, and ClAcOH in MeCN 11

Table S1. Summary of calculated pK_a values in MeCN. 11

Electrochemical Analysis of Ph_2Phen^{2+} 11

Electrochemistry 11

Rotating Ring-Disk Electrode Methods 11

Description of Au Ring Roughening Procedure. 11

Description of RRDE Collection Efficiency. 12

RRDE Experiments. 12

Figure S4. CV of **Ph_2Phen^{2+}** under Ar. 13

Figure S5. (A) CVs of **Ph_2Phen^{2+}** under Ar saturation at various scan rates. (B) Square root of scan rate versus current density in (A). 13

Figure S6. (A) CVs of **Ph_2Phen^{2+}** under O_2 saturation at various scan rates. (B) Logarithm of the scan rate versus the reduction peak potential in (A). 14

Figure S7. (A) CVs of **Ph_2Phen^{2+}** under O_2 saturation at varying concentrations. (B) Logarithm of **Ph_2Phen^{2+}** concentration versus the reduction peak potential in (A). 14

Figure S8. CVs of Ph_2Phen^{2+} with and without added 6.7 mM urea•H_2O_2 under Ar and O_2 saturation. 15

Electrochemical Analysis with TFAH 15

Figure S9. (A) CVs of **Ph_2Phen^{2+}** under Ar (black), O_2 (red) and with 0.1 M TFAH under Ar saturation (green). (B) CVs from A and catalytic trace shown (blue) with Ph_2Phen^{2+} and 0.1 M TFAH under O_2 saturation. 15

Figure S10. (A) CVs of **Ph_2Phen^{2+}** under catalytic conditions with 0.13 M TFAH at 0.1 V/s (black) and 0.8 V/s (red) 16

Figure S11. Rinse test of **Ph_2Phen^{2+}** and TFAH. Ph2Phen2+ under catalytic conditions (black trace), rinse test (red trace), and CV of TFAH under O_2 saturation (green trace). 17

Figure S13. (A) CVs of **Ph_2Phen^{2+}** under catalytic conditions with variable TFAH concentrations. (B) Logarithm of TFAH concentration versus the logarithm of current density from (A). 18

Figure S14. (A) CVs of **Ph_2Phen^{2+}** under catalytic conditions with variable O_2 concentrations. (B) Logarithm of O_2 concentration versus the logarithm of current density from (A)............18

Figure S15. (A) CVs of **Ph_2Phen^{2+}** in the presence of 6.7 mM urea•H_2O_2 under Ar and O_2 saturation with added 0.11 M TFAH. (B) CVs of **Ph_2Phen^{2+}** in the presence of 6.7 mM urea•H_2O_2 with added 0.11 M TFAH under O_2 saturation. 19

Figure S16. Linear sweep voltammograms of RRDE experiment with 0.5 mM **Ph_2Phen^{2+}** and 0.1 M TFAH under air saturation conditions. (A) Uncorrected LSVs at 600 rpm. (B) Corrected LSVs at various rotation rates used for quantification of %H_2O_2.......................................19

Figure S17. (A) Levich and (B) Koutecky-Levich plots from data obtained from linear sweep voltammograms of **Ph_2Phen^{2+}** (0.5 mM) by RRDE with 0.1 M TFAH under air saturation conditions at various rotation rates...20

Electrochemical Analysis with Cl_3AcOH..20

Figure S18. (A) CVs of **Ph_2Phen^{2+}** under Ar (black), O_2 (red) and with 0.1 M Cl_3AcOH under Ar saturation (green). (B) CVs from A and catalytic trace shown (blue) with **Ph_2Phen^{2+}** and 0.1 M Cl_3AcOH under O_2 saturation. 20

*Determination of Effective Overpotential of **Ph_2Phen^{2+}** with Cl_3AcOH*................................21

Figure S19. (A) CVs of **Ph_2Phen^{2+}** under catalytic conditions with 0.1 M Cl_3AcOH at 0.1 V/s (black) and 1 V/s (red)..21

Figure S20. Rinse test of **Ph_2Phen^{2+}** and Cl_3AcOH. 22

Figure S21. (A) CVs of **Ph_2Phen^{2+}** under catalytic conditions with variable **Ph_2Phen^{2+}** concentrations. (B) Logarithm of **Ph_2Phen^{2+}** concentration versus the logarithm of current density from (A)..22

Figure S22. CVs of **Ph_2Phen^{2+}** under catalytic conditions with variable Cl_3AcOH concentrations. 23

Figure S23. (A) CVs of **Ph_2Phen^{2+}** under catalytic conditions with variable O_2 concentrations. (B) Logarithm of O_2 concentration versus the logarithm of current density from (A)............23

Figure S24. (A) CVs of **Ph_2Phen^{2+}** in the presence of 5.7 mM urea•H_2O_2 under Ar and O_2 saturation with added 0.12 M Cl_3AcOH. (B) CVs of **Ph_2Phen^{2+}** in the presence of 5.7 mM urea•H_2O_2 with added 0.12 M Cl_3AcOH under O_2 saturation..24

Figure S25. Linear sweep voltammograms of RRDE experiment with 0.5 mM **Ph_2Phen^{2+}** and 0.1 M Cl_3AcOH under air saturation conditions. (A) Uncorrected LSVs at 600 rpm. (B) Corrected LSVs at various rotation rates used for quantification of %H_2O_2. 24

Figure S26. (A) Levich and (B) Koutecky-Levich plots from data obtained from linear sweep voltammograms of 25

Electrochemical Analysis with Cl_2AcOH..25

Figure S27. (A) CVs of **Ph_2Phen^{2+}** under Ar (black), O_2 (red) and with 0.1 M Cl_2AcOH under Ar saturation (green). (B) CVs from A and catalytic trace shown (blue) with Ph_2Phen^{2+} and 0.1 M Cl_2AcOH under O_2 saturation. 25

Determination of Effective Overpotential of Ph_2Phen^{2+} with Cl_2AcOH................................26

Figure S28. (A) CVs of **Ph_2Phen^{2+}** under catalytic conditions with 0.1 M Cl_2AcOH at 0.1 V/s (black) and 1 V/s (red).......26

Figure S29. Rinse test of **Ph_2Phen^{2+}** and Cl_2AcOH. 27

Figure S30. CVs of **Ph_2Phen^{2+}** under catalytic conditions with variable **Ph_2Phen^{2+}** concentrations. (B) Logarithm of **Ph_2Phen^{2+}** concentration versus the logarithm of current density from (A).......27

Figure S31. (A) CVs of **Ph_2Phen^{2+}** under catalytic conditions with variable Cl_2AcOH concentrations. (B) Logarithm of Cl_2AcOH concentration versus the logarithm of current density from (A).......28

Figure S32. (A) CVs of **Ph_2Phen^{2+}** under catalytic conditions with variable O_2 concentrations. (B) Logarithm of O_2 concentration versus the logarithm of current density from (A).......28

Figure S33. (A) CVs of **Ph_2Phen^{2+}** in the presence of 5.5 mM urea•H_2O_2 under Ar and O_2 saturation with added 0.06 M Cl_2AcOH. (B) CVs of **Ph_2Phen^{2+}** in the presence of 5.7 mM urea•H_2O_2 with added 0.06 M Cl_2AcOH under O_2 saturation 29

Figure S34. Linear sweep voltammograms of RRDE experiment with 0.5 mM **Ph_2Phen^{2+}** and 0.1 M Cl_2AcOH under air saturation conditions. (A) Uncorrected LSVs at 600 rpm. (B) Corrected LSVs at various rotation rates used for quantification of %H_2O_2. 29

Figure S35. (A) Levich and (B) Koutecky-Levich plots from data obtained from linear sweep voltammograms of **Ph_2Phen^{2+}** (0.5 mM) by RRDE with 0.1 M Cl_2AcOH under air saturation conditions at various rotation rates.......30

Electrochemical Analysis with ClAcOH 30

Figure S36. (A) CVs of **Ph_2Phen^{2+}** under Ar (black), O_2 (red) and with 0.1 M ClAcOH under Ar saturation (green). (B) CVs from A and catalytic trace shown (blue) with **Ph_2Phen^{2+}** and 0.1 M ClAcOH under O_2 saturation. 30

*Determination of Effective Overpotential of **Ph_2Phen^{2+}** with ClAcOH* 31

Figure S37. (A) CVs of **Ph_2Phen^{2+}** under catalytic conditions with 0.1 M ClAcOH at 0.1 V/s (black) and 1 V/s (red).......31

Figure S38. Rinse test of **Ph_2Phen^{2+}** and ClAcOH. 32

Figure S39. CVs of **Ph_2Phen^{2+}** under catalytic conditions with variable **Ph_2Phen^{2+}** concentrations. (B) Logarithm of **Ph_2Phen^{2+}** concentration versus the logarithm of current density from (A).......32

Figure S40. (A) CVs of **Ph_2Phen^{2+}** under catalytic conditions with variable ClAcOH concentrations. (B) Logarithm of ClAcOH concentration versus the logarithm of current density from (A).......33

Figure S41. (A) CVs of **Ph_2Phen^{2+}** under catalytic conditions with variable O_2 concentrations. (B) Logarithm of O_2 concentration versus the logarithm of current density from (A).......33

Figure S42. (A) CVs of **Ph_2Phen^{2+}** in the presence of 5.7 mM urea•H_2O_2 under Ar and O_2 saturation with added 0.1 M ClAcOH. (B) CVs of **Ph_2Phen^{2+}** in the presence of 5.7 mM urea•H_2O_2 with added 0.1 M ClAcOH under O_2 saturation. 34

Figure S43. Linear sweep voltammogram of RRDE experiment with 0.5 mM **Ph_2Phen^{2+}** and 0.1 M ClAcOH under air saturation conditions at 200 rpm. 34

Figure S44. CVs under catalytical conditions with variable **Ph_2Phen^{2+}** concentrations and scan rates in order to achieve an S-shaped catalytic wave with TFAH as a proton source. 35

Figure S45. CVs under catalytic conditions with variable **Ph_2Phen^{2+}** concentrations and scan rates in order to achieve an S-shaped catalytic wave with Cl3AcOH as a proton source. 35

Figure S46. CVs under catalytic conditions with variable **Ph_2Phen^{2+}** concentrations and scan rates in order to achieve an S-shaped catalytic wave with Cl2AcOH as a proton source. 35

Figure S47. CVs under catalytical conditions with variable **Ph_2Phen^{2+}** concentrations and scan rates in order to achieve an S-shaped catalytic wave with ClAcOH as a proton source. 36

Table S2. Calculated i_{cat}/i_p and TOF values for variable Ph_2Phen^{2+} concentration at all catalytic conditions..36

Stopped-Flow Kinetic Analysis of Ph_2Phen^{2+} 36

Stopped-Flow with TFAH 37

Figure S48. (A) Change in absorbance at 780 nm over time as a result of the formation of $[Cp^*{}_2Fe]^+$ by ORR catalyzed by **Ph_2Phen^{2+}** with TFAH (black trace), example of 2Exp + Mx + C fit in Kinetic Studio 4.0 (red trace), and residual fit (blue trace). (B) Black trace from (A) with TFAH only control (no **Ph_2Phen^{2+}** present). 37

Figure S49. Calculated R_{fit}/n_{cat} values from stopped-flow spectrochemical experiments with TFAH, O_2, and $Cp^*{}_2Fe$ with varying **Ph_2Phen^{2+}** concentration. 37

Figure S50. Calculated R_{fit}/n_{cat} values from stopped-flow spectrochemical experiments with **Ph_2Phen^{2+},** O_2, and $Cp^*{}_2Fe$ with varying TFAH concentration. 38

Figure S51. Calculated R_{fit}/n_{cat} values from stopped-flow spectrochemical experiments with **Ph_2Phen^{2+}**, TFAH, and $Cp^*{}_2Fe$ with varying O_2 concentration. 38

Figure S52. Calculated R_{fit}/n_{cat} values from stopped-flow spectrochemical experiments with **Ph_2Phen^{2+}**, TFAH, and O_2 with varying $Cp^*{}_2Fe$ concentration. 39

Comparison of ORR and H_2O_2RR with TFAH 39

Stopped-Flow with Cl_3AcOH 40

Figure S54. Change in absorbance at 780 nm over time as a result of the formation of $[Cp^*{}_2Fe]^+$ by ORR catalyzed by **Ph_2Phen^{2+}** with Cl_3AcOH (black trace), example of 2Exp + Mx + C fit in Kinetic Studio 4.0 (red trace), and residual fit (blue trace). (B) Black trace from (A) with Cl_3AcOH only control (no **Ph_2Phen^{2+}** present)..40

Figure S55. Calculated R_{fit}/n_{cat} values from stopped-flow spectrochemical experiments with Cl_3AcOH, O_2, and $Cp^*{}_2Fe$ with varying **Ph_2Phen^{2+}** concentration. 40

Figure S56. Calculated R_{fit}/n_{cat} values from stopped-flow spectrochemical experiments with **Ph_2Phen^{2+}**, O_2, and $Cp^*{}_2Fe$ with varying Cl_3AcOH concentration. 41

Figure S57. Calculated R_{fit}/n_{cat} values from stopped-flow spectrochemical experiments with **Ph_2Phen^{2+}**, Cl_3AcOH, and $Cp^*{}_2Fe$ with varying O_2 concentration. 41

Figure S58. Calculated R_{fit}/n_{cat} values from stopped-flow spectrochemical experiments with **Ph_2Phen^{2+}**, Cl_3AcOH, and O_2 with varying $Cp^*{}_2Fe$ concentration. 42

Comparison of ORR and H_2O_2RR with Cl_3AcOH 42

Stopped-Flow with Cl_2AcOH 43

Figure S60. (A) Change in absorbance at 780 nm over time as a result of the formation of $[Cp^*_2Fe]^+$ by ORR catalyzed by **Ph_2Phen^{2+}** with Cl_2AcOH (black trace), example of 2Exp + Mx + C fit in Kinetic Studio 4.0 (red trace), and residual fit (blue trace). 43

Figure S61. Calculated R_{fit}/n_{cat} values from stopped-flow spectrochemical experiments with Cl_2AcOH, O_2, and Cp^*_2Fe with varying **Ph_2Phen^{2+}** concentration. 43

Figure S62. Calculated R_{fit}/n_{cat} values from stopped-flow spectrochemical experiments with **Ph_2Phen^{2+}**, O_2, and Cp^*_2Fe with varying Cl_2AcOH concentration. 44

Figure S63. Calculated R_{fit}/n_{cat} values from stopped-flow spectrochemical experiments with **Ph_2Phen^{2+}**, Cl_2AcOH, and Cp^*_2Fe with varying O_2 concentration. 44

Figure S64. Calculated R_{fit}/n_{cat} values from stopped-flow spectrochemical experiments with **Ph_2Phen^{2+}**, Cl_2AcOH, and O_2 with varying Cp^*_2Fe concentration. 45

Comparison of ORR and H_2O_2RR with Cl_2AcOH 45

Stopped-Flow with ClAcOH 46

Figure S66. Change in absorbance at 780 nm over time as a result of the formation of $[Cp^*_2Fe]^+$ by ORR catalyzed by **Ph_2Phen^{2+}** with ClAcOH (black trace), example of 2Exp + Mx + C fit in Kinetic Studio 4.0 (red trace), and residual fit (blue trace). 46

Figure S67. Calculated R_{fit}/n_{cat} values from stopped-flow spectrochemical experiments with ClAcOH, O_2, and Cp^*_2Fe with varying **Ph_2Phen^{2+}** concentration. 46

Figure S68. Calculated R_{fit}/n_{cat} values from stopped-flow spectrochemical experiments with **Ph_2Phen^{2+}**, O_2, and Cp^*_2Fe with varying ClAcOH concentration 47

Figure S69. Calculated R_{fit}/n_{cat} values from stopped-flow spectrochemical experiments with **Ph_2Phen^{2+}**, ClAcOH, and Cp^*_2Fe with varying O_2 concentration. 47

Figure S70. Calculated R_{fit}/n_{cat} values from stopped-flow spectrochemical experiments with **Ph_2Phen^{2+}**, ClAcOH, and O_2 with varying Cp^*_2Fe concentration. 48

Comparison of ORR and H_2O_2RR with ClAcOH 48

Spectrochemical Analysis 49

ORR Selectivity 49

Table S3. Summary of ORR selectivity by **Ph_2Phen^{2+}** with each acid.[a] 49

Figure S72. H_2O_2 product quantification of ORR by **Ph_2Phen^{2+}** with TFAH after 90 s. 49

Figure S73. H_2O_2 product quantification of ORR by **Ph_2Phen^{2+}** with Cl_3AcOH after 90 s. ..50

Figure S74. H_2O_2 product quantification of ORR by **Ph_2Phen_{2+}** with Cl_2AcOH after 2 min. 50

Figure S75. H_2O_2 product quantification of ORR by **Ph_2Phen^{2+}** with ClAcOH after 3 min. .51

Figure S76. H_2O_2 product quantification of ORR by **Ph_2Phen^{2+}** with ClAcOH after 30 s, 2 min, and 5 min. 51

Table S4. Summary of ORR selectivity of **Ph_2Phen^{2+}** with ClAcOH at various time points from **Figure S76** 51

H_2O_2 Stability Control Studies 52

Table S5. Summary of H_2O_2 Recovery for Stability Control Studies by **Ph_2Phen^{2+}** with each acid. 52

Figure S77. Stability test of urea•H_2O_2 in the presence of **Ph_2Phen^{2+}**, TFAH, and O_2........52

Figure S78. Stability test of urea•H_2O_2 in the presence of **Ph_2Phen^{2+}**, Cl_3AcOH, and O_2...53

Figure S79. Stability test of urea•H_2O_2 in the presence of **Ph_2Phen^{2+}**, Cl_2AcOH, and O_2...53

Figure S80. Stability test of urea•H_2O_2 in the presence of **Ph_2Phen^{2+}**, ClAcOH, and O_2....54

Computational Methods 54

Figure S81. Comparison of computational pathways to H_2O_2 from the radical-ion pair. 55

Figure S82. Comparison of computational pathways for acid-mediated dimer cleavage and H_2O_2 production for the strongest (TFAH) and weakest (ClAcOH) acids. 56

^{1}H-NMR Studies..56

Figure S83. ^{1}H NMR spectra of **$Ph_2Phen(PF_6)_2$** (red) in the presence of Cp^*_2Fe under N_2 (green) and exposed to air (blue). Conditions: 3.2 mM [**$Ph_2Phen(PF_6)_2$**], 4 mM [Cp^*_2Fe]; MeCN-d_3; Varian 600 MHz. 57

Figure S84. ^{1}H NMR spectra of **$Ph_2Phen(PF_6)_2$** (red) in the presence of Cp^*_2Fe exposed to air (green) and TFAH under N_2 (teal) and exposed to air (blue). Conditions: 3.2 mM [**$Ph_2Phen(PF_6)_2$**], 4 mM [Cp^*_2Fe], 4 mM [TFAH]; MeCN-d_3; Varian 600 MHz. 58

Figure S85. ^{1}H NMR spectra of **$Ph_2Phen(PF_6)_2$** in the presence of Cp^*_2Fe and ClAcOH under N_2 (color) and exposed to air (color). Conditions: 3.2 mM [**$Ph_2Phen(PF_6)_2$**], 4 mM [Cp^*_2Fe], 4 mM [ClAcOH]; MeCN-d_3; Varian 600 MHz. 59

Figure S86. ^{1}H-NMR spectra of **$Ph_2Phen(PF_6)_2$** (red) in the presence of Cp^*_2Fe and urea•H_2O_2 (green) with TFAH (teal) and ClAcOH (purple) under N_2 atmosphere. Conditions: 3.7 mM [**$Ph_2Phen(PF_6)_2$**], 4 mM [Cp^*_2Fe] if present, 5.3 mM [urea•H_2O_2] if present, 4 mM [TFAH] if present, 5.3 mM [ClAcOH] if present; N_2 atmosphere; MeCN-d_3; Varian 600 MHz.................60

Table S7. Summary of Best Performing Previously Reported Metal-Free ORR Systems. 61

X-Ray Crystallographic Details..61

Refinement details for Ph_2Phen^{2+} 61

Table S8. Crystal data and structure refinement for **Ph_2Phen^{2+}**...61

Experimental Methods

General Considerations. All chemicals and solvents (ACS or HPLC grade) were commercially available and used as received unless otherwise indicated. For all air-sensitive reactions and electrochemical experiments, HPLC-grade solvents were obtained as anhydrous and air-free from a PPT Glass Contour Solvent Purification System. Gas cylinders were obtained from Praxair (Ar as 5.0; O2 as 4.0) and passed through activated molecular sieves prior to use. Gas mixing for variable concentration experiments was accomplished using a gas proportioning rotameter from Omega Engineering. UV-vis absorbance spectra were obtained on a Cary 60 from Agilent. An Anton-Parr Multiwave Pro SOLV, NXF-8 microwave reactor was used for microwave syntheses. The concentration of O_2 saturation in MeCN is reported to be 8.1 mM and the saturation concentration in MeCN with added electrolyte to be 6.3 mM.[1]

Synthesis and Characterization

Synthesis of 1,11-diphenyl-6,7-dihydro-5H-[1,4]diazepino[1,2,3,4-lmn][1,10]phenanthroline-4,8-diium bromide (**Ph_2Phen^{2+}**). In a round bottom flask, 4,7-diphenyl-1,10-phenanthroline (2.0 g, 6.0 mmol) was added to 20 mL of toluene. The solution was stirred and brought to 70°C. Once the temperature was reached, 1,3-dibromopropane (2.8 mL, 28 mmol) was added. The solution was brought to reflux and allowed to stir overnight. The resulting bright orange solid was filtered, washed with hexanes, and recrystallized from hot dichloromethane to obtain 0.80 g (25% yield) of pure yellow-orange solid. ^{1}H-NMR (MeOD-d_4, 600 MHz Varian): δ 9.75 (d, 2H), 8.55 (d, 2H), 8.43 (s, 2H), 7.79 (m, 10H), 5.01 (t, 4H), 3.44 (quint, 2H). ^{13}C{^{1}H}-NMR (MeOD-d_4, 150 MHz Varian): δ 158.66, 149.77, 134.79, 134.27, 132.22, 131.09, 130.31, 129.23, 127.97, 126.90, 60.01, 30.81. Elemental analysis calculated for $C_{27}H_{22}Br_2N_2$•1/3CH_2Cl_2: C 58.35, H 4.06, N 4.98; Found: C 58.10, H 3.91, N 4.95.

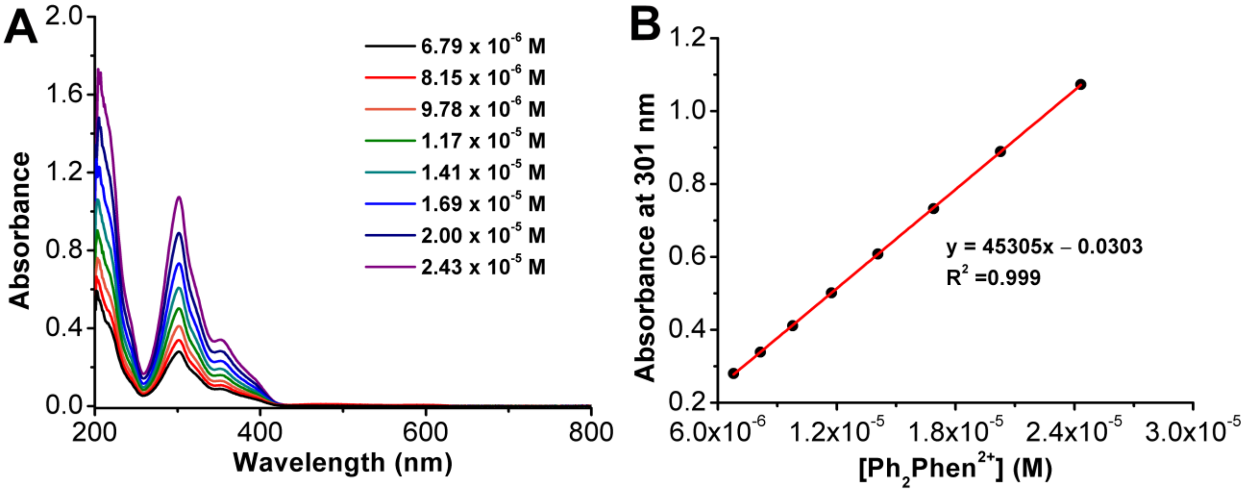

Figure S1. (A) UV-vis serial dilution absorbance data from **Ph_2Phen^{2+}** in MeCN solution. Conditions: varying concentration; quartz cell with 1 cm pathlength. (B) Plot of absorbance concentration (M) for **Ph_2Phen^{2+}** in MeCN solution at 301 nm (45305 M^{-1} cm^{-1}); R^2=0.999. All: λ_{max} = 351 nm (14411 $M^{-1}cm^{-1}$), 394 nm (5760 $M^{-1}cm^{-1}$).

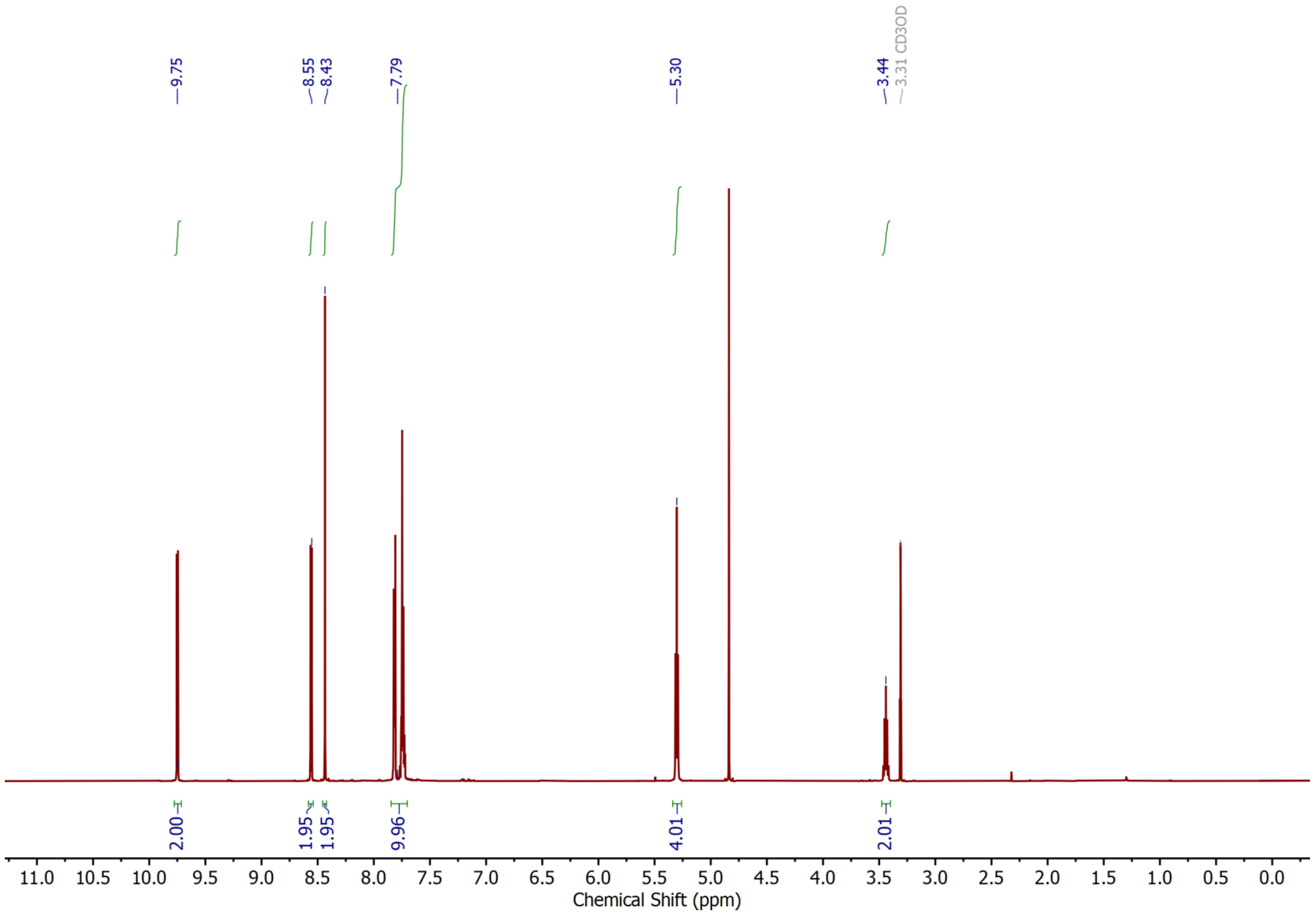

Figure S2. ^{1}H-NMR spectrum of **Ph_2Phen^{2+}** in MeOD-d_4; Varian 600 MHz.

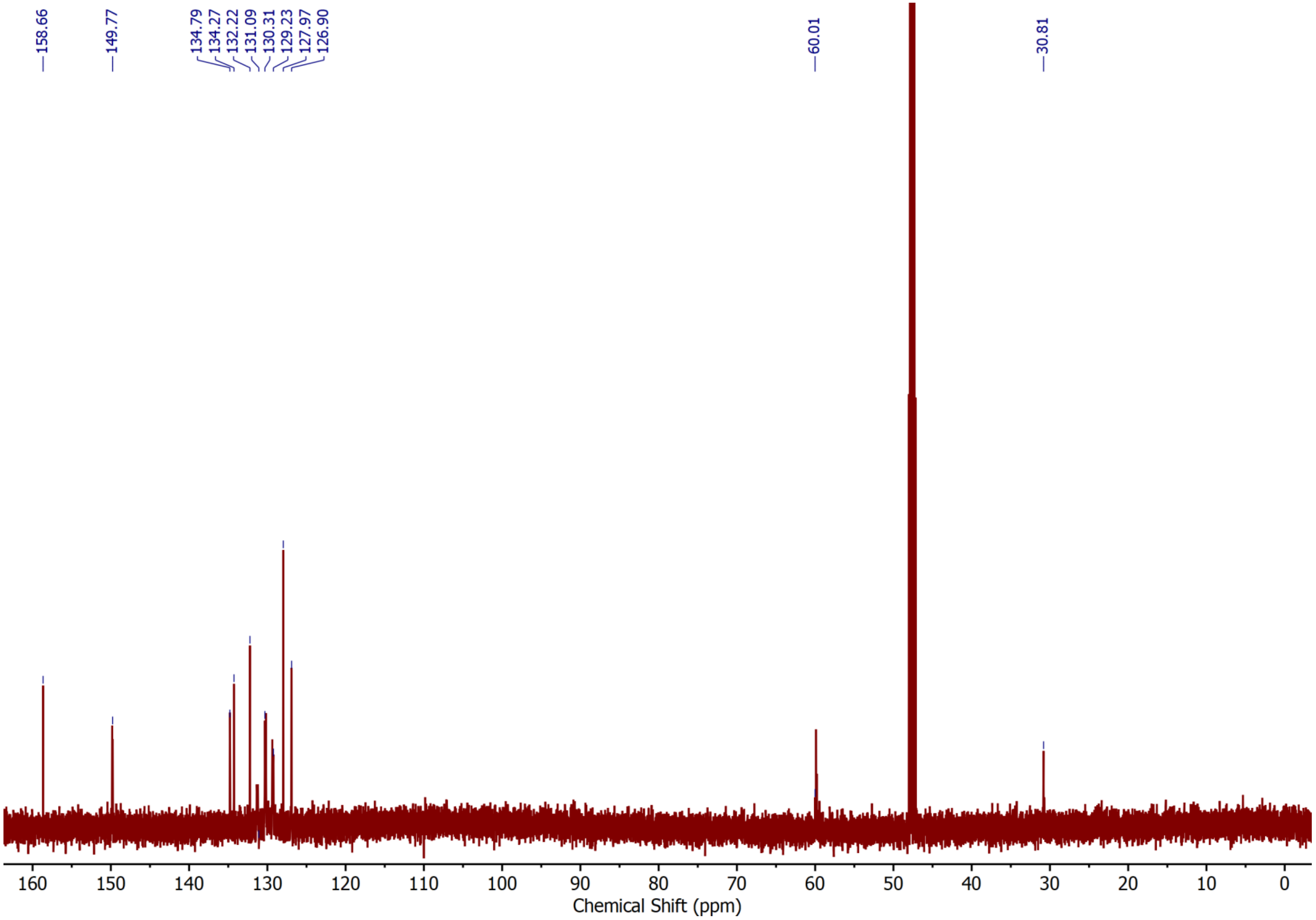

Figure S3. $^{13}C\{^1H\}$ NMR of **Ph_2Phen^{2+}** in MeOD-d_4; Varian 150 MHz.

Synthesis of 1,11-diphenyl-6,7-dihydro-5H-[1,4]diazepino[1,2,3,4-lmn][1,10]phenanthroline-4,8-diium hexafluorophosphate (**$Ph_2Phen(PF_6)_2$**). In a vial, 0.25 g (0.37 mmol) of the dibromide salt of **Ph_2Phen^{2+}** (0.25 g, 0.37 mmol) was dissolved in a minimal amount of methanol. A saturated solution of NH_4PF_6 (0.3 g, 1.86 mmol in minimal methanol) was passed through a syringe filter and added to the **Ph_2Phen^{2+}** solution. **$Ph_2Phen(PF_6)_2$** immediately precipitated out of solution. The light orange solid was filtered and rinsed with excess methanol to obtain pure product (0.15 g, 61% yield). Elemental analysis calculated for $C_{27}H_{22}N_2P_2F_{12}$: C 48.81, H 3.34, N 4.22; Found: C 48.05, H 3.23, N 4.13.

Estimation of pK_a Values for Cl_3AcOH, Cl_2AcOH, and ClAcOH in MeCN

The pK_as of Cl_3AcOH, Cl_2AcOH, and ClAcOH in MeCN were estimated from pK_a(H_2O) using a linear scaling relationship reported by Leito and coworkers, described in **Eq S1**.[2] Where (X–CO) is the number of carbonyl groups attached directly to the acidity center, nC is the number of C atoms in the molecule, and MW is the molecular weight.

$$pK_a(H_2O) = pK_a(MeCN) \times 0.55 - (X - CO) \times 2.2 + nC \times 0.13 + MW \times 0.0017 - 6.5 \qquad \textbf{Eq S1}$$

Table S1. Summary of calculated pK_a values in MeCN.

Acid	pK_a(H_2O)	X–CO	nC	MW (g/mol)	pK_a(MeCN)
Cl_3AcOH	0.65	1	2	163.38	16.0
Cl_2AcOH	1.29	1	2	128.94	17.3
ClAcOH	2.86	1	2	94.5	20.3

Electrochemical Analysis of Ph_2Phen^{2+}

Electrochemistry

Electroanalytical experiments were performed using Metrohm Autolab PGSTAT302N and BioLogic SP-50 potentiostats. RRDE experiments were performed using BioLogic VSP Bipotentiostat with a Pine Research MSR Rotator. Glassy carbon working (⌀ = 3 mm) and non-aqueous silver/silver chloride pseudoreference electrodes behind PTFE tips were obtained from CH Instruments. The pseudoreference electrodes were obtained by depositing chloride on bare silver wire in 10% HCl at oxidizing potentials and stored in a 0.1 M tetrabutylammonium hexafluorophosphate solution in acetonitrile in the dark prior to use. The counter electrode was a glassy carbon rod (⌀ = 3 mm). All CV experiments were performed in a modified scintillation vial (20 mL volume) as a single-chamber cell with a cap modified with ports for all electrodes and a sparging needle. Tetrabutylammonium hexafluorophosphate ($TBAPF_6$) was purified by recrystallization from ethanol and dried in a vacuum oven before being stored in a desiccator. All data were referenced to an internal ferrocene standard (ferrocenium/ferrocene reduction potential under stated conditions) unless otherwise specified. All voltammograms were corrected for internal resistance. Ferrocene was purified by sublimation prior to use. In the event that the presence of electrochemical features precluded ferrocene addition, ferrocene was added to the electrochemical cell at the end of analysis for reference.

Rotating Ring-Disk Electrode Methods

Description of Au Ring Roughening Procedure. The Au ring electrode was roughened according to a previously reported method.[3] The electrodes were polished first on a felt polishing pad with 0.3 micron alumina, then with 0.05 micron alumina and rinsed with water and ethanol. Cyclic voltammograms were obtained in 0.5 M H_2SO_4 by scanning from 0 to 1.6 V vs. Ag/AgCl at 100 mV/s, then at 20 mV/s for an additional 2 cycles to obtain the pre-roughening, surface oxide

reduction charge. The electrode was then pulsed between 2.4 and 0.2 V vs Ag/AgCl for 2.4 ms each and repeated for 250,000 cycles. Bubbles formed during electrolysis pulses were dislodged by contacting with a large bubble from a glass pipette. After electrolysis, the electrode was held at 0.3 V vs. Ag/AgCl for 2 minutes and the roughening was evaluated by CV.

Description of RRDE Collection Efficiency. The collection efficiency was determined as previously reported.[4-6] Conditions: Air saturation, 0.1 M $TBAPF_6$, 0.5 mM ferrocene in MeCN, glassy carbon disk electrode (5 mm), roughened Au ring electrode, glassy carbon rod counter electrode, Ag/AgCl pseudoreference electrode; scan rate 0.01 V/s. To calculate the collection efficiency of the RRDE, the ratio of the ring current (i_r) to the disk current (i_d) at each rotation rate was used to determine $N_{empirical}$ (**Eq S2**). The $N_{empirical}$ value at each rotation rate was multiplied by a factor of 100 to determine the collection efficiency % at each rotation rate (~23%).

$$N_{empirical} = \frac{i_{ring\ corrected}}{i_{disk\ corrected}} \qquad \textbf{(Eq S2)}$$

RRDE Experiments. Conditions: Performed under Ar and air saturation conditions, 0.1 M $TBAPF_6$, 0.5 mM **Ph_2Phen^{2+}**, 0.1 M acid, glassy carbon disk electrode (5 mm diameter), roughened Au ring electrode, glassy carbon rod counter electrode, Ag/AgCl pseudoreference electrode; scan rate 0.01 V/s.

The solution was sparged with air until saturation was achieved. **Ph_2Phen^{2+}** (0.5 mM) was dissolved in MeCN and 0.1 M acid was added. A standard CV was taken of the solution to confirm the potential window to be used for the experiment. The roughened Au ring was set to +1.0 V. LSVs were obtained for various rotation rates between 600 and 2200 under the described conditions. In between each scan, the solution was sparged for 2 minutes. The reproducibility of scans was confirmed by repeating scans at the same rotation rate, producing exact overlays. The same procedure was repeated and the roughened Au ring was set to +1.2 V. The ring current (i_r) was corrected for by subtracting limiting current observed at the ring when it was set at +1.0 V. Levich analysis was used to verify the suitability of the quantification method (**Figures S17**, **S26**, **35**).

The arithmetic mean of the number of electrons received by O_2 (n_{cat}) during the ORR was calculated from the disk current (i_d) and ring current (i_r) according to **Eq S3:**[7]

$$n_{cat} = 4 \times \frac{i_d}{i_d + \frac{i_r}{N_{empirical}}} \qquad \textbf{(Eq S3)}$$

The H_2O_2 ratio (p) is defined as the fraction of O_2 reduced to H_2O_2 and relates to n_{cat} by **Eq S4**:

$$n_{cat} = 4 - 2p \qquad \textbf{(Eq S4)}$$

Multiplying p by 100% provides the %H_2O_2 selectivity of the ORR.

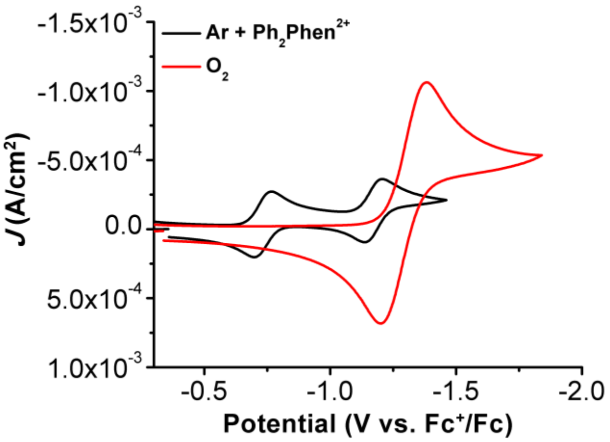

Figure S4. CV of $\mathbf{Ph_2Phen^{2+}}$ under Ar (black) compared to O_2 in a blank MeCN solution (red). Conditions: 1 mM $\mathbf{Ph_2Phen^{2+}}$, 0.1 M $TBAPF_6$/MeCN; glassy carbon working electrode, glassy carbon rod counter electrode, Ag/AgCl pseudoreference electrode; referenced to Fc^+/Fc internal standard; 100 mV/s scan rate.

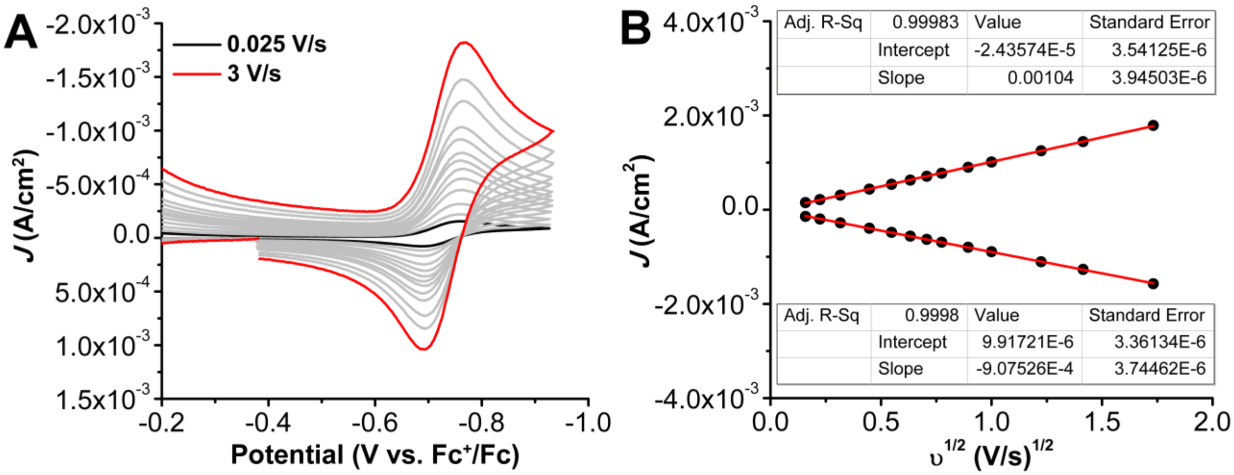

Figure S5. (A) CVs of $\mathbf{Ph_2Phen^{2+}}$ under Ar saturation at various scan rates. (B) Square root of scan rate versus current density in (A). Conditions: 1 mM $\mathbf{Ph_2Phen^{2+}}$, 0.1 M $TBAPF_6$/MeCN; glassy carbon working electrode, glassy carbon rod counter electrode, Ag/AgCl pseudoreference electrode; referenced to an internal ferrocene standard; scan rates: 0.025, 0.05, 0.1, 0.2, 0.3, 0.4, 0.5, 0.6, 0.8, 1, 1.5, 2, 3 V/s.

The diffusion coefficient of $\mathbf{Ph_2Phen^{2+}}$ was calculated using the slope from **Figure S5B** and **Eq S5**, where i_p is the current (A), n is the number of electrons, A is the area of the electrode (cm^2), C is the concentration (mol/cm^2), v is the scan rate (V/s), and D is the diffusion coefficient (cm^2/s)

$$i_p = (2.69 \times 10^5) n^{\frac{3}{2}} A C D^{\frac{1}{2}} v^{\frac{1}{2}} \qquad \textbf{Eq S5}$$

$$D = \frac{(slope)^2}{n^3 C^2 (2.69 \times 10^5)^2}$$

$$D = 1.14 \times 10^{-5}\ cm^2 \cdot s^{-1}$$

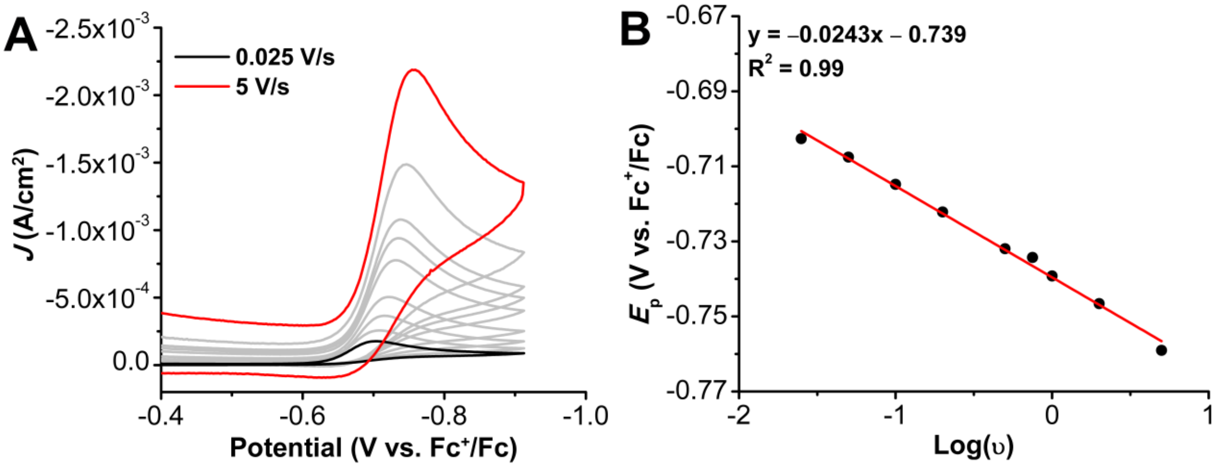

Figure S6. (A) CVs of **Ph_2Phen^{2+}** under O_2 saturation at various scan rates. (B) Logarithm of the scan rate versus the reduction peak potential in (A). Conditions: 1 mM **Ph_2Phen^{2+}**, 0.1 M $TBAPF_6$/MeCN; glassy carbon working electrode, glassy carbon rod counter electrode, Ag/AgCl pseudoreference electrode; referenced to an internal ferrocene standard; scan rates: 0.025, 0.05, 0.1, 0.2, 0.3, 0.4, 0.5, 0.6, 0.8, 1, 1.5, 2, 3, 4, 5 V/s.

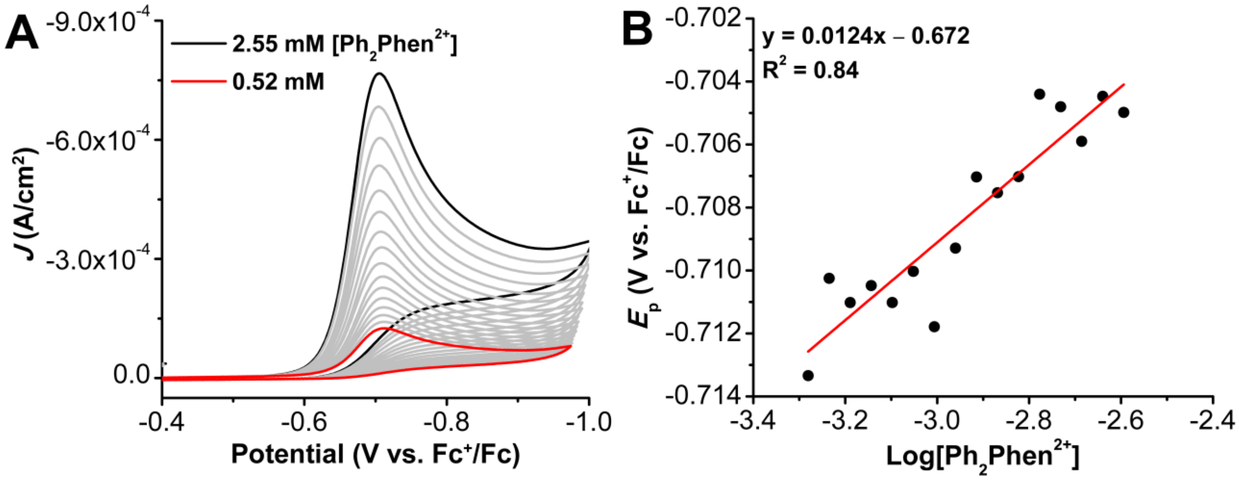

Figure S7. (A) CVs of **Ph_2Phen^{2+}** under O_2 saturation at varying concentrations. (B) Logarithm of **Ph_2Phen^{2+}** concentration versus the reduction peak potential in (A). Conditions: varying **Ph_2Phen^{2+}** concentration, 0.1 M $TBAPF_6$/MeCN; glassy carbon working electrode, glassy carbon rod counter electrode, Ag/AgCl pseudoreference electrode; referenced to an internal ferrocene standard; 100 mV/s scan rate. [**Ph_2Phen^{2+}**]: 2.55, 2.23, 2.06, 1.86, 1.67, 1.50, 1.35, 1.22, 1.10, 0.986, 0.888, 0.799, 0.719, 0.647, 0.582, 0.524 mM

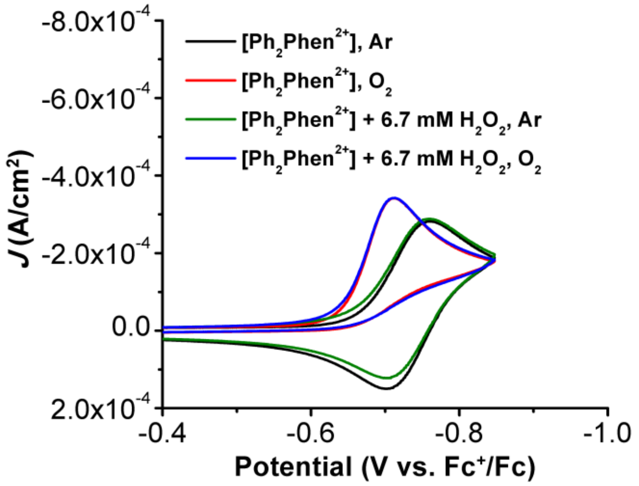

Figure S8. CVs of $\mathbf{Ph_2Phen^{2+}}$ with and without added 6.7 mM urea•H_2O_2 under Ar and O_2 saturation. Conditions: 1 mM $\mathbf{Ph_2Phen^{2+}}$, 0.1 M $TBAPF_6$/MeCN; 100 mV/s; glassy carbon working electrode, glassy carbon counter electrode, Ag/AgCl pseudoreference electrode; referenced to an internal ferrocene standard.

Electrochemical Analysis with TFAH

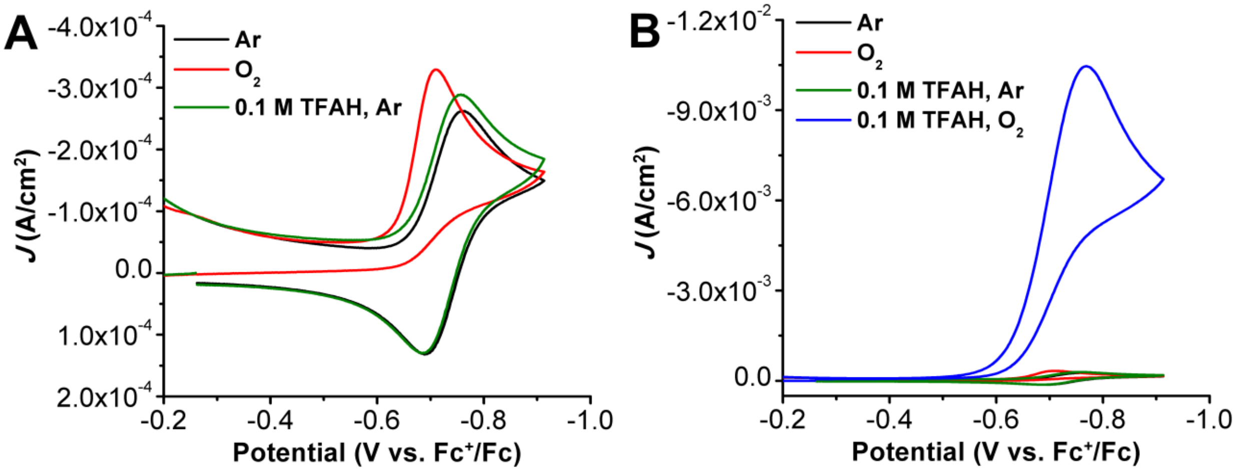

Figure S9. (A) CVs of $\mathbf{Ph_2Phen^{2+}}$ under Ar (black), O_2 (red) and with 0.1 M TFAH under Ar saturation (green). (B) CVs from A and catalytic trace shown (blue) with Ph_2Phen^{2+} and 0.1 M TFAH under O_2 saturation. Conditions: 1 mM $\mathbf{Ph_2Phen^{2+}}$, 0.1 M $TBAPF_6$/MeCN; glassy carbon working electrode, glassy carbon rod counter electrode, Ag/AgCl pseudoreference electrode; referenced to an internal ferrocene standard; 100 mV/s scan rate.

Utilizing the pK_a (12.65) and log(K_{AHA}) (3.9) values for TFAH in MeCN, we have corrected the reported standard reduction potentials for ORR using **Eqs S6 & S7**.

$$E^0_{O2/H2O} = 1.21 - 0.0592pK_a + \frac{2.30RT}{4F}\log(4K_{AHA})\ V\ vs. Fc^+/Fc \qquad \textbf{Eq (S6)}$$

$$E^0_{O2/H2O}(MeCN, TFAH) = \ 0.53\ V\ vs. Fc^+/Fc$$

$$E^0_{O2/H2O2} = 0.68 - 0.0592pK_a + \frac{2.30RT}{2F}\log(2K_{AHA})\ V\ vs. Fc^+/Fc \qquad \textbf{Eq (S7)}$$

$$E^0_{O2/H2O2}(MeCN, TFAH) = \ +0.06\ V\ vs. Fc^+/Fc$$

where R is the ideal gas law (8.314 J·K^{-1}·mol^{-1}); T is the temperature (298 K); F is Faraday's constant (96485 C·mol^{-1}); K_{AHA} is the reported homoconjugation equilibrium constant for TFAH in MeCN (log K_{AHA} = 3.9). The effective overpotential was calculated according to **Eqs S8 & S9**. Where E_{app} is the $E_{1/2}$ of Ph_2Phen^{2+} with 0.1 M TFAH (**Figure S9**).

$$\eta = \left|E_{app} - E^0_{O2/H2O}\right| = |-0.72 - 0.53| = 1.25\ V \qquad \textbf{Eq (S8)}$$

$$\eta = \left|E_{app} - E^0_{O2/H2O2}\right| = |-0.72 - 0.06| = 0.78\ V \qquad \textbf{Eq (S9)}$$

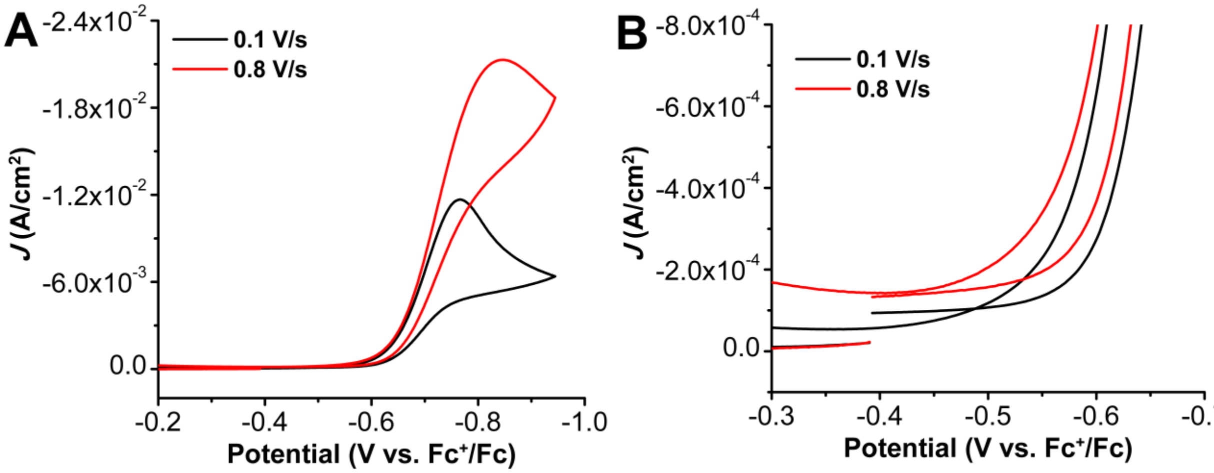

Figure S10. (A) CVs of **Ph_2Phen^{2+}** under catalytic conditions with 0.13 M TFAH at 0.1 V/s (black) and 0.8 V/s (red) (B) CVs from A to emphasize observed cross-tracing. Conditions: 1 mM **Ph_2Phen^{2+}**, 0.13 M TFAH, 0.1 M $TBAPF_6$/MeCN; glassy carbon working electrode, glassy carbon rod counter electrode, Ag/AgCl pseudoreference electrode; referenced to an internal ferrocene standard.

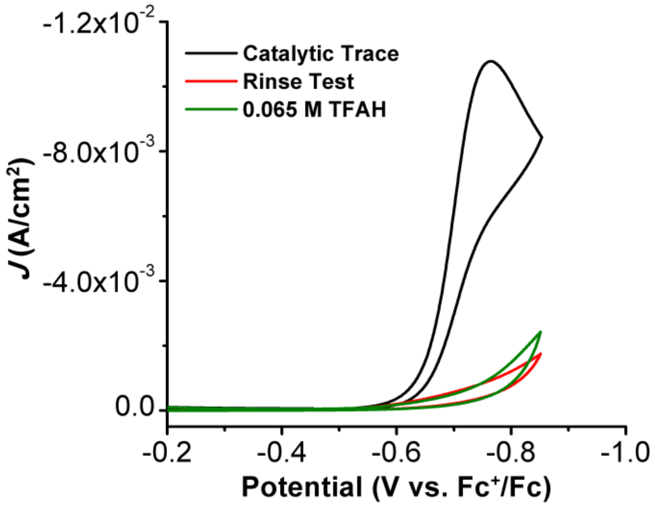

Figure S11. Rinse test of **Ph_2Phen^{2+}** and TFAH. **Ph_2Phen^{2+}** under catalytic conditions (black trace), rinse test (red trace), and CV of TFAH under O_2 saturation (green trace). Conditions: 1 mM **Ph_2Phen^{2+}**, 0.12 M Cl_3AcOH, 0.1 M $TBAPF_6$/MeCN, O_2 saturation; 100 mV/s; glassy carbon working electrode, glassy carbon counter electrode, Ag/AgCl pseudoreference electrode; referenced to an internal ferrocene standard.

To establish mechanistic information under electrochemical conditions, variable concentration studies were performed, analysis was adapted from Sathrum and Kubiak *J. Phys. Chem. Lett.* **2011**, *2*, 2372.[8] Where, *F* is Faraday's constant, *A* is the electrode area, [Q] is the substrate concentration, k_{cat} is the catalytic rate, *D* is the diffusion constant of the catalyst, [cat] is the concentration of the catalyst and n_{cat} is the number of electrons in the catalytic process.

$$i_{cat} = n_{cat}FA[cat](Dk_{cat}[Q]^{y})^{1/2}$$

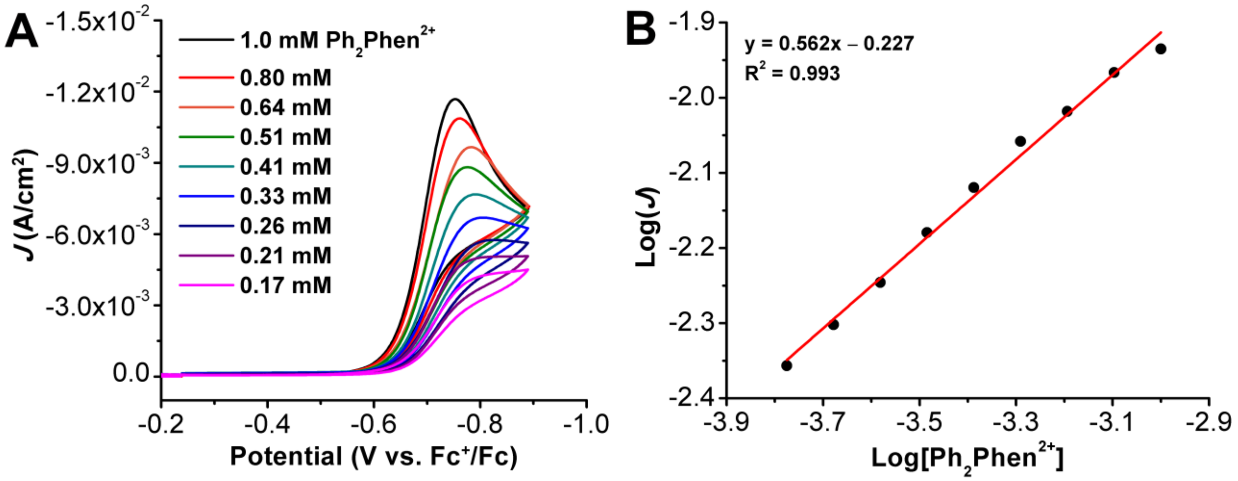

Figure S12. (A) CVs of **Ph_2Phen^{2+}** under catalytic conditions with variable **Ph_2Phen^{2+}** concentrations. (B) Logarithm of **Ph_2Phen^{2+}** concentration versus the logarithm of current density from (A). Conditions: 0.1 M TFAH, 0.1 M $TBAPF_6$/MeCN; 100 mV/s; glassy carbon working electrode, glassy carbon counter electrode, Ag/AgCl pseudoreference electrode; referenced to an internal ferrocene standard.

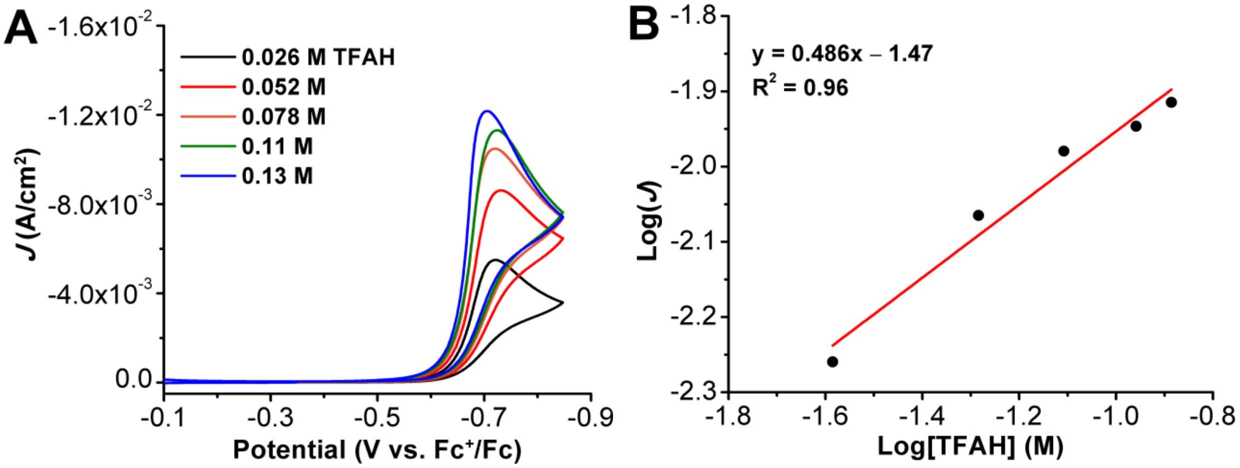

Figure S13. (A) CVs of **Ph_2Phen^{2+}** under catalytic conditions with variable TFAH concentrations. (B) Logarithm of TFAH concentration versus the logarithm of current density from (A). Conditions: 1 mM **Ph_2Phen^{2+}**, 0.1 M $TBAPF_6$/MeCN, O_2 saturation; 100 mV/s; glassy carbon working electrode, glassy carbon counter electrode, Ag/AgCl pseudoreference electrode; referenced to an internal ferrocene standard.

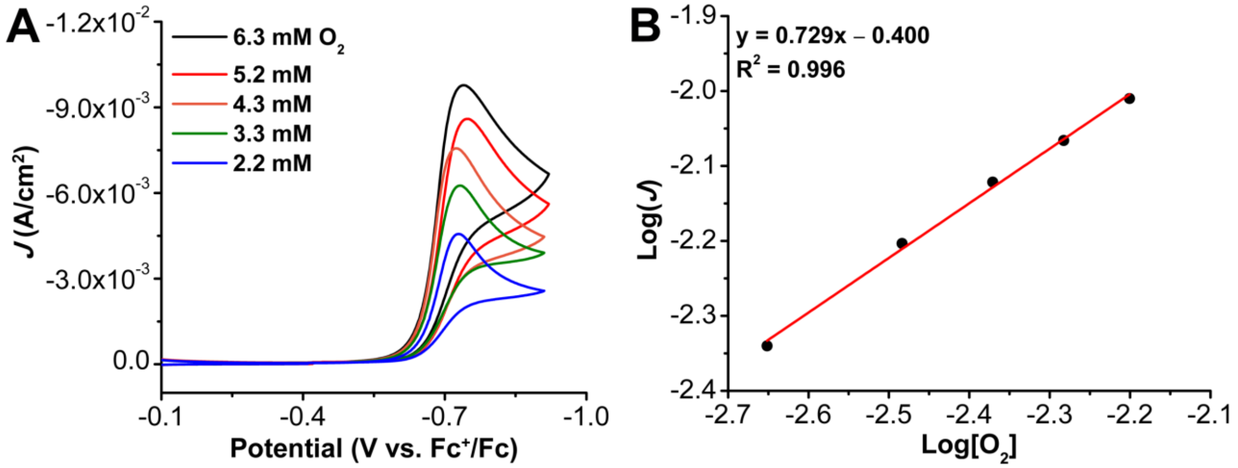

Figure S14. (A) CVs of **Ph_2Phen^{2+}** under catalytic conditions with variable O_2 concentrations. (B) Logarithm of O_2 concentration versus the logarithm of current density from (A). Conditions: 1 mM **Ph_2Phen^{2+}**, 0.1 M TFAH, 0.1 M $TBAPF_6$/MeCN; 100 mV/s; glassy carbon working electrode, glassy carbon counter electrode, Ag/AgCl pseudoreference electrode; referenced to an internal ferrocene standard.

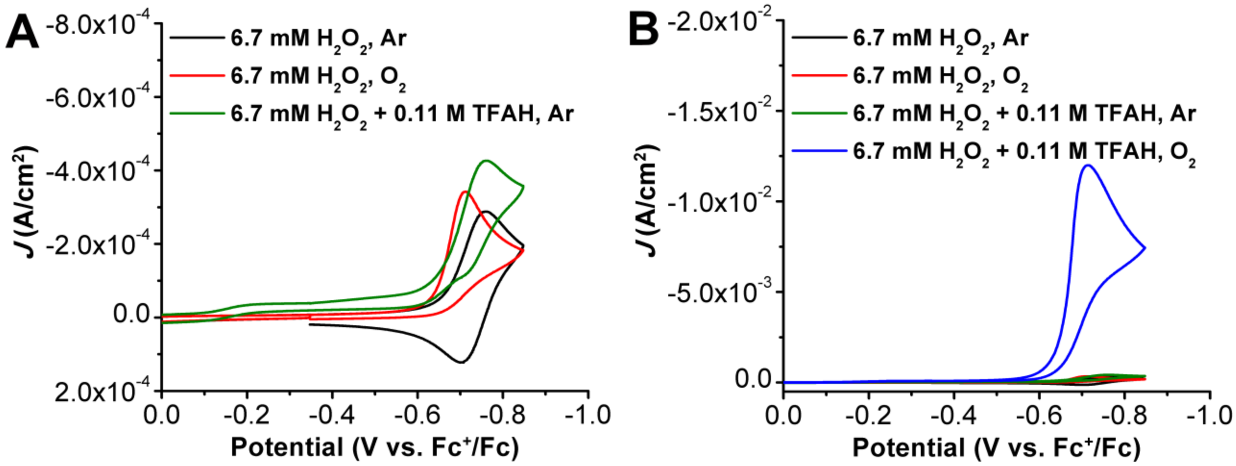

Figure S15. (A) CVs of **Ph_2Phen^{2+}** in the presence of 6.7 mM urea•H_2O_2 under Ar and O_2 saturation with added 0.11 M TFAH. (B) CVs of **Ph_2Phen^{2+}** in the presence of 6.7 mM urea•H_2O_2 with added 0.11 M TFAH under O_2 saturation. Conditions: 1 mM **Ph_2Phen^{2+}**, 0.11 M TFAH, 0.1 M TBAPF6/MeCN; 100 mV/s; glassy carbon working electrode, glassy carbon counter electrode, Ag/AgCl pseudoreference electrode; referenced to an internal ferrocene standard.

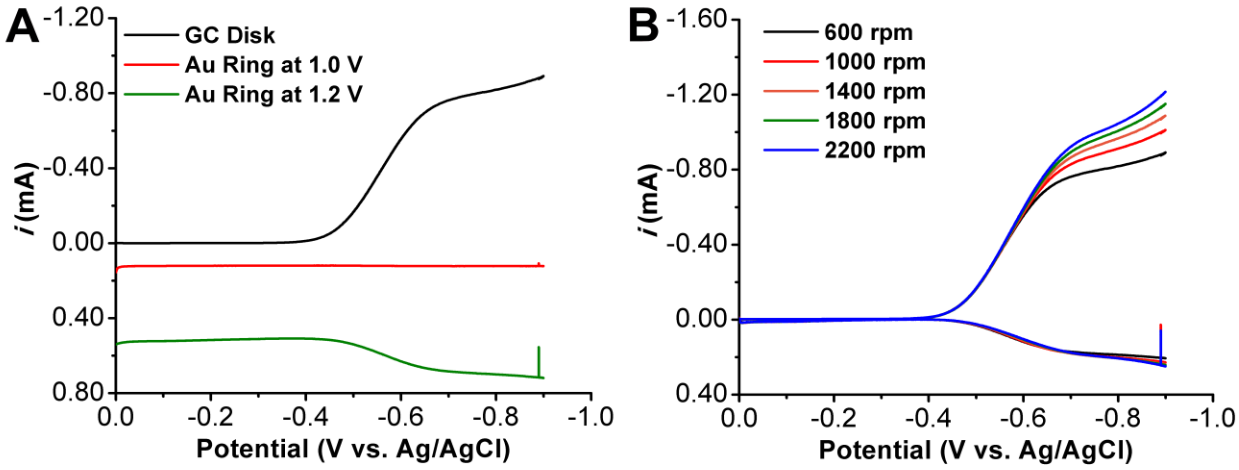

Figure S16. Linear sweep voltammograms of RRDE experiment with 0.5 mM **Ph_2Phen^{2+}** and 0.1 M TFAH under air saturation conditions. (A) Uncorrected LSVs at 600 rpm. (B) Corrected LSVs at various rotation rates used for quantification of %H_2O_2. Ring potential = 1.0 or 1.2 V vs Ag/AgCl. Conditions: 0.5 mM **Ph_2Phen^{2+}**, 0.1 M TFAH, 0.1 M $TBAPF_6$/MeCN; glassy carbon working electrode/roughened Au ring working electrode, glassy carbon counter electrode, Ag/AgCl pseudoreference electrode; scan rate 0.01 V/s.

Calculated ORR selectivity under electrochemical conditions using RRDE is 97.3 ± 2.6% H_2O_2.

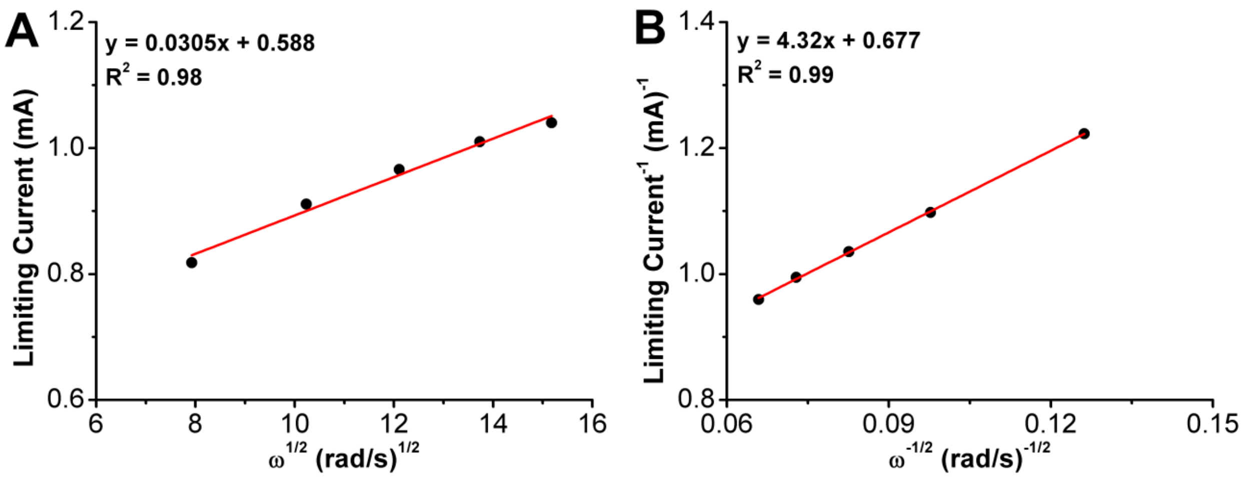

Figure S17. (A) Levich and (B) Koutecky-Levich plots from data obtained from linear sweep voltammograms of **Ph_2Phen^{2+}** (0.5 mM) by RRDE with 0.1 M TFAH under air saturation conditions at various rotation rates (600, 1000, 1400, 1800, & 2200 rpm).

Electrochemical Analysis with Cl_3AcOH

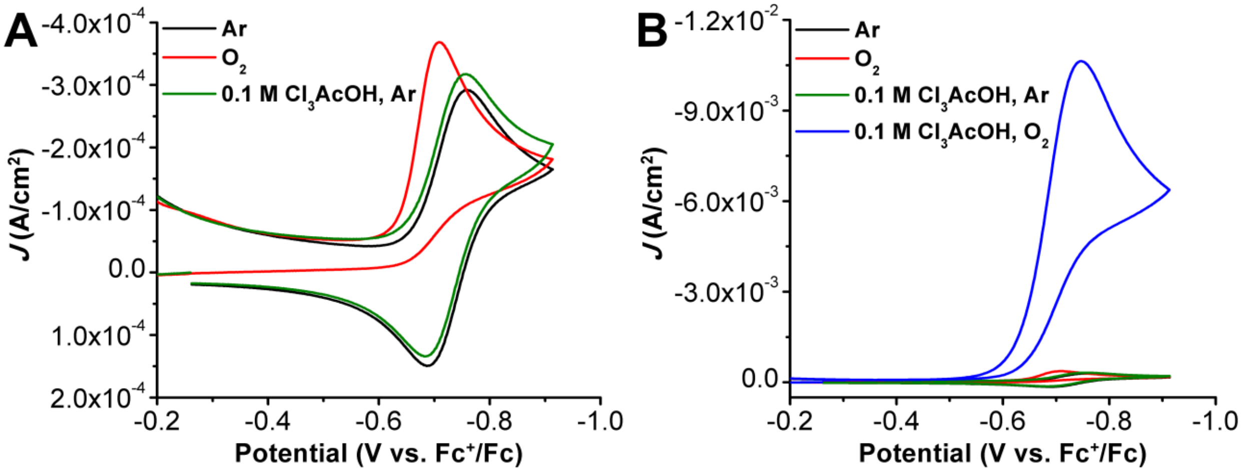

Figure S18. (A) CVs of **Ph_2Phen^{2+}** under Ar (black), O_2 (red) and with 0.1 M Cl_3AcOH under Ar saturation (green). (B) CVs from A and catalytic trace shown (blue) with **Ph_2Phen^{2+}** and 0.1 M Cl_3AcOH under O_2 saturation. Conditions: 1 mM **Ph_2Phen^{2+}**, 0.1 M $TBAPF_6$/MeCN; 100 mV/s; glassy carbon working electrode, glassy carbon rod counter electrode, Ag/AgCl pseudoreference electrode; referenced to an internal ferrocene standard.

*Determination of Effective Overpotential of **Ph_2Phen^{2+}** with Cl_3AcOH*

Utilizing the estimated pK_a of Cl_3AcOH (16.0) in MeCN, we can determine the effective overpotential according to **Eqs S10-S13**. Where E_{app} is the $E_{1/2}$ of **Ph_2Phen^{2+}** with 0.1 M Cl_3AcOH (**Figure S18**). We are unaware of a homoconjugation constant for further correction.

$$E^0_{O2/H2O} = 1.21 - 0.0592pK_a \qquad \textbf{Eq (S10)}$$

$$E^0_{O2/H2O}(MeCN, Cl_3AcOH) = \; 0.26\,V\; vs. Fc^+/Fc$$

$$\eta = \left|E_{app} - E^0_{O2/H2O}\right| = |-0.72 - 0.26| = 0.98\,V \qquad \textbf{Eq (S11)}$$

$$E^0_{O2/H2O2} = 0.695 - 0.0592pK_a$$

$$E^0_{O2/H2O2}(MeCN, Cl_3AcOH) = \; -0.25\,V\; vs. Fc^+/Fc \qquad \textbf{Eq (S12)}$$

$$\eta = \left|E_{app} - E^0_{O2/H2O2}\right| = |-0.72 - 0.25| = 0.47\,V \qquad \textbf{Eq (S13)}$$

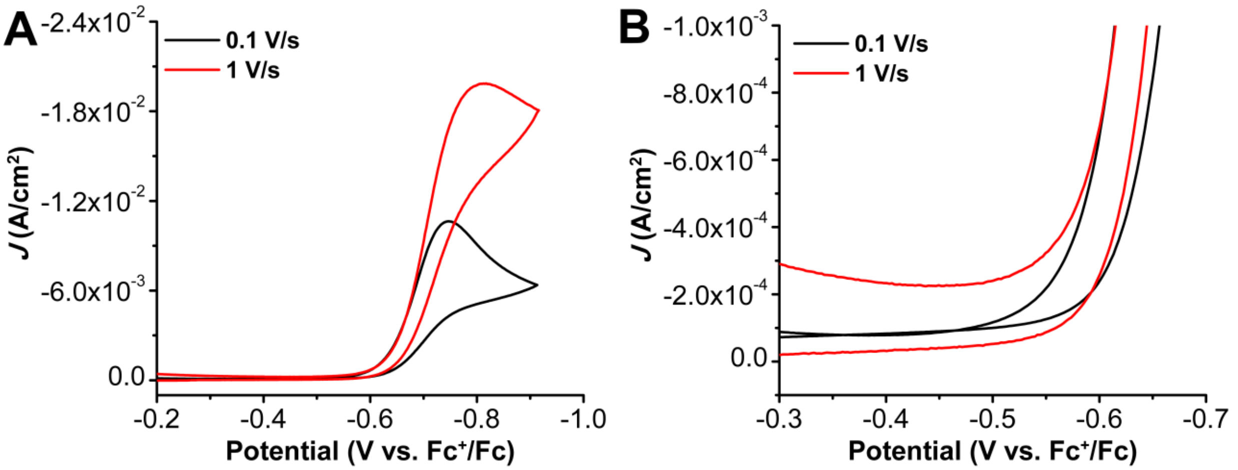

Figure S19. (A) CVs of **Ph_2Phen^{2+}** under catalytic conditions with 0.1 M Cl_3AcOH at 0.1 V/s (black) and 1 V/s (red) (B) CVs from A to emphasize observed cross-tracing. Conditions: 1 mM **Ph_2Phen^{2+}**, 0.1 M Cl_3AcOH, 0.1 M $TBAPF_6$/MeCN; 100 mV/s; glassy carbon working electrode, glassy carbon rod counter electrode, Ag/AgCl pseudoreference electrode; referenced to an internal ferrocene standard.

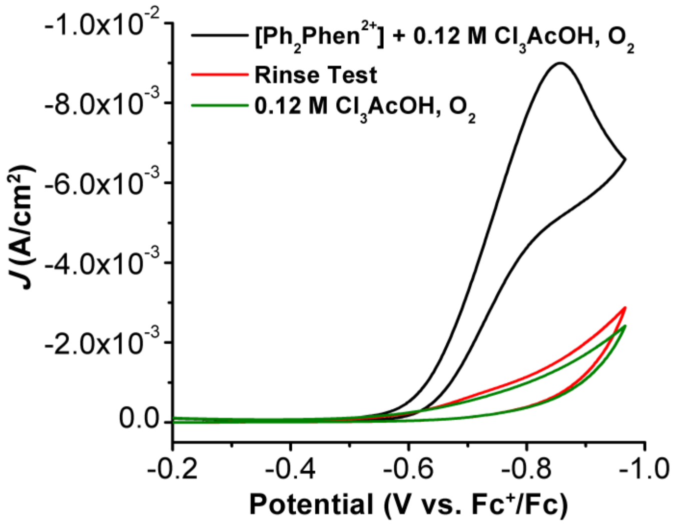

Figure S20. Rinse test of **Ph_2Phen^{2+}** and Cl_3AcOH. **Ph_2Phen^{2+}** under catalytic conditions (black trace), rinse test (red trace), and CV of Cl_3AcOH under O_2 saturation (green trace). Conditions: 1 mM **Ph_2Phen^{2+}**, 0.12 M Cl_3AcOH, 0.1 M $TBAPF_6$/MeCN, O_2 saturation; 100 mV/s; glassy carbon working electrode, glassy carbon counter electrode, Ag/AgCl pseudoreference electrode; referenced to an internal ferrocene standard.

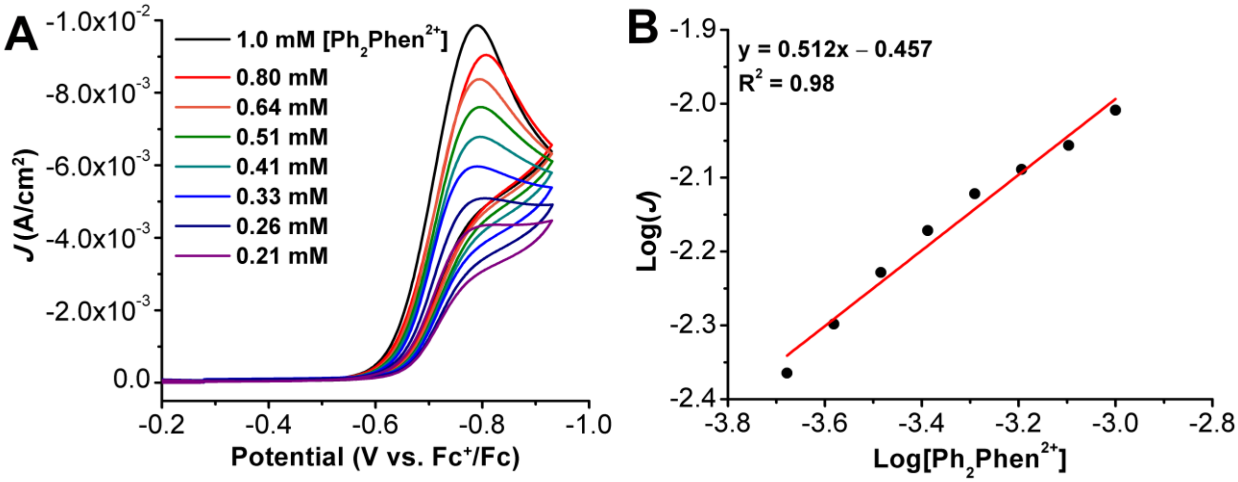

Figure S21. (A) CVs of **Ph_2Phen^{2+}** under catalytic conditions with variable **Ph_2Phen^{2+}** concentrations. (B) Logarithm of **Ph_2Phen^{2+}** concentration versus the logarithm of current density from (A). Conditions: 0.1 M Cl_3AcOH 0.1 M Cl_3AcOH, 0.1 M $TBAPF_6$/MeCN; 100 mV/s; glassy carbon working electrode, glassy carbon rod counter electrode, Ag/AgCl pseudoreference electrode; referenced to an internal ferrocene standard.

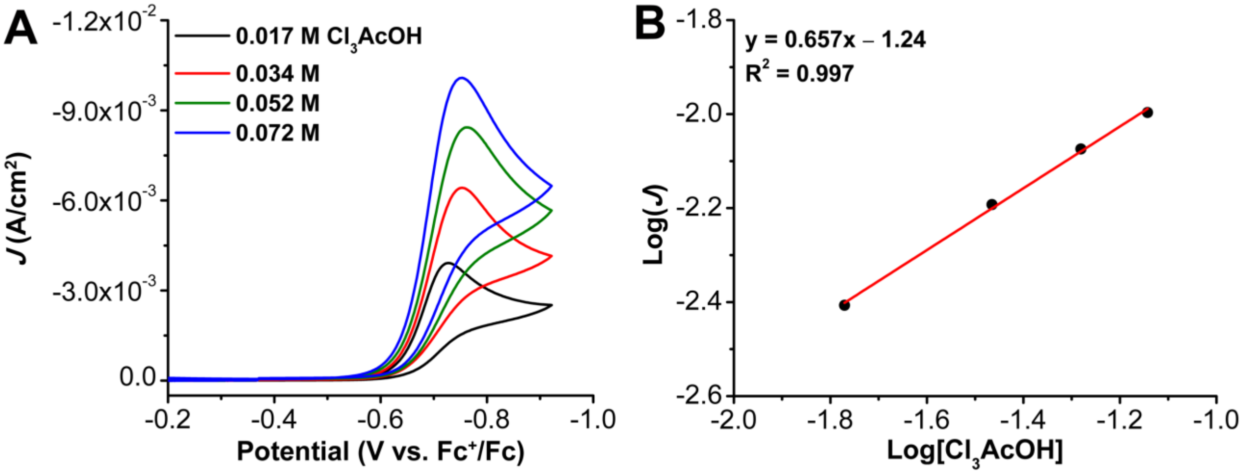

Figure S22. CVs of **Ph_2Phen^{2+}** under catalytic conditions with variable Cl_3AcOH concentrations. Conditions: 1 mM **Ph_2Phen^{2+}**, 0.1 M $TBAPF_6$/MeCN, O_2 saturation; 100 mV/s; glassy carbon working electrode, glassy carbon counter electrode, Ag/AgCl pseudoreference electrode; referenced to an internal ferrocene standard.

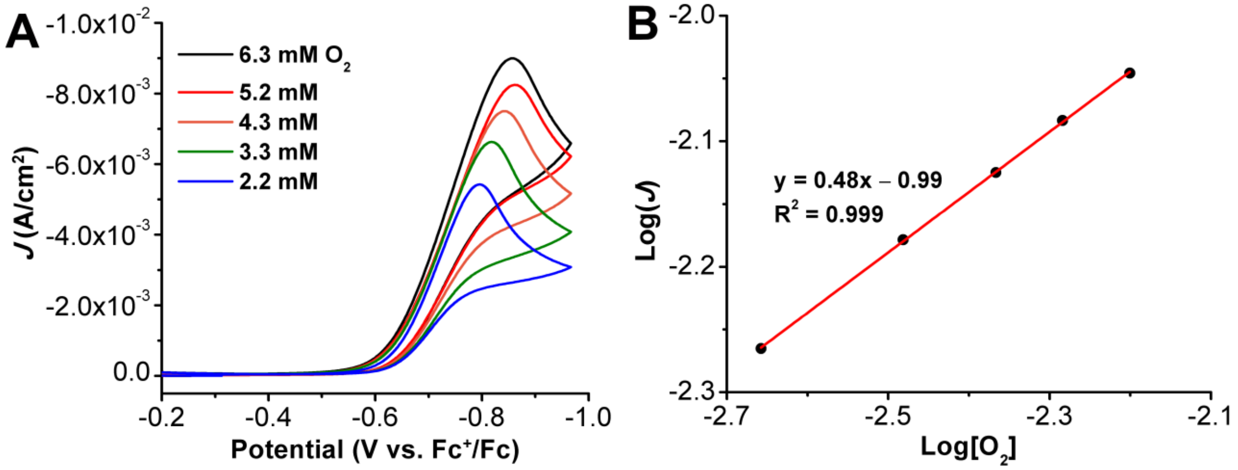

Figure S23. (A) CVs of **Ph_2Phen^{2+}** under catalytic conditions with variable O_2 concentrations. (B) Logarithm of O_2 concentration versus the logarithm of current density from (A). Conditions: 1 mM **Ph_2Phen^{2+}**, 0.12 M Cl_3AcOH, 0.1 M $TBAPF_6$/MeCN; 100 mV/s; glassy carbon working electrode, glassy carbon counter electrode, Ag/AgCl pseudoreference electrode; referenced to an internal ferrocene standard.

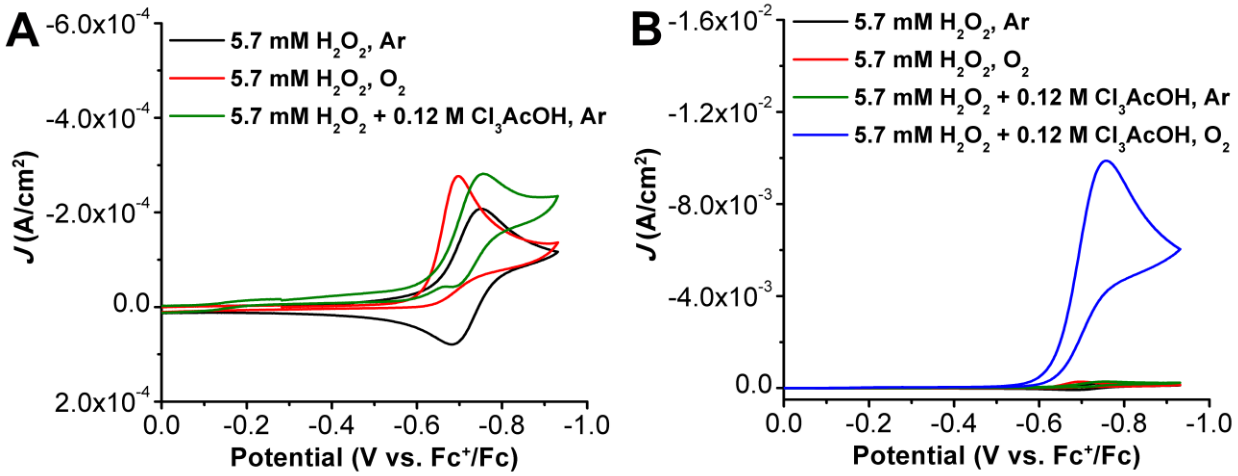

Figure S24. (A) CVs of **Ph_2Phen^{2+}** in the presence of 5.7 mM urea•H_2O_2 under Ar and O_2 saturation with added 0.12 M Cl_3AcOH. (B) CVs of **Ph_2Phen^{2+}** in the presence of 5.7 mM urea•H_2O_2 with added 0.12 M Cl_3AcOH under O_2 saturation. Conditions: 1 mM **Ph_2Phen^{2+}**, 0.12 M Cl_3AcOH, 0.1 M $TBAPF_6$/MeCN; 100 mV/s; glassy carbon working electrode, glassy carbon counter electrode, Ag/AgCl pseudoreference electrode; referenced to an external ferrocene standard.

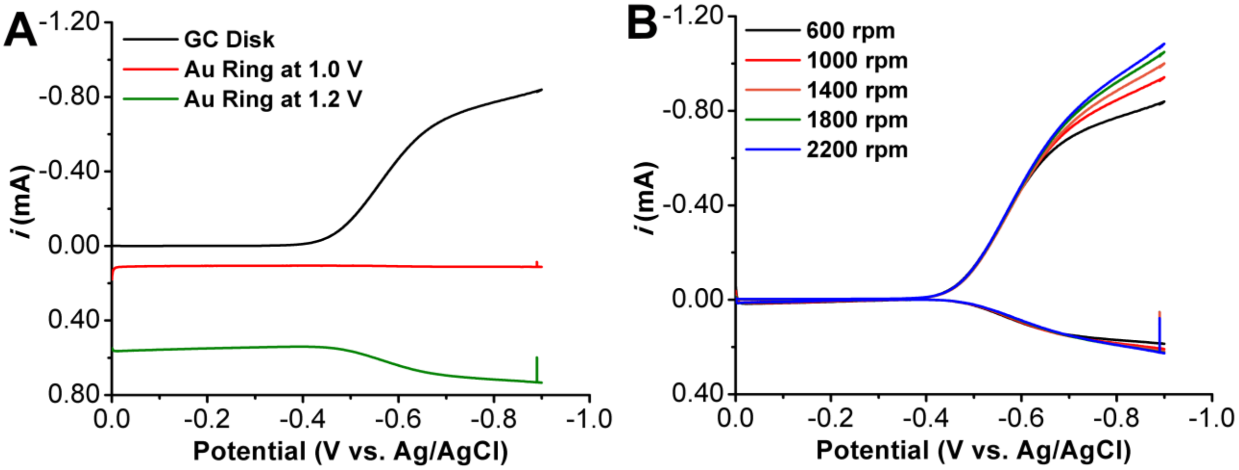

Figure S25. Linear sweep voltammograms of RRDE experiment with 0.5 mM **Ph_2Phen^{2+}** and 0.1 M Cl_3AcOH under air saturation conditions. (A) Uncorrected LSVs at 600 rpm. (B) Corrected LSVs at various rotation rates used for quantification of %H_2O_2. Ring potential = 1.0 or 1.2 V vs Ag/AgCl. Conditions: 0.5 mM **Ph_2Phen^{2+}**, 0.1 M Cl_3AcOH, 0.1 M $TBAPF_6$/MeCN; glassy carbon working electrode/roughened Au ring working electrode, glassy carbon counter electrode, Ag/AgCl pseudoreference electrode; scan rate 0.01 V/s.

Calculated ORR selectivity under electrochemical conditions using RRDE is 96.9 ± 0.85% H_2O_2.

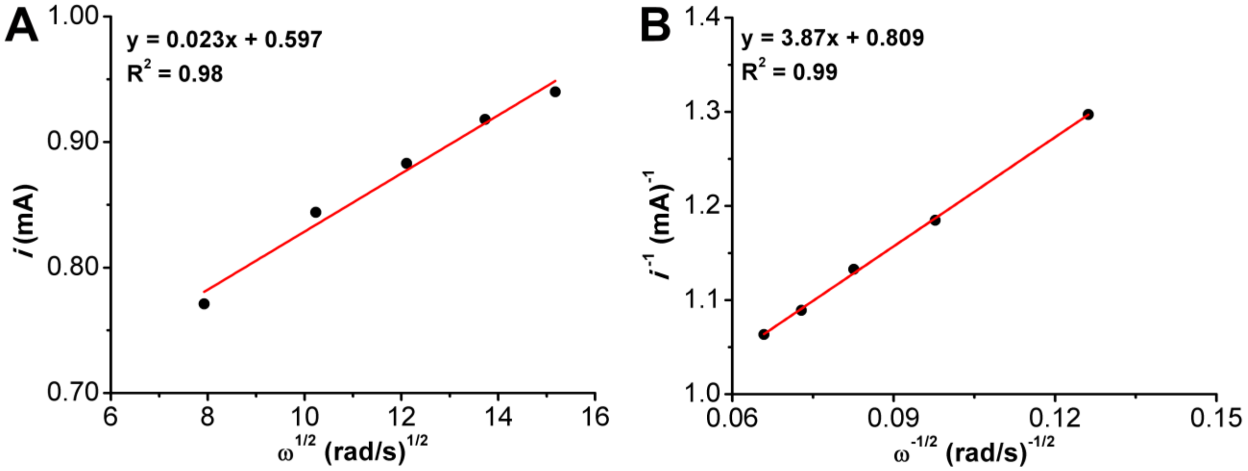

Figure S26. (A) Levich and (B) Koutecky-Levich plots from data obtained from linear sweep voltammograms of **Ph_2Phen^{2+}** (0.5 mM) by RRDE with 0.1 M Cl_3AcOH under air saturation conditions at various rotation rates (600, 1000, 1400, 1800, & 2200 rpm).

Electrochemical Analysis with Cl_2AcOH

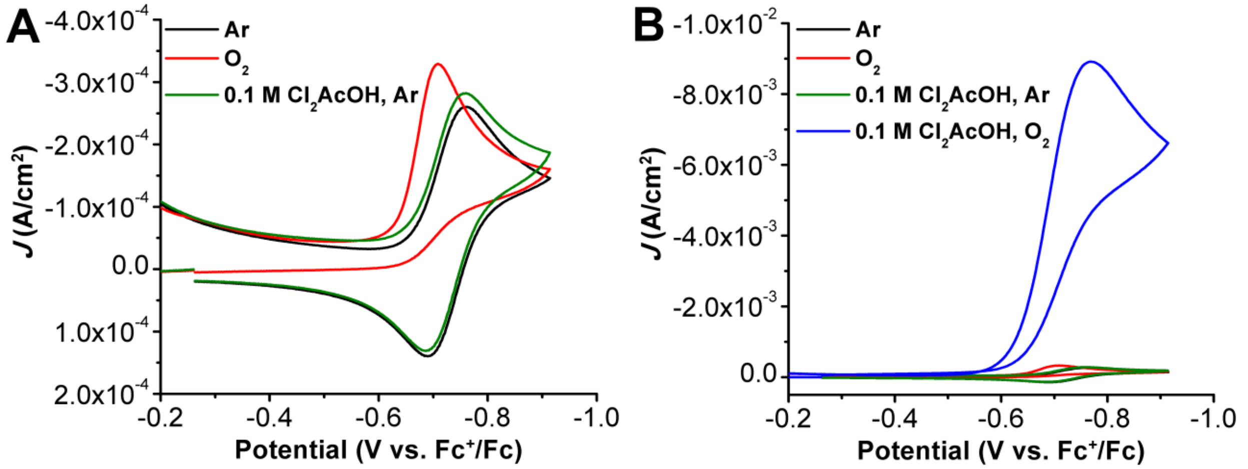

Figure S27. (A) CVs of **Ph_2Phen^{2+}** under Ar (black), O_2 (red) and with 0.1 M Cl_2AcOH under Ar saturation (green). (B) CVs from A and catalytic trace shown (blue) with Ph_2Phen^{2+} and 0.1 M Cl_2AcOH under O_2 saturation. Conditions: 1 mM **Ph_2Phen^{2+}**, 0.1 M $TBAPF_6$/MeCN; 100 mV/s; glassy carbon working electrode, glassy carbon rod counter electrode, Ag/AgCl pseudoreference electrode; referenced to an internal ferrocene standard.

Determination of Effective Overpotential of Ph_2Phen^{2+} with Cl_2AcOH

Utilizing the estimated pK_a of Cl_2AcOH (17.3) in MeCN, we can determine the effective overpotential according to **Eqs S13-S16**. Where E_{app} is the $E_{1/2}$ of Ph_2Phen^{2+} with 0.1 M Cl_2AcOH (**Figure S27**). We are unaware of a homoconjugation constant for further correction.

$$E^0_{O2/H2O} = 1.21 - 0.0592pK_a \qquad \textbf{Eq (S13)}$$

$$E^0_{O2/H2O}(MeCN, Cl_2AcOH) = 0.19\ V\ vs. Fc^+/Fc$$

$$\eta = \left|E_{app} - E^0_{O2/H2O}\right| = |-0.72 - 0.19| = 0.91\ V \qquad \textbf{Eq (S14)}$$

$$E^0_{O2/H2O2} = 0.695 - 0.0592pK_a$$

$$E^0_{O2/H2O2}(MeCN, Cl_2AcOH) = -0.33\ V\ vs. Fc^+/Fc \qquad \textbf{Eq (S15)}$$

$$\eta = \left|E_{app} - E^0_{O2/H2O2}\right| = |-0.72 - 0.33| = 0.39\ V \qquad \textbf{Eq (S16)}$$

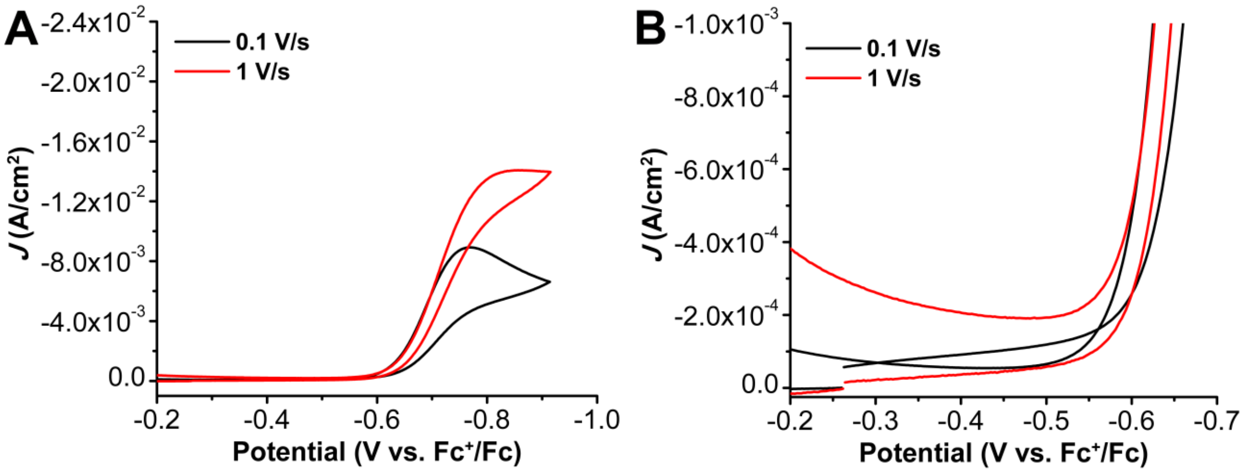

Figure S28. (A) CVs of $\mathbf{Ph_2Phen^{2+}}$ under catalytic conditions with 0.1 M Cl_2AcOH at 0.1 V/s (black) and 1 V/s (red) (B) CVs from A to emphasize observed cross-tracing. Conditions: 1 mM $\mathbf{Ph_2Phen^{2+}}$, 0.1 M Cl_2AcOH, 0.1 M $TBAPF_6$/MeCN; glassy carbon working electrode, glassy carbon rod counter electrode, Ag/AgCl pseudoreference electrode; referenced to an internal ferrocene standard.

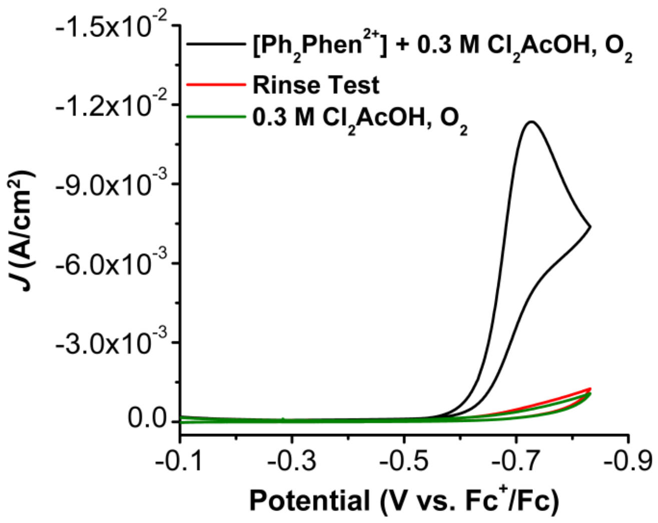

Figure S29. Rinse test of **Ph_2Phen^{2+}** and Cl_2AcOH. **Ph_2Phen^{2+}** under catalytic conditions (black trace), rinse test (red trace), and CV of Cl_2AcOH under O_2 saturation (green trace). Conditions: 1 mM **Ph_2Phen^{2+}**, 0.3 M Cl_2AcOH, 0.1 M $TBAPF_6$/MeCN, O_2 saturation; 100 mV/s; glassy carbon working electrode, glassy carbon counter electrode, Ag/AgCl pseudoreference electrode; referenced to an internal ferrocene standard.

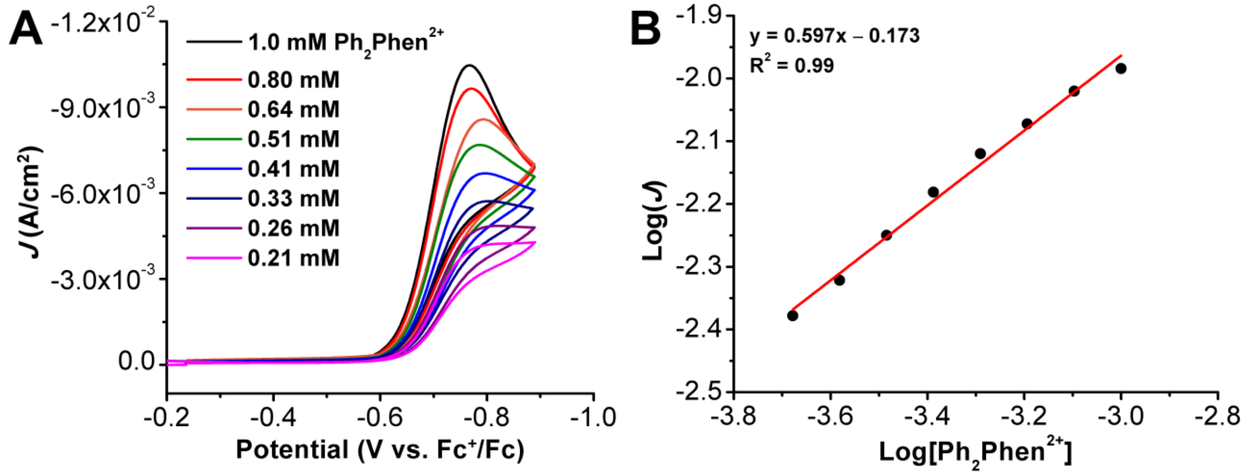

Figure S30. CVs of **Ph_2Phen^{2+}** under catalytic conditions with variable **Ph_2Phen^{2+}** concentrations. (B) Logarithm of **Ph_2Phen^{2+}** concentration versus the logarithm of current density from (A). Conditions: 0.3 M Cl_2AcOH, 0.1 M $TBAPF_6$/MeCN; 100 mV/s; glassy carbon working electrode, glassy carbon counter electrode, Ag/AgCl pseudoreference electrode; referenced to an internal ferrocene standard.

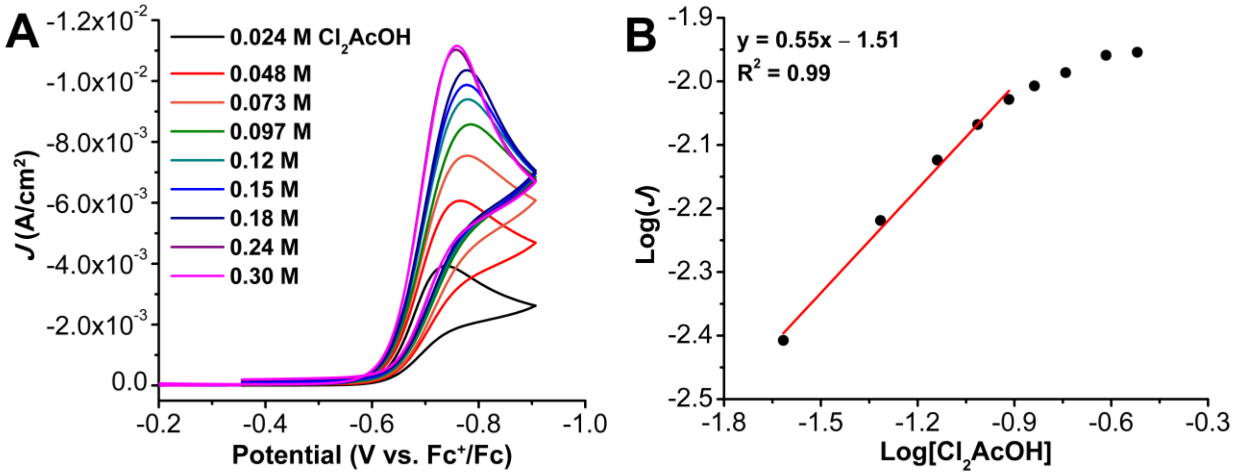

Figure S31. (A) CVs of **Ph_2Phen^{2+}** under catalytic conditions with variable Cl_2AcOH concentrations. (B) Logarithm of Cl_2AcOH concentration versus the logarithm of current density from (A). Conditions: 1 mM **Ph_2Phen^{2+}**, 0.1 M $TBAPF_6$/MeCN, O_2 saturation; 100 mV/s; glassy carbon working electrode, glassy carbon counter electrode, Ag/AgCl pseudoreference electrode; referenced to an internal ferrocene standard.

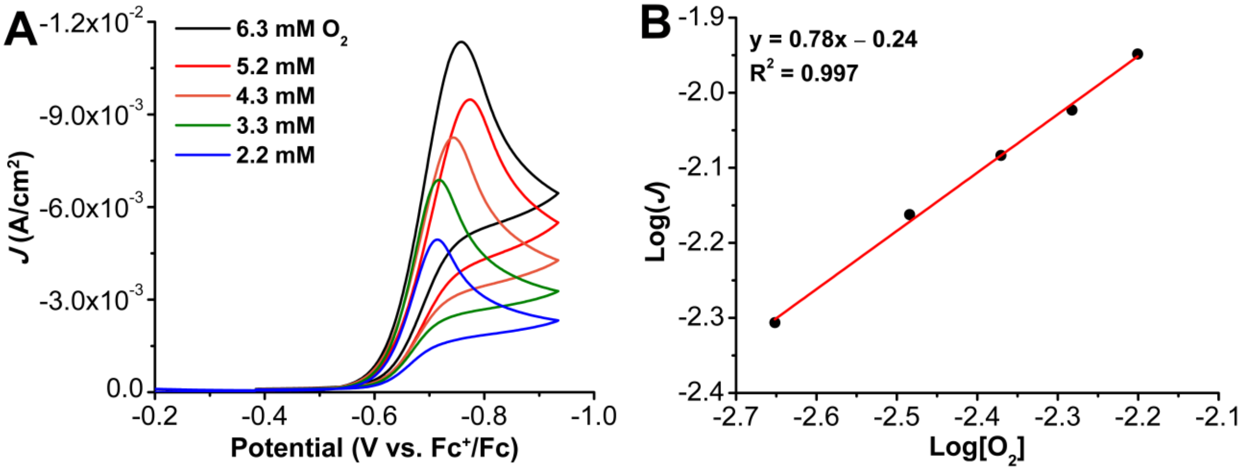

Figure S32. (A) CVs of **Ph_2Phen^{2+}** under catalytic conditions with variable O_2 concentrations. (B) Logarithm of O_2 concentration versus the logarithm of current density from (A). Conditions: 1 mM **Ph_2Phen^{2+}**, 0.3 M Cl_2AcOH, 0.1 M $TBAPF_6$/MeCN; 100 mV/s; glassy carbon working electrode, glassy carbon counter electrode, Ag/AgCl pseudoreference electrode; referenced to an internal ferrocene standard.

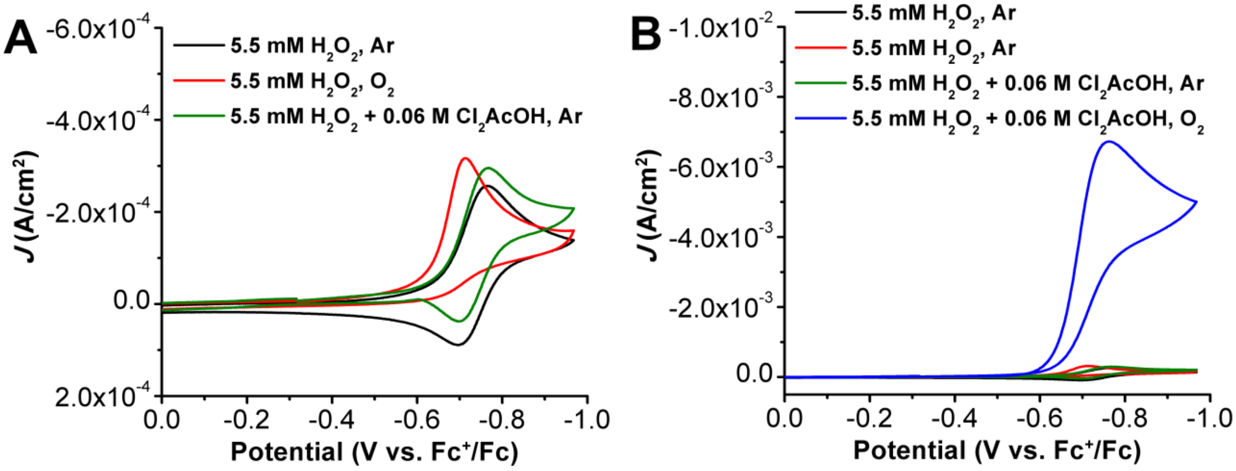

Figure S33. (A) CVs of **Ph_2Phen^{2+}** in the presence of 5.5 mM urea•H_2O_2 under Ar and O_2 saturation with added 0.06 M Cl_2AcOH. (B) CVs of **Ph_2Phen^{2+}** in the presence of 5.7 mM urea•H_2O_2 with added 0.06 M Cl_2AcOH under O_2 saturation. Conditions: 1 mM **Ph_2Phen^{2+}**, 0.06 M Cl_2AcOH, 0.1 M $TBAPF_6$/MeCN; 100 mV/s; glassy carbon working electrode, glassy carbon counter electrode, Ag/AgCl pseudoreference electrode; referenced to an external ferrocene standard.

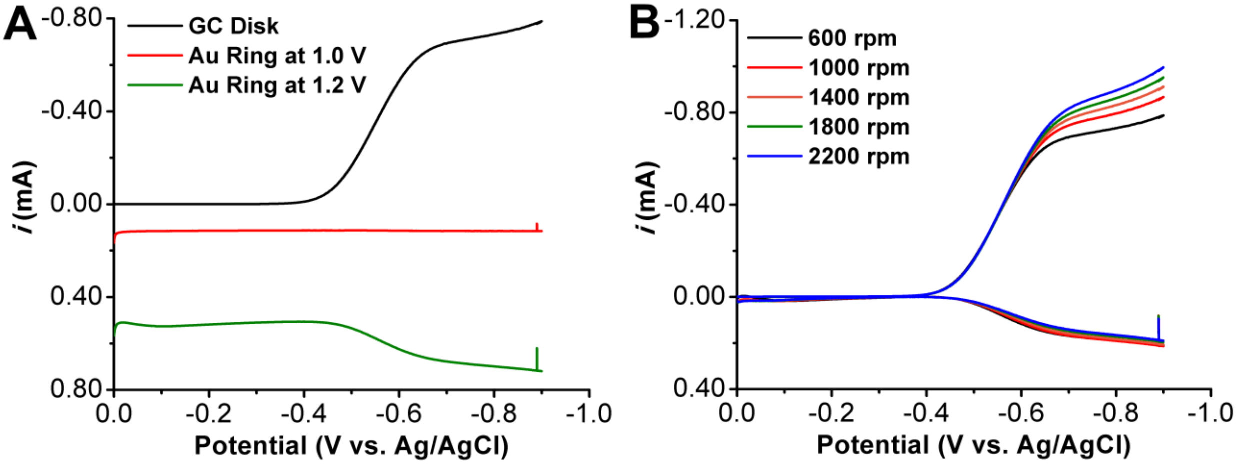

Figure S34. Linear sweep voltammograms of RRDE experiment with 0.5 mM **Ph_2Phen^{2+}** and 0.1 M Cl_2AcOH under air saturation conditions. (A) Uncorrected LSVs at 600 rpm. (B) Corrected LSVs at various rotation rates used for quantification of %H_2O_2. Ring potential = 1.0 or 1.2 V vs Ag/AgCl. Conditions: 0.5 mM **Ph_2Phen^{2+}**, 0.1 M Cl_2AcOH, 0.1 M $TBAPF_6$/MeCN; glassy carbon working electrode/roughened Au ring working electrode, glassy carbon counter electrode, Ag/AgCl pseudoreference electrode; scan rate 0.01 V/s.

Calculated ORR selectivity under electrochemical conditions using RRDE is 98.2 ± 7.8% H_2O_2.

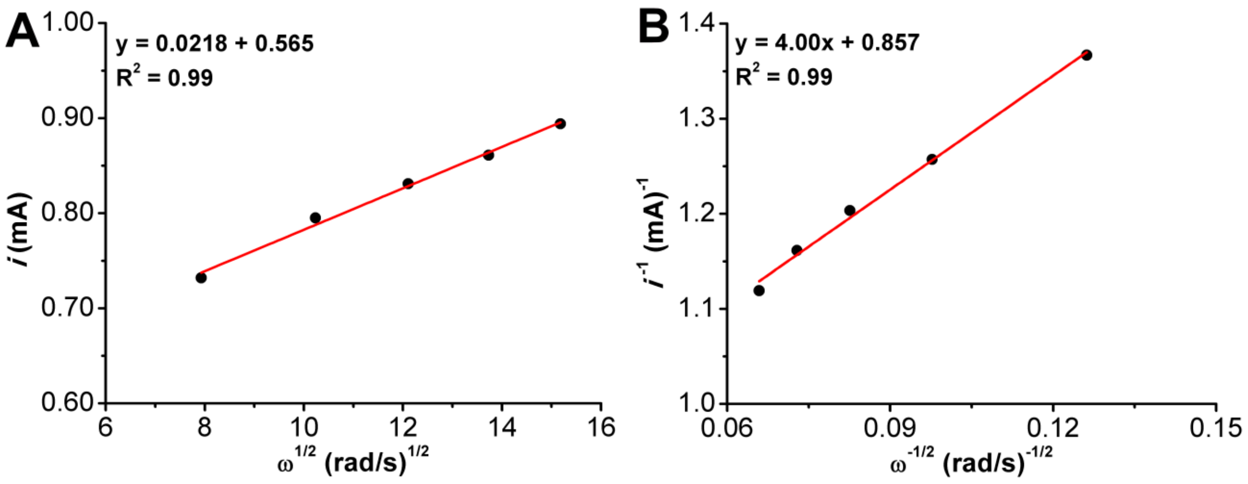

Figure S35. (A) Levich and (B) Koutecky-Levich plots from data obtained from linear sweep voltammograms of **Ph_2Phen^{2+}** (0.5 mM) by RRDE with 0.1 M Cl_2AcOH under air saturation conditions at various rotation rates (600, 1000, 1400, 1800, & 2200 rpm).

Electrochemical Analysis with ClAcOH

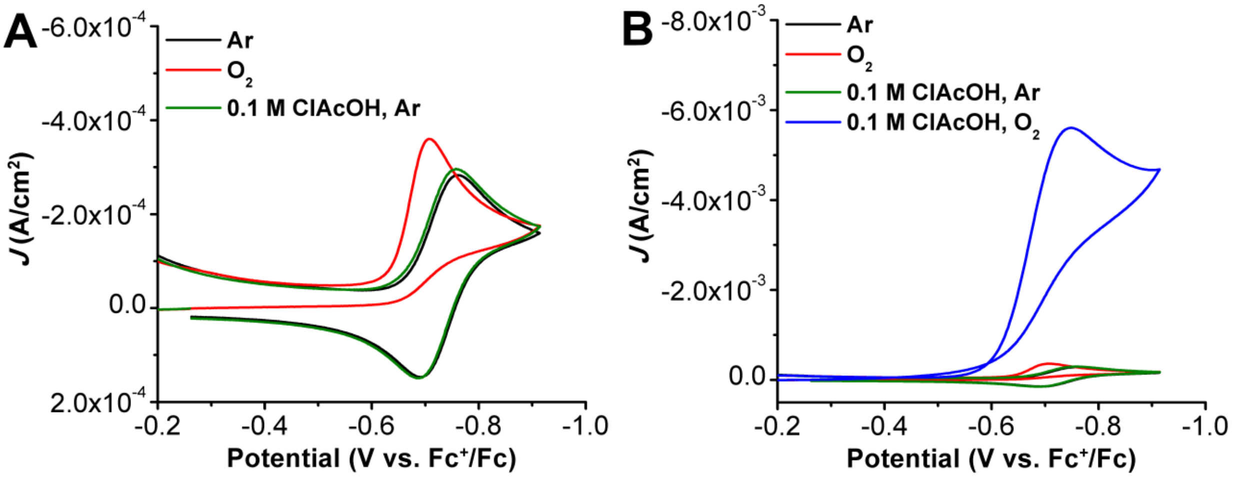

Figure S36. (A) CVs of **Ph_2Phen^{2+}** under Ar (black), O_2 (red) and with 0.1 M ClAcOH under Ar saturation (green). (B) CVs from A and catalytic trace shown (blue) with **Ph_2Phen^{2+}** and 0.1 M ClAcOH under O_2 saturation. Conditions: 1 mM **Ph_2Phen^{2+}**, 0.1 M $TBAPF_6$/MeCN; 100 mV/s; glassy carbon working electrode, glassy carbon rod counter electrode, Ag/AgCl pseudoreference electrode; referenced to an internal ferrocene standard.

Determination of Effective Overpotential of ***Ph₂Phen²⁺*** *with ClAcOH*

Utilizing the estimated pK_a of ClAcOH (20.25) in MeCN, we can determine the effective overpotential according to **Eqs S17-S20**. Where E_{app} is the $E_{1/2}$ of Ph_2Phen^{2+} with 0.1 M ClAcOH (**Figure S36)**. We are unaware of a homoconjugation constant for further correction.

$$E^0_{O2/H2O} = 1.21 - 0.0592pK_a \quad \textbf{Eq (S17)}$$

$$E^0_{O2/H2O}(MeCN, ClAcOH) = 0.01\,V\,vs. Fc^+/Fc$$

$$\eta = |E_{app} - E^0_{O2/H2O}| = |-0.72 - 0.01| = 0.73\,V \quad \textbf{Eq (S18)}$$

$$E^0_{O2/H2O2} = 0.695 - 0.0592pK_a$$

$$E^0_{O2/H2O2}(MeCN, ClAcOH) = -0.50\,V\,vs. Fc^+/Fc \quad \textbf{Eq (S19)}$$

$$\eta = |E_{app} - E^0_{O2/H2O2}| = |-0.72 - 0.50| = 0.22\,V \quad \textbf{Eq (S20)}$$

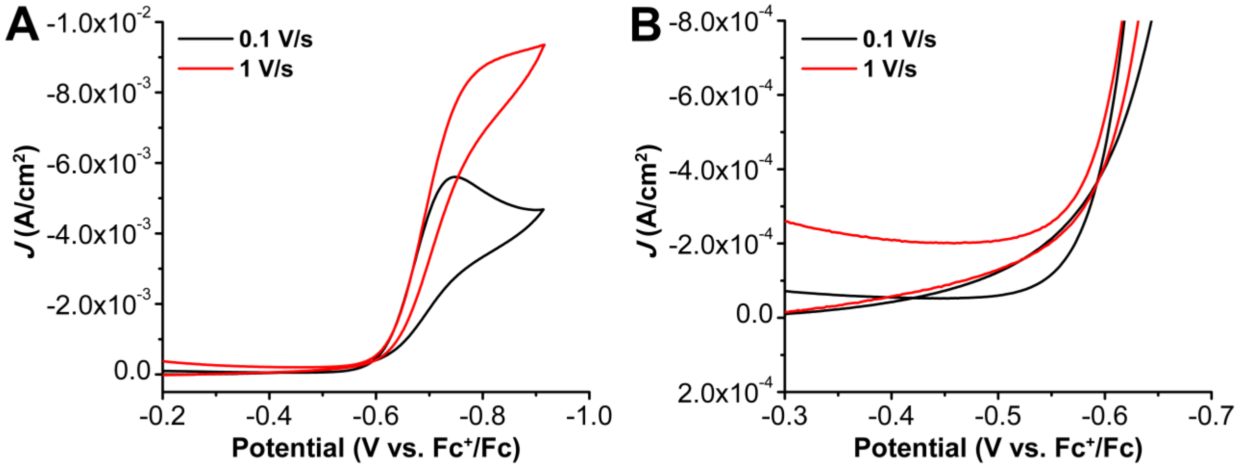

Figure S37. (A) CVs of $\mathbf{Ph_2Phen^{2+}}$ under catalytic conditions with 0.1 M ClAcOH at 0.1 V/s (black) and 1 V/s (red) (B) CVs from A to emphasize observed cross-tracing. Conditions: 1 mM $\mathbf{Ph_2Phen^{2+}}$, 0.1 M ClAcOH, 0.1 M $TBAPF_6$/MeCN; glassy carbon working electrode, glassy carbon rod counter electrode, Ag/AgCl pseudoreference electrode; referenced to an internal ferrocene standard.

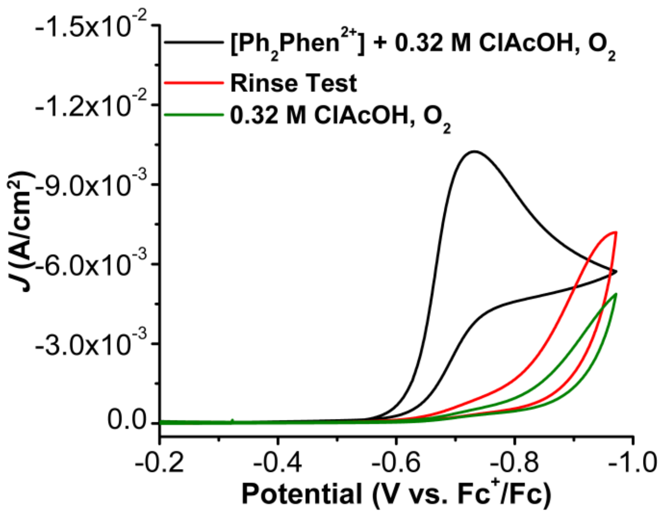

Figure S38. Rinse test of **Ph_2Phen^{2+}** and ClAcOH. **Ph_2Phen^{2+}** under catalytic conditions (black trace), rinse test (red trace), and CV of Cl_2AcOH under O_2 saturation (green trace). Conditions: 1 mM **Ph_2Phen^{2+}**, 0.32 M ClAcOH, 0.1 M $TBAPF_6$/MeCN, O_2 saturation; 100 mV/s; glassy carbon working electrode, glassy carbon counter electrode, Ag/AgCl pseudoreference electrode; referenced to an internal ferrocene standard.

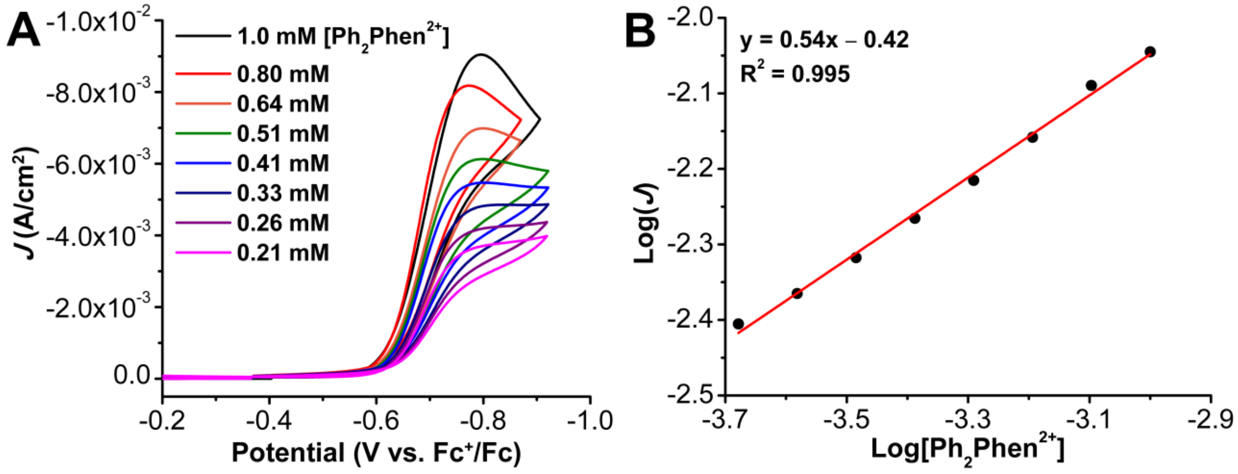

Figure S39. CVs of **Ph_2Phen^{2+}** under catalytic conditions with variable **Ph_2Phen^{2+}** concentrations. (B) Logarithm of **Ph_2Phen^{2+}** concentration versus the logarithm of current density from (A). Conditions: 0.3 M ClAcOH, 0.1 M $TBAPF_6$/MeCN; 100 mV/s; glassy carbon working electrode, glassy carbon counter electrode, Ag/AgCl pseudoreference electrode; referenced to an internal ferrocene standard.

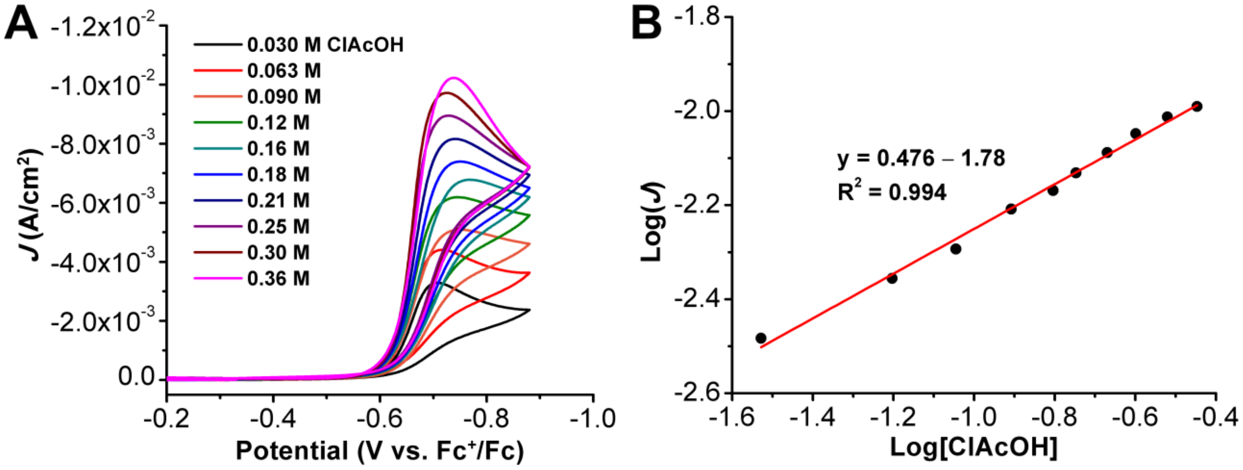

Figure S40. (A) CVs of **Ph_2Phen^{2+}** under catalytic conditions with variable ClAcOH concentrations. (B) Logarithm of ClAcOH concentration versus the logarithm of current density from (A). Conditions: 1 mM **Ph_2Phen^{2+}**, 0.1 M $TBAPF_6$/MeCN; 100 mV/s; glassy carbon working electrode, glassy carbon counter electrode, Ag/AgCl pseudoreference electrode; referenced to an internal ferrocene standard.

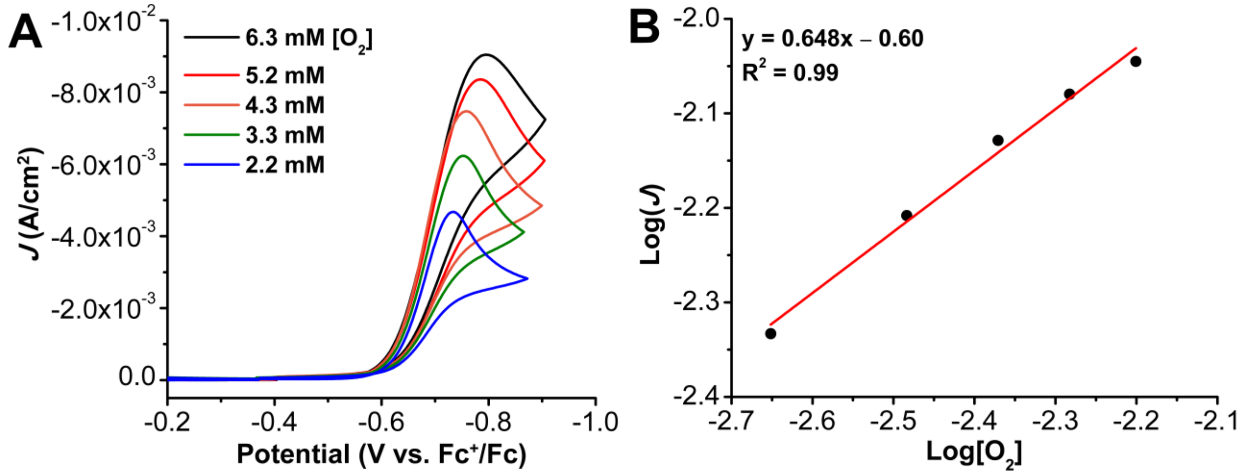

Figure S41. (A) CVs of **Ph_2Phen^{2+}** under catalytic conditions with variable O_2 concentrations. (B) Logarithm of O_2 concentration versus the logarithm of current density from (A). Conditions: 1 mM **Ph_2Phen^{2+}**, 0.3 M ClAcOH, 0.1 M $TBAPF_6$/MeCN; 100 mV/s; glassy carbon working electrode, glassy carbon counter electrode, Ag/AgCl pseudoreference electrode; referenced to an internal ferrocene standard.

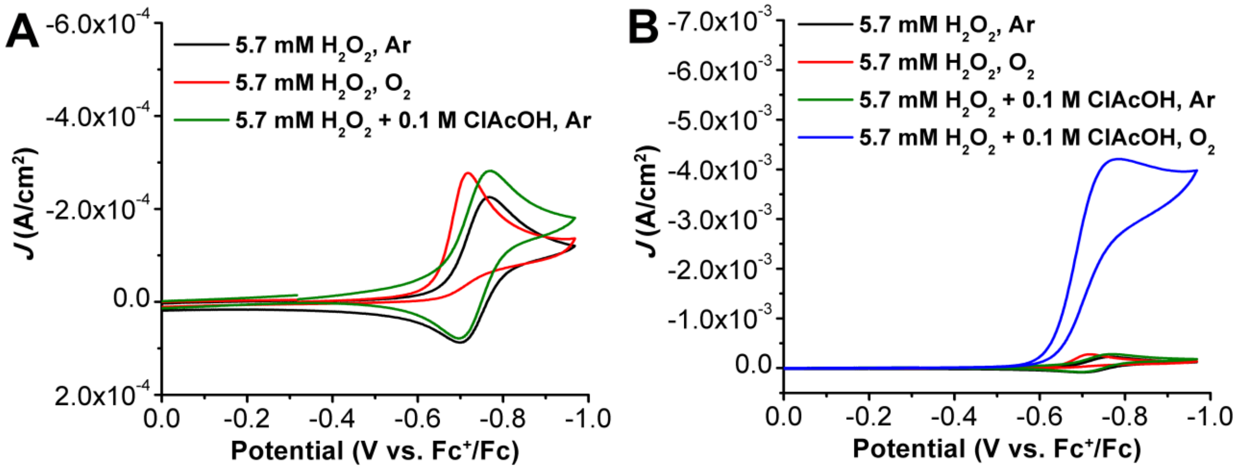

Figure S42. (A) CVs of **Ph_2Phen^{2+}** in the presence of 5.7 mM urea•H_2O_2 under Ar and O_2 saturation with added 0.1 M ClAcOH. (B) CVs of **Ph_2Phen^{2+}** in the presence of 5.7 mM urea•H_2O_2 with added 0.1 M ClAcOH under O_2 saturation. Conditions: 1 mM **Ph_2Phen^{2+}**, 0.1 M ClAcOH, 0.1 M $TBAPF_6$/MeCN; 100 mV/s; glassy carbon working electrode, glassy carbon counter electrode, Ag/AgCl pseudoreference electrode; referenced to an external ferrocene standard

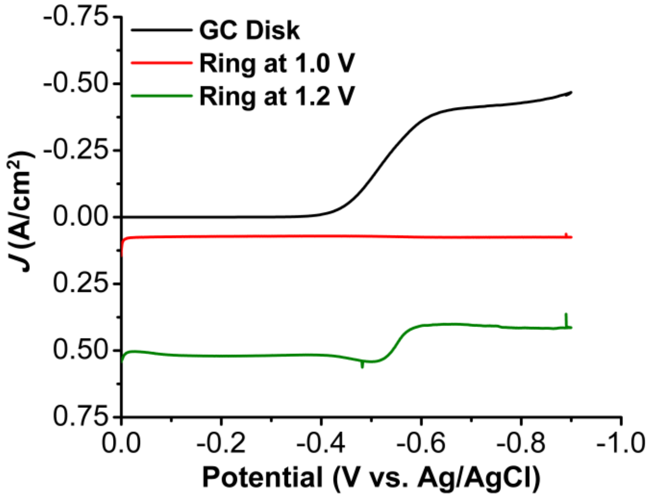

Figure S43. Linear sweep voltammogram of RRDE experiment with 0.5 mM **Ph_2Phen^{2+}** and 0.1 M ClAcOH under air saturation conditions at 200 rpm. Ring potential = 1.0 or 1.2 V vs Ag/AgCl. Conditions: 0.5 mM **Ph_2Phen^{2+}**, 0.1 M Cl_2AcOH, 0.1 M $TBAPF_6$/MeCN; glassy carbon working electrode/roughened Au ring working electrode, glassy carbon counter electrode, Ag/AgCl pseudoreference electrode; scan rate 0.01 V/s.

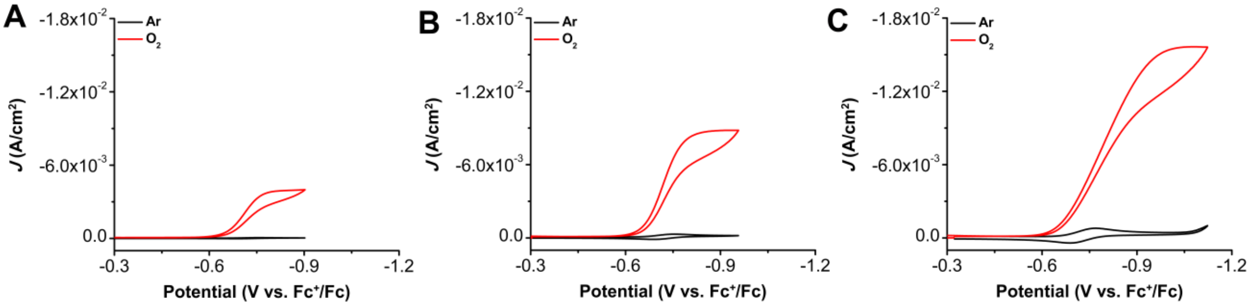

Figure S44. CVs under catalytical conditions with variable **Ph_2Phen^{2+}** concentrations and scan rates in order to achieve an S-shaped catalytic wave with TFAH as a proton source. (A) 0.21 mM **Ph_2Phen^{2+}** and 75 mV/s, (B) 0.41 mM **Ph_2Phen^{2+}** and 500 mV/s, and (C) 0.82 mM **Ph_2Phen^{2+}** 1000 mV/s. Conditions: 0.1 M TFAH, 0.1 M $TBAPF_6$/MeCN; glassy carbon working electrode, glassy carbon counter electrode, Ag/AgCl pseudoreference electrode; referenced to an internal ferrocene standard.

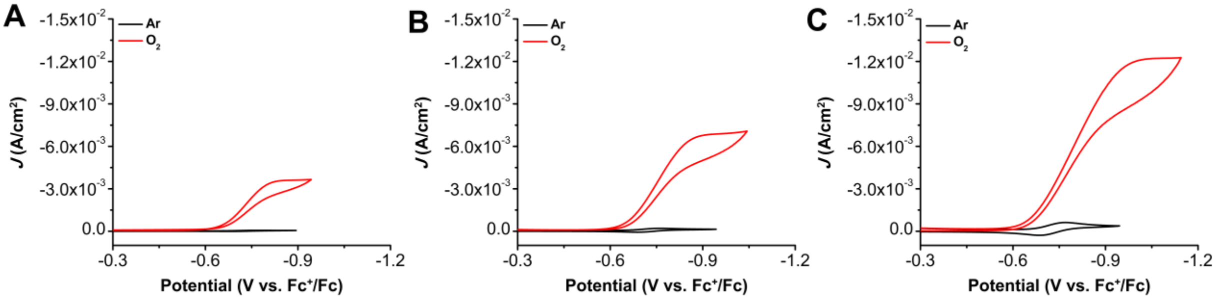

Figure S45. CVs under catalytic conditions with variable **Ph_2Phen^{2+}** concentrations and scan rates in order to achieve an S-shaped catalytic wave with Cl_3AcOH as a proton source. (A) 0.20 mM **Ph_2Phen^{2+}** and 50 mV/s, (B) 0.39 mM **Ph_2Phen^{2+}** and 200 mV/s, and (C) 0.79 mM **Ph_2phen^{2+}** 600 mV/s. Conditions: 0.1 M Cl_3AcOH, 0.1 M $TBAPF_6$/MeCN; glassy carbon working electrode, glassy carbon counter electrode, Ag/AgCl pseudoreference electrode; referenced to an internal ferrocene standard.

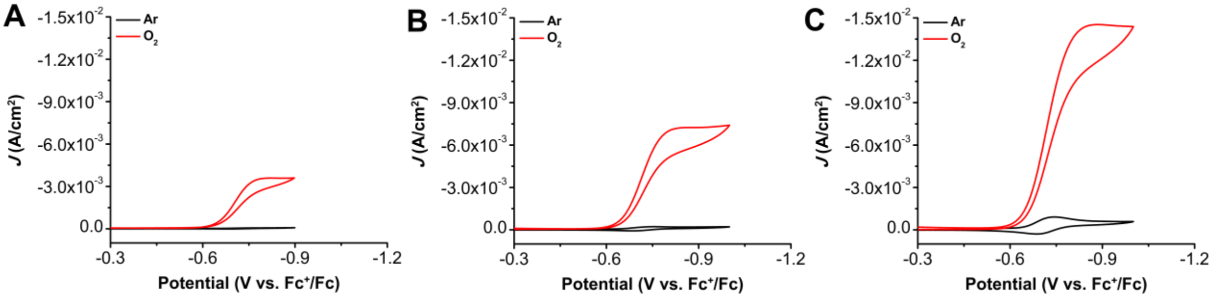

Figure S46. CVs under catalytic conditions with variable **Ph_2Phen^{2+}** concentrations and scan rates in order to achieve an S-shaped catalytic wave with Cl_2AcOH as a proton source. (A) 0.21 mM **Ph_2Phen^{2+}** and 75 mV/s, (B) 0.41 mM **Ph_2Phen^{2+}** and 300 mV/s, and (C) 0.82 mM **Ph_2Phen^{2+}** 1000 mV/s. Conditions: 0.3 M Cl_2AcOH, 0.1 M $TBAPF_6$/MeCN; glassy carbon working electrode, glassy carbon counter electrode, Ag/AgCl pseudoreference electrode; referenced to an internal ferrocene standard.

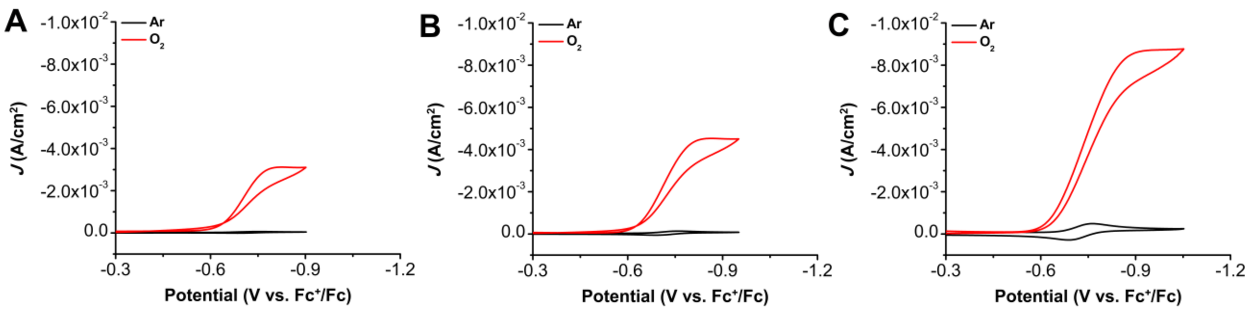

Figure S47. CVs under catalytical conditions with variable **Ph_2Phen^{2+}** concentrations and scan rates in order to achieve an S-shaped catalytic wave with ClAcOH as a proton source. (A) 0.20 mM **Ph_2Phen^{2+}** and 50 mV/s, (B) 0.40 mM **Ph_2Phen^{2+}** and 100 mV/s, and (C) 0.80 mM **Ph_2Phen^{2+}** 400 mV/s. Conditions: 0.3 M ClAcOH, 0.1 M $TBAPF_6$/MeCN; glassy carbon working electrode, glassy carbon counter electrode, Ag/AgCl pseudoreference electrode; referenced to an internal ferrocene standard.

Table S2. Calculated i_{cat}/i_p and TOF values for variable **Ph_2Phen^{2+}** concentration at all catalytic conditions.

Acid	[**Ph_2Phen^{2+}**] (mM)	Scan Rate (mV/s)	i_{cat}/i_p	TOF (s^{-1})
TFAH	0.21	75	109.2	1.73E+03
	0.41	500	38.2	1.41E+03
	0.82	1000	22.9	1.01E+03
Cl_3AcOH	0.20	50	96.2	8.97E+02
	0.39	200	48.2	8.99E+02
	0.79	600	24.5	6.98E+02
Cl_2AcOH	0.21	75	100.8	1.48E+03
	0.41	300	43.0	1.08E+03
	0.82	1000	18.1	6.34E+02
ClAcOH	0.20	50	77.2	5.78E+02
	0.40	100	44.8	3.89E+02
	0.80	400	20.9	3.40E+02

$$\mathrm{TOF} = \frac{Fvn_p^3}{RT}\left(\frac{0.4463}{n_{cat}}\right)^2\left(\frac{i_{cat}}{i_p}\right)^2$$

Where is F Faraday's constant, v is scan rate (V/s), T is the ideal gas constant, T the temperature (K), n_p number of electrons corresponding to the Faradaic feature (1.0), n_{cat} the number of electrons corresponding to the catalytic feature (2.0 for H_2O_2), i_{cat} the amount of catalytic current at the plateau, i_p the peak Faradaic current of the reversible reduction feature. Adapted.[9]

Stopped-Flow Kinetic Analysis of Ph_2Phen^{2+}

Stopped-flow spectrochemical kinetics studies were performed with a CSF-61DX2 Stopped-Flow System from Hi-Tech Scientific. Kinetic Studio Software was used to monitor a single wavelength and Integrated CCD Software was used to monitor the entire visible spectrum. All data fits were performed within the Kinetic Studio 4.0 Software Suite. Prior to experiments, dried and degassed

MeCN was passed through syringes and the cell block before reagents were loaded. In a typical experiment, syringes would be charged with known concentrations of reagent. All reagent solutions were prepared immediately before use.

In general, a vial containing **Ph_2Phen^{2+}** catalyst and acid was sparged with O_2, drawn into a syringe and loaded into the stopped-flow. A second syringe containing N_2-saturated $Cp*_2Fe$ solution was loaded into the stopped-flow. All reported concentrations are the mixed concentrations in the spectroscopic cell.

Stopped-Flow with TFAH

$$\frac{R_{fit}}{n_{cat}} = k_{cat}[Ph_2Phen^{2+}]^1[TFAH]^1[O_2]^1$$

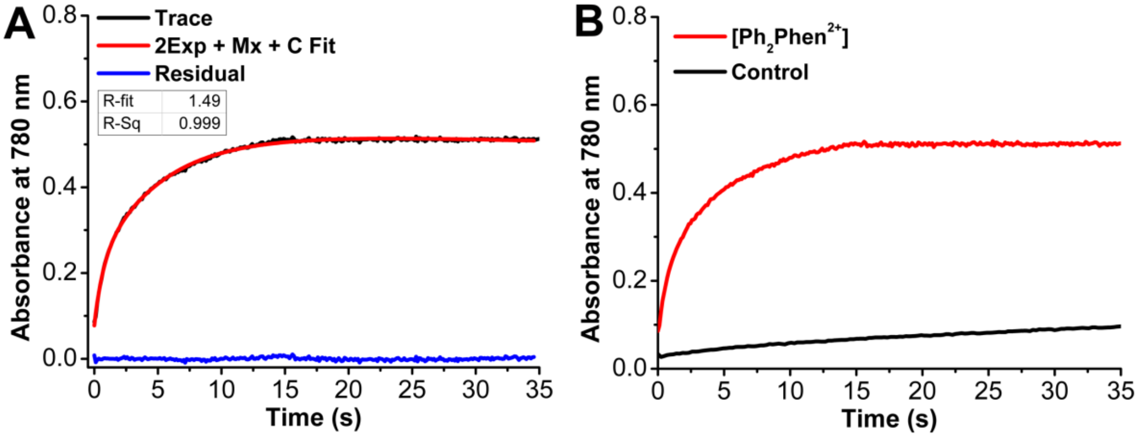

Figure S48. (A) Change in absorbance at 780 nm over time as a result of the formation of $[Cp*_2Fe]^+$ by ORR catalyzed by **Ph_2Phen^{2+}** with TFAH (black trace), example of 2Exp + Mx + C fit in Kinetic Studio 4.0 (red trace), and residual fit (blue trace). (B) Black trace from (A) with TFAH only control (no **Ph_2Phen^{2+}** present). Concentrations: **Ph_2Phen^{2+}** (if present) = 40 μM, TFAH = 50 mM, O_2 = 4.05 mM, $Cp*_2Fe$ = 1 mM.

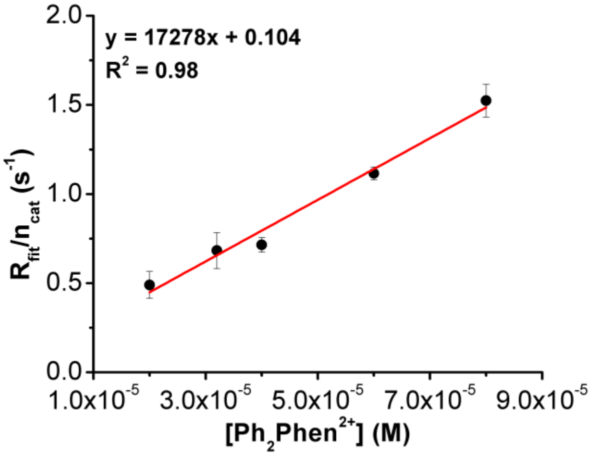

Figure S49. Calculated R_{fit}/n_{cat} values from stopped-flow spectrochemical experiments with TFAH, O_2, and $Cp*_2Fe$ with varying **Ph_2Phen^{2+}** concentration. Data were fit using Kinetic Studio 4.0 (2Exp+Mx+C); n_{cat} = 2.14. Concentrations: TFAH = 50 mM, O_2 = 4.05 mM, $Cp*_2Fe$ = 1 mM.

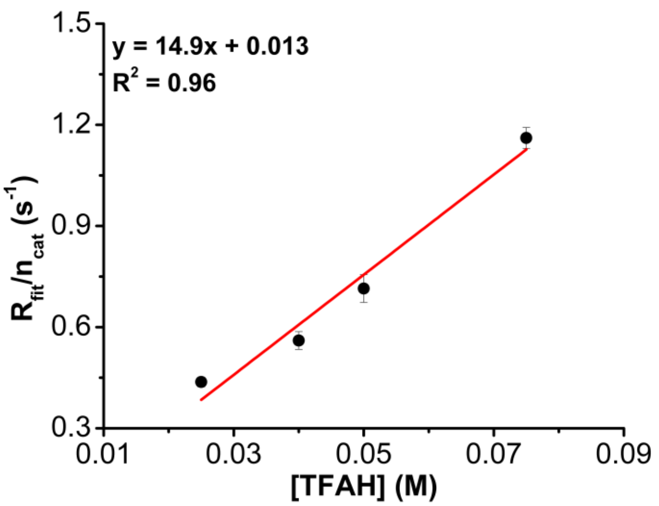

Figure S50. Calculated R_{fit}/n_{cat} values from stopped-flow spectrochemical experiments with **Ph_2Phen^{2+},** O_2, and $Cp^*{}_2Fe$ with varying TFAH concentration. Data were fit using Kinetic Studio 4.0 (2Exp+Mx+C); n_{cat} = 2.14. Concentrations: **Ph_2Phen^{2+}** = 40 μM, O_2 = 4.05 mM, $Cp^*{}_2Fe$ = 1 mM.

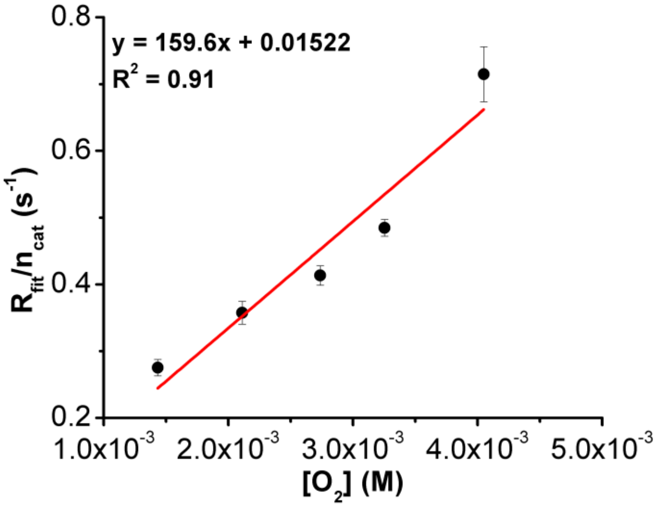

Figure S51. Calculated R_{fit}/n_{cat} values from stopped-flow spectrochemical experiments with **Ph_2Phen^{2+}**, TFAH, and $Cp^*{}_2Fe$ with varying O_2 concentration. Data were fit using Kinetic Studio 4.0 (2Exp+Mx+C); n_{cat} = 2.14. Concentrations: **Ph_2Phen^{2+}** = 40 μM, TFAH = 50 mM, $Cp^*{}_2Fe$ = 1 mM.

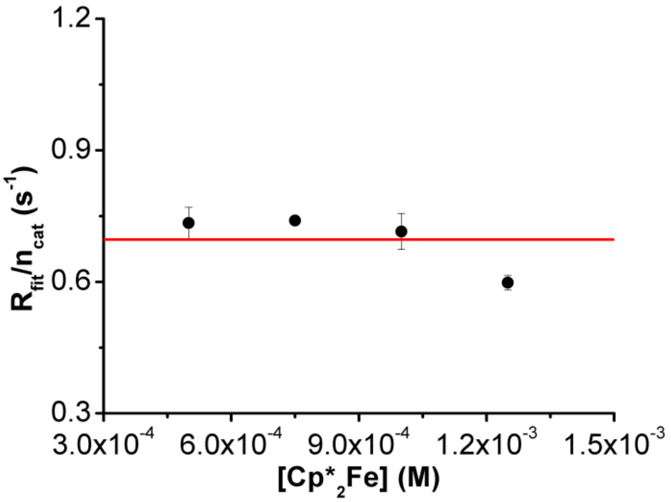

Figure S52. Calculated R_{fit}/n_{cat} values from stopped-flow spectrochemical experiments with **Ph_2Phen^{2+}**, TFAH, and O_2 with varying Cp^*_2Fe concentration. Data were fit using Kinetic Studio 4.0 (2Exp+Mx+C); n_{cat} = 2.14. The horizontal line represents the global average rate observed across all experiments for variable [Cp^*_2Fe]. Concentrations: **Ph_2Phen^{2+}** = 40 μM, TFAH = 50 mM, O_2 = 4.05 mM.

Comparison of ORR and H_2O_2RR with TFAH

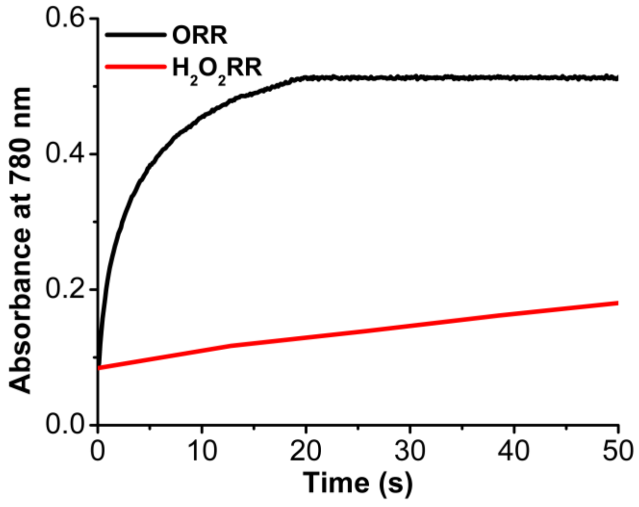

Figure S53. Change in absorbance at 780 nm over time as a result of the formation of $[Cp^*_2Fe]^+$ from the ORR (black trace) or H_2O_2RR (red trace) by **Ph_2Phen^{2+}** with TFAH. Conditions: **Ph_2Phen^{2+}** = 40 μM, TFAH = 25 mM, Cp*Fe = 1 mM, O_2 = 4.05 mM, urea•H_2O_2 = 0.96 mM.

Stopped-Flow with Cl_3AcOH

$$\frac{R_{fit}}{n_{cat}} = k_{cat}[\mathrm{Ph_2Phen^{2+}}]^1[\mathrm{Cl_3AcOH}]^1[\mathrm{O_2}]^0[Cp^*{}_2Fe]^0$$

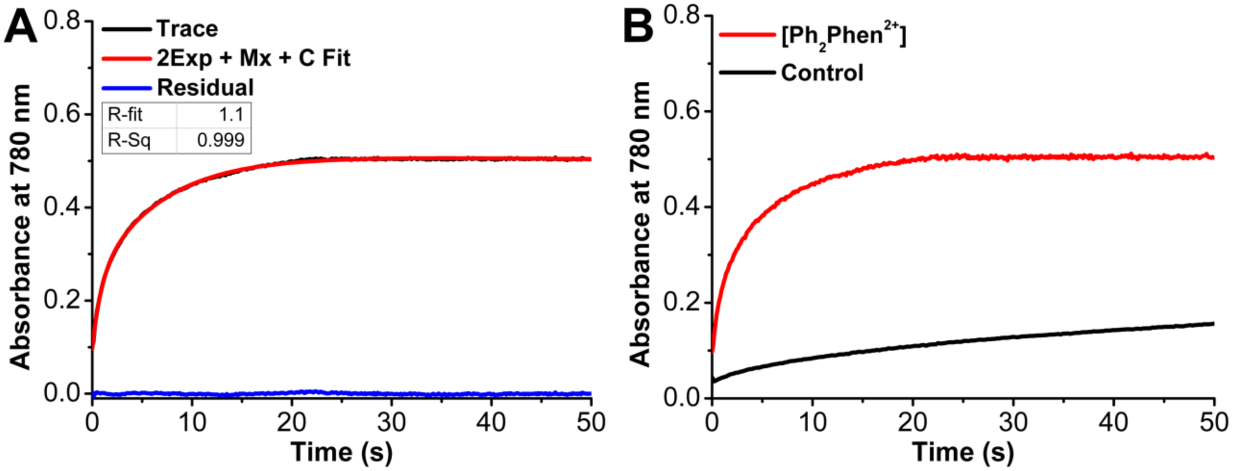

Figure S54. Change in absorbance at 780 nm over time as a result of the formation of $[Cp^*{}_2Fe]^+$ by ORR catalyzed by **Ph_2Phen^{2+}** with Cl_3AcOH (black trace), example of 2Exp + Mx + C fit in Kinetic Studio 4.0 (red trace), and residual fit (blue trace). (B) Black trace from (A) with Cl_3AcOH only control (no **Ph_2Phen^{2+}** present). Concentrations: **Ph_2Phen^{2+}** = 40 μM, Cl_3AcOH = 50 mM, O_2 = 4.05 mM, $Cp^*{}_2Fe$ = 1 mM.

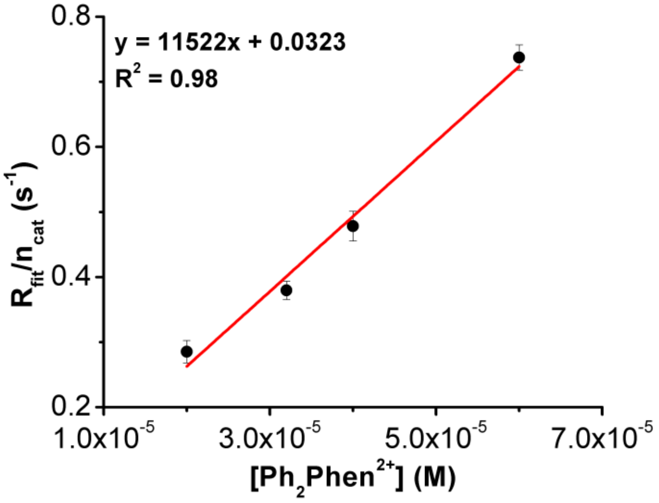

Figure S55. Calculated R_{fit}/n_{cat} values from stopped-flow spectrochemical experiments with Cl_3AcOH, O_2, and $Cp^*{}_2Fe$ with varying **Ph_2Phen^{2+}** concentration. Data were fit using Kinetic Studio 4.0 (2Exp+Mx+C); n_{cat} = 2.3. Concentrations: Cl_3AcOH = 50 mM, O_2 = 4.05 mM, $Cp^*{}_2Fe$ = 1 mM.

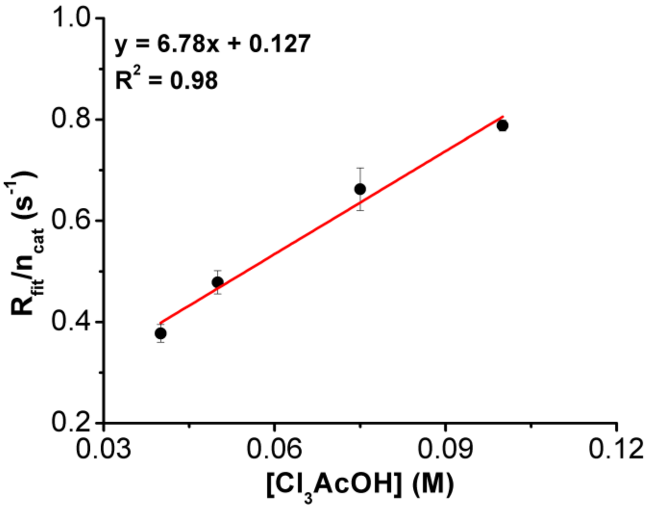

Figure S56. Calculated R_{fit}/n_{cat} values from stopped-flow spectrochemical experiments with **Ph_2Phen^{2+}**, O_2, and $Cp^*{}_2Fe$ with varying Cl_3AcOH concentration. Data were fit using Kinetic Studio 4.0 (2Exp+Mx+C); n_{cat} = 2.3. Concentrations: **Ph_2Phen^{2+}** = 40 μM, O_2 = 4.05 mM, $Cp^*{}_2Fe$ = 1 mM.

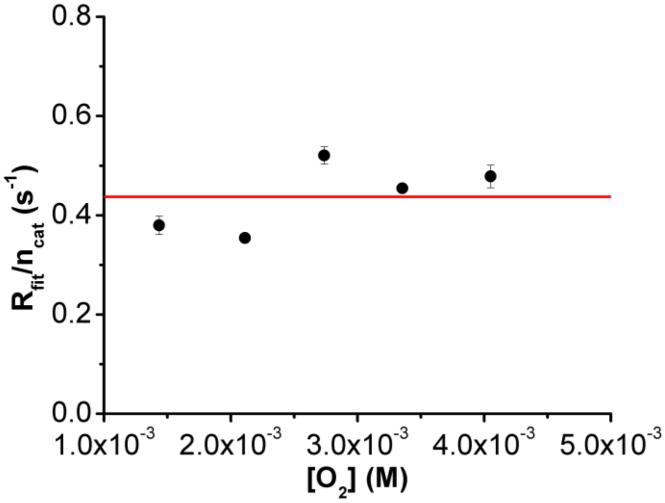

Figure S57. Calculated R_{fit}/n_{cat} values from stopped-flow spectrochemical experiments with **Ph_2Phen^{2+}**, Cl_3AcOH, and $Cp^*{}_2Fe$ with varying O_2 concentration. Data were fit using Kinetic Studio 4.0 (2Exp+Mx+C); *n_{cat} = 2.3.* The horizontal line represents the global average rate observed across all experiments for variable [O_2]. Concentrations: **Ph_2Phen^{2+}** = 40 μM, Cl_3AcOH = 50 mM, $Cp^*{}_2Fe$ = 1 mM.

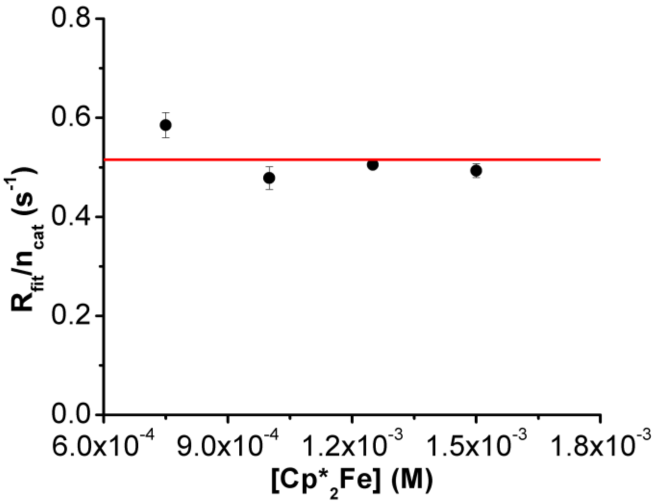

Figure S58. Calculated R_{fit}/n_{cat} values from stopped-flow spectrochemical experiments with **Ph_2Phen^{2+}**, Cl_3AcOH, and O_2 with varying Cp^*_2Fe concentration. Data were fit using Kinetic Studio 4.0 (2Exp+Mx+C); n_{cat} = 2.3. The horizontal line represents the global average rate observed across all experiments for variable [Cp^*_2Fe]. Concentrations: **Ph_2Phen^{2+}** = 40 μM, Cl_3AcOH = 50 mM, O_2 = 4.05 mM.

Comparison of ORR and H_2O_2RR with Cl_3AcOH

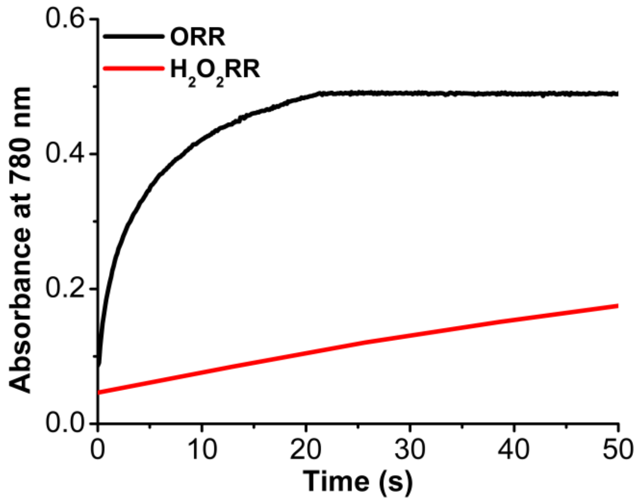

Figure S59. Change in absorbance at 780 nm over time of [Cp^*_2Fe]$^+$ over time as a result of the ORR (black trace) or H_2O_2RR (red trace) by **Ph_2Phen^{2+}** with Cl_3AcOH. Conditions: **Ph_2Phen^{2+}** = 40 μM, Cl_3AcOH = 25 mM, Cp*Fe = 1 mM, O_2 = 4.05 mM, urea•H_2O_2 = 0.96 mM.

Stopped-Flow with Cl_2AcOH

$$\frac{R_{fit}}{n_{cat}} = k_{cat}[Ph_2Phen^{2+}]^{1/2}[Cl_2AcOH]^1[O_2]^0[Cp^*{}_2Fe]^0$$

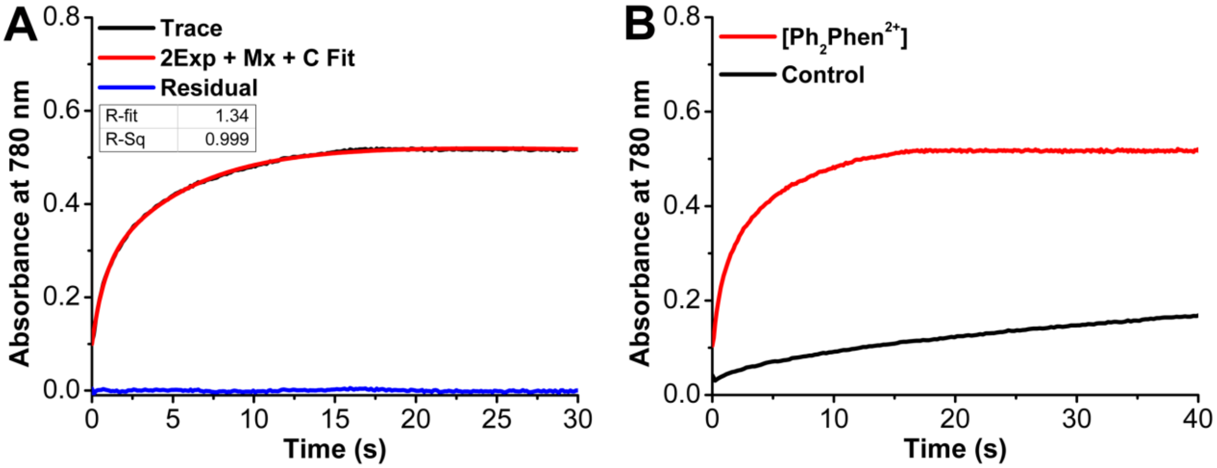

Figure S60. (A) Change in absorbance at 780 nm over time as a result of the formation of $[Cp^*{}_2Fe]^+$ by ORR catalyzed by **Ph_2Phen^{2+}** with Cl_2AcOH (black trace), example of 2Exp + Mx + C fit in Kinetic Studio 4.0 (red trace), and residual fit (blue trace). (B) Black trace from (A) with Cl_2AcOH only control (no **Ph_2Phen^{2+}** present). Concentrations: **Ph_2Phen^{2+}** = 40 μM, Cl_2AcOH = 50 mM, O_2 = 4.05 mM, $Cp^*{}_2Fe$ = 1 mM.

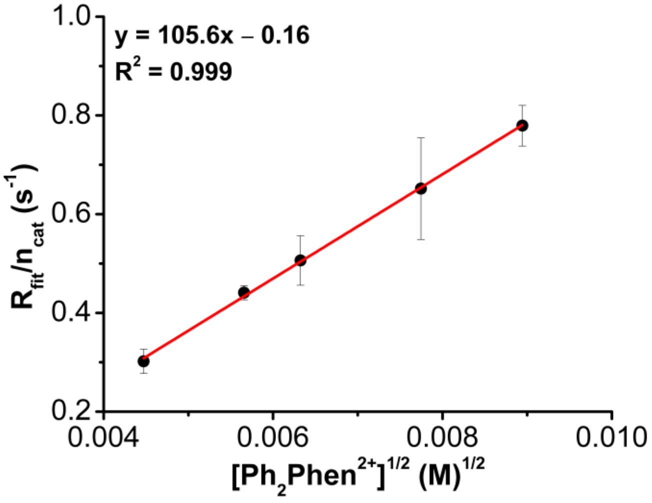

Figure S61. Calculated R_{fit}/n_{cat} values from stopped-flow spectrochemical experiments with Cl_2AcOH, O_2, and $Cp^*{}_2Fe$ with varying **Ph_2Phen^{2+}** concentration. Data were fit using Kinetic Studio 4.0 (2Exp+Mx+C); n_{cat} = 2.35. Concentrations: Cl_2AcOH = 50 mM, O_2 = 4.05 mM, $Cp^*{}_2Fe$ = 1 mM.

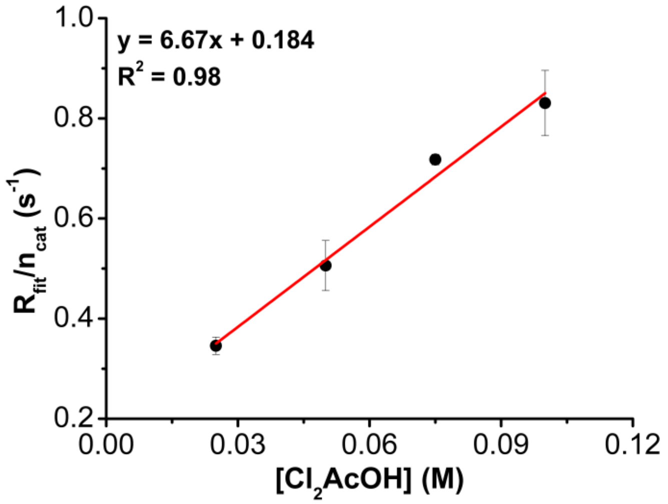

Figure S62. Calculated R_{fit}/n_{cat} values from stopped-flow spectrochemical experiments with **Ph_2Phen^{2+}**, O_2, and $Cp^*{}_2Fe$ with varying Cl_2AcOH concentration. Data were fit using Kinetic Studio 4.0 (2Exp+Mx+C); n_{cat} = 2.35. Concentrations: **Ph_2Phen^{2+}** = 40 μM, O_2 = 4.05 mM, $Cp^*{}_2Fe$ = 1 mM.

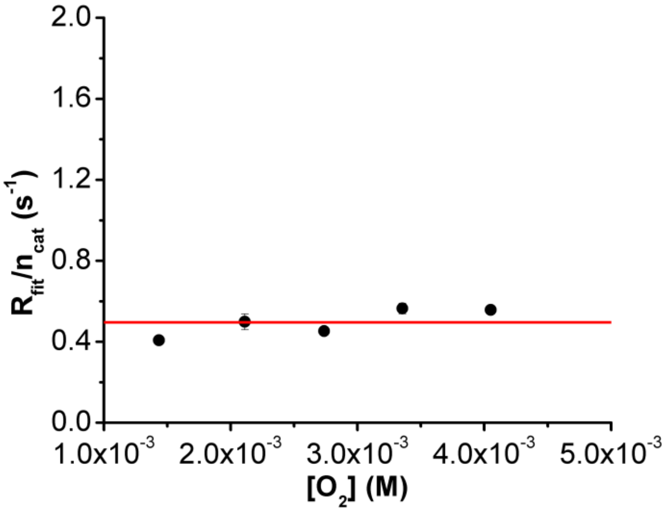

Figure S63. Calculated R_{fit}/n_{cat} values from stopped-flow spectrochemical experiments with **Ph_2Phen^{2+}**, Cl_2AcOH, and $Cp^*{}_2Fe$ with varying O_2 concentration. Data were fit using Kinetic Studio 4.0 (2Exp+Mx+C); n_{cat} = 2.35. The horizontal line represents the global average rate observed across all experiments for variable [O_2]. Concentrations: **Ph_2Phen^{2+}** = 40 μM, Cl_2AcOH = 50 mM, $Cp^*{}_2Fe$ = 1 mM.

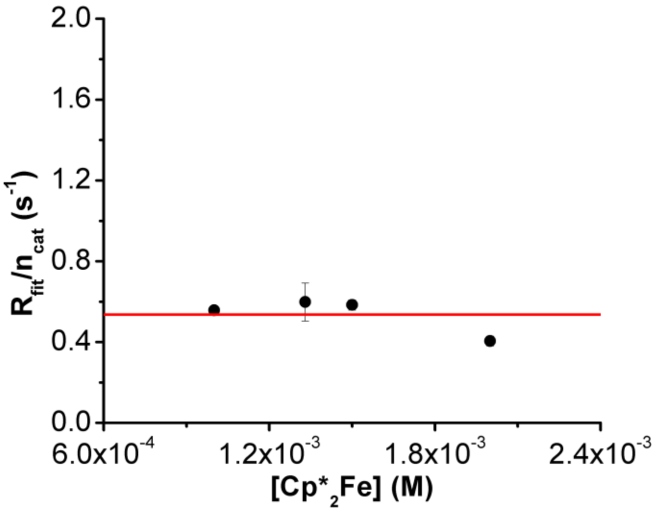

Figure S64. Calculated R_{fit}/n_{cat} values from stopped-flow spectrochemical experiments with **Ph_2Phen^{2+}**, Cl_2AcOH, and O_2 with varying $Cp^*{}_2Fe$ concentration. Data were fit using Kinetic Studio 4.0 (2Exp+Mx+C); n_{cat} = 2.35. The horizontal line represents the global average rate observed across all experiments for variable [$Cp^*{}_2Fe$]. Concentrations: **Ph_2Phen^{2+}** = 40 μM, Cl_2AcOH = 50 mM, O_2 = 4.05 mM.

Comparison of ORR and H_2O_2RR with Cl_2AcOH

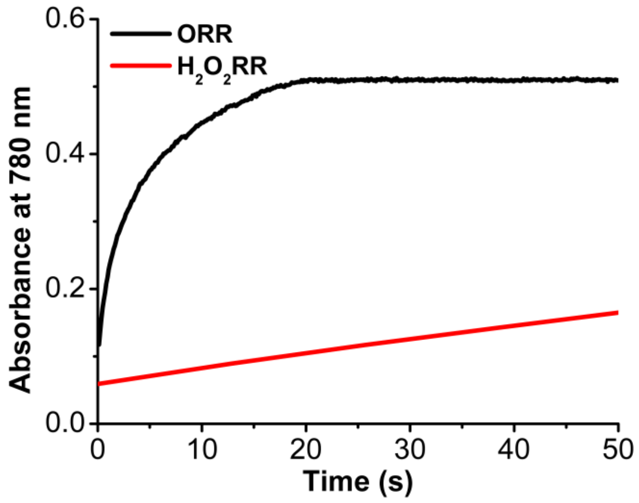

Figure S65. Change in absorbance at 780 nm over time as a result of the formation of $[Cp^*{}_2Fe]^+$ from the ORR (black trace) or H_2O_2RR (red trace) by **Ph_2Phen^{2+}** with Cl_2AcOH. Conditions: **Ph_2Phen^{2+}** = 40 μM, Cl_2AcOH = 25 mM, Cp*Fe = 1 mM, O_2 = 4.05 mM, urea•H_2O_2 = 0.96 mM.

Stopped-Flow with ClAcOH

$$\text{Rate} = k_{cat}[\text{Ph}_2\text{Phen}^{2+}]^{0.5}[\text{ClAcOH}]^1[\text{O}_2]^0[Cp^*{}_2Fe]^0$$

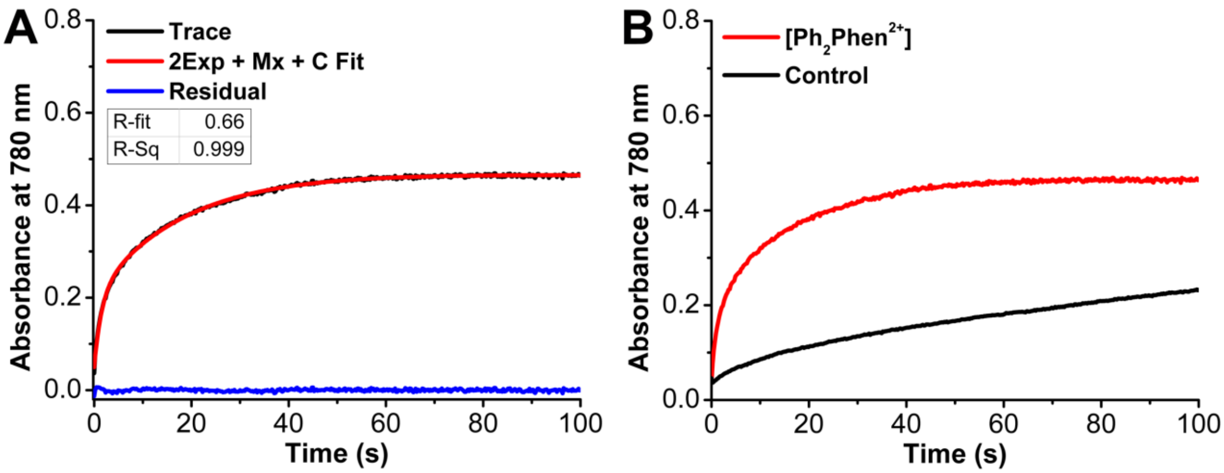

Figure S66. Change in absorbance at 780 nm over time as a result of the formation of $[Cp^*{}_2Fe]^+$ by ORR catalyzed by **Ph_2Phen^{2+}** with ClAcOH (black trace), example of 2Exp + Mx + C fit in Kinetic Studio 4.0 (red trace), and residual fit (blue trace). (B) Black trace from (A) with ClAcOH only control (no **Ph_2Phen^{2+}** present). Concentrations: **Ph_2Phen^{2+}** = 40 μM, ClAcOH = 50 mM, O_2 = 4.05 mM, $Cp^*{}_2Fe$ = 1 mM.

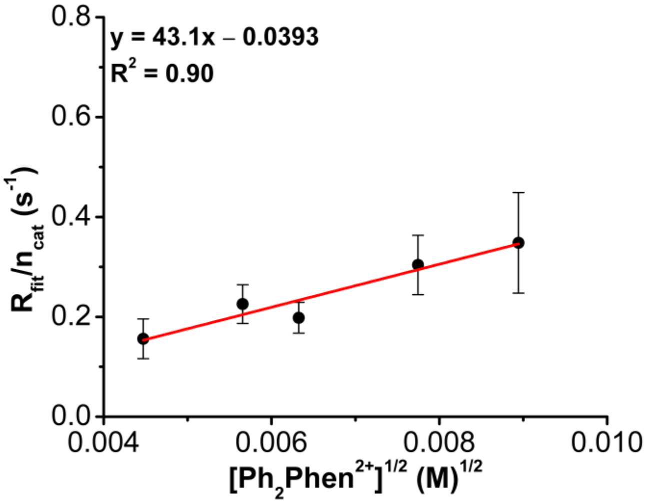

Figure S67. Calculated R_{fit}/n_{cat} values from stopped-flow spectrochemical experiments with ClAcOH, O_2, and $Cp^*{}_2Fe$ with varying **Ph_2Phen^{2+}** concentration. Data were fit using Kinetic Studio 4.0 (2Exp+Mx+C); n_{cat} = 3.52. Concentrations: ClAcOH = 50 mM, O_2 = 4.05 mM, $Cp^*{}_2Fe$ = 1 mM.

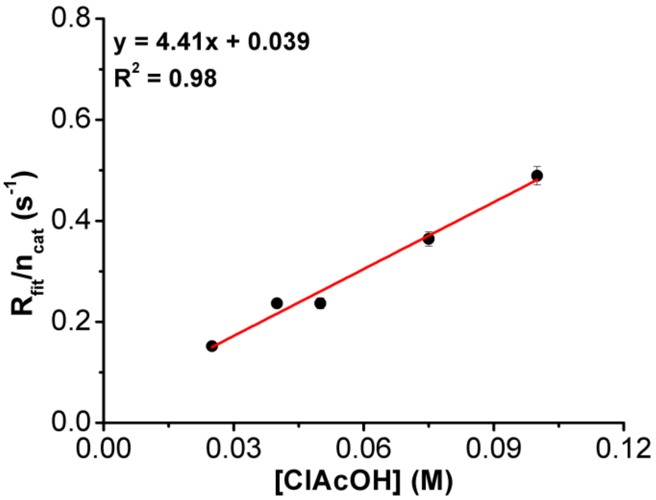

Figure S68. Calculated R_{fit}/n_{cat} values from stopped-flow spectrochemical experiments with **Ph_2Phen^{2+}**, O_2, and $Cp^*{}_2Fe$ with varying ClAcOH concentration. Data were fit using Kinetic Studio 4.0 (2Exp+Mx+C); n_{cat} = 3.52. Concentrations: **Ph_2Phen^{2+}** = 40 μM, O_2 = 4.05 mM, $Cp^*{}_2Fe$ = 1 mM.

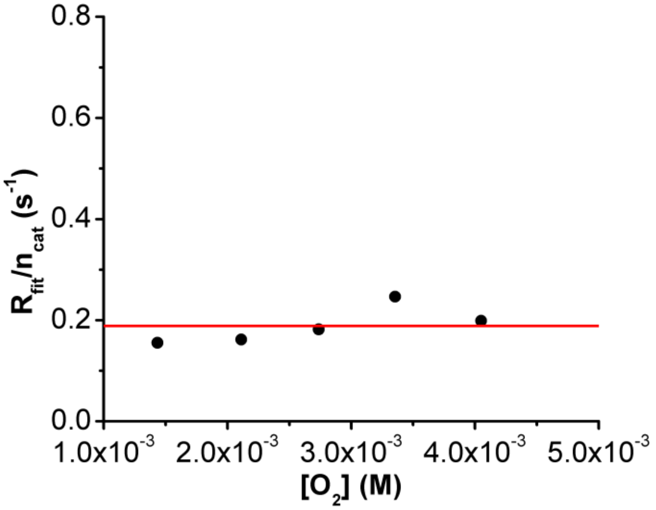

Figure S69. Calculated R_{fit}/n_{cat} values from stopped-flow spectrochemical experiments with **Ph_2Phen^{2+}**, ClAcOH, and $Cp^*{}_2Fe$ with varying O_2 concentration. Data were fit using Kinetic Studio 4.0 (2Exp+Mx+C); n_{cat} = 3.52. The horizontal line represents the global average rate observed across all experiments for variable [O_2]. Concentrations: **Ph_2Phen^{2+}** = 40 μM, ClAcOH = 50 mM, $Cp^*{}_2Fe$ = 1 mM.

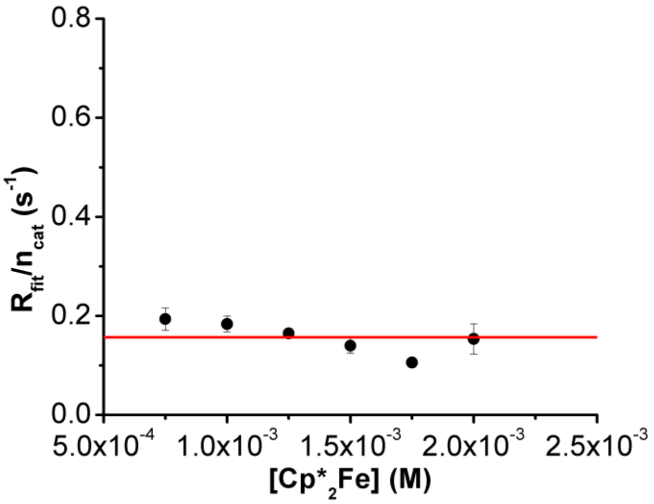

Figure S70. Calculated R_{fit}/n_{cat} values from stopped-flow spectrochemical experiments with **Ph_2Phen^{2+}**, ClAcOH, and O_2 with varying Cp*$_2$Fe concentration. Data were fit using Kinetic Studio 4.0 (2Exp+Mx+C); n_{cat} = 3.52. The horizontal line represents the global average rate observed across all experiments for variable [Cp*$_2$Fe]. Concentrations: **Ph_2Phen^{2+}** = 40 μM, ClAcOH = 50 mM, O_2 = 4.05 mM.

Comparison of ORR and H_2O_2RR with ClAcOH

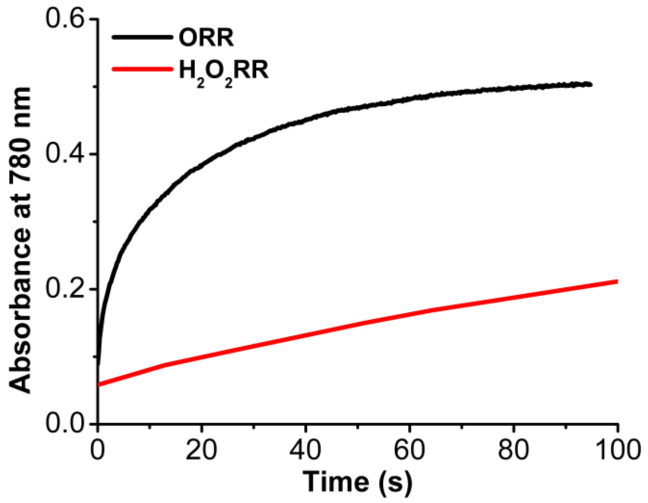

Figure S71. Change in absorbance at 780 nm over time as a result of the formation of [Cp*$_2$Fe]$^+$ from the ORR (black trace) or H_2O_2RR (red trace) by **Ph_2Phen^{2+}** with ClAcOH. Conditions: **Ph_2Phen^{2+}** = 40 μM, ClAcOH = 25 mM, Cp*Fe = 1 mM, O_2 = 4.05 mM, urea•H_2O_2 = 0.96 mM.

Spectrochemical Analysis

ORR Selectivity

The ORR selectivity of **Ph_2Phen^{2+}** was determined using a previously reported procedure.[5] Generally, solutions containing 80 μM [**Ph_2Phen^{2+}**] and 50 mM acid (TFAH, Cl_3AcOH, Cl_2AcOH, or ClAcOH) were sparged with O_2 gas and rapidly mixed in a 1:1 ratio with a N_2 saturated 2 mM $Cp^*{}_2Fe$ solution to a final volume of 4 mL (final concentrations: 40 μM **Ph_2Phen^{2+},** 1 mM $Cp^*{}_2Fe$, 25 mM acid, 4.05 mM O_2). The reactions were allowed to reach completion and 2 mL of the reaction solution was added to 2 mL of water. Then, 3 mL of this solution was added to the cuvette and a UV-vis spectrum was taken before and after the addition of 0.1 mL of $Ti(O)SO_4$ solution, as previously reported.[5, 6, 10-12] A calibration curve was used to establish **Eqs S21-S22**[5] and were used to calculate the % selectivity of H_2O_2.

$$O_2 + 2Cp^*{}_2Fe(1\ mM) + 2AH \xrightarrow{cat.[Ph_2Phen^{2+}]} H_2O_2(0.5\ mM) + 2[Cp^*{}_2Fe]^+ + 2A^- \quad \textbf{(Eq S21)}$$

Abs@408 nm (red trace) – Abs@408 nm (black trace) = $405.6[H_2O_2]_{exp} - 0.01122$

$$\frac{[H_2O_2]_{exp}}{0.5\ mM\ H_2O_2} \times 100 = \%\ H_2O_2\ \text{selectivity} \quad \textbf{(Eq S22)}$$

Table S3. Summary of ORR selectivity by **Ph_2Phen^{2+}** with each acid.[a]

	Time (s)[b]	% H_2O_2	% H_2O	n_{cat}
**TFAH**	90	93.2 ± 1.4	6.8 ± 1.4	2.14
**Cl_3AcOH**	90	84.8 ± 5.8	15.2 ± 5.8	2.30
**Cl_2AcOH**	120	82.3 ± 3.2	17.7 ± 3.2	2.35
**ClAcOH**	180	24.0 ± 6.2	76.0 ± 6.2	3.52

[a] 40 μM [**Ph_2Phen^{2+}**], 25 mM [AH], 4.05 mM [O_2], 1 mM [$Cp^*{}_2Fe$]; [b]time when all Cp^*Fe_2 is consumed

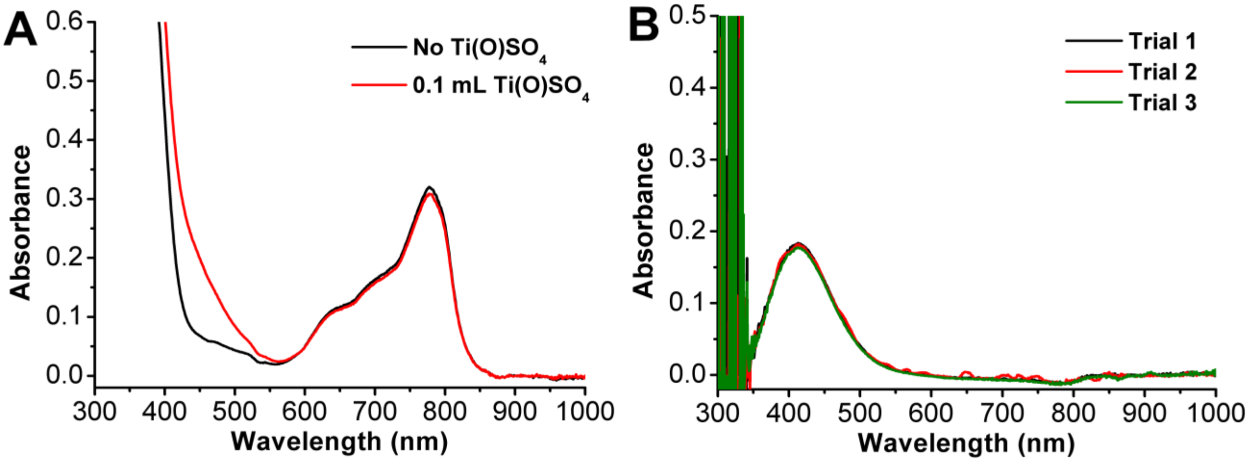

Figure S72. H_2O_2 product quantification of ORR by **Ph_2Phen^{2+}** with TFAH after 90 s. (A) UV-vis spectra of catalytic aliquot before (black) and after (red) 0.1 mL of 0.1 M $Ti(O)SO_4$ solution was added. (B) Corrected spectra (red – black trace from A). Conditions: 40 μM **Ph_2Phen^{2+}**, 25 mM TFAH, 1 mM $Cp^*{}_2Fe$, 4.05 mM O_2 in MeCN.

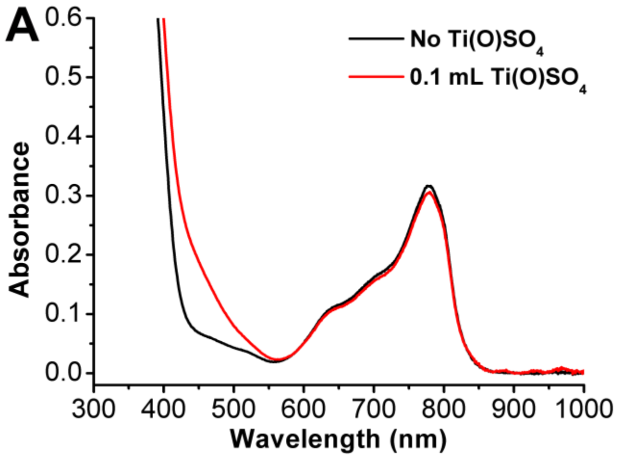

Figure S73. H_2O_2 product quantification of ORR by **Ph_2Phen^{2+}** with Cl_3AcOH after 90 s. (A) UV-vis spectra of catalytic aliquot before (black) and after (red) 0.1 mL of 0.1 M $Ti(O)SO_4$ solution was added. (B) Corrected spectra (red – black trace from A). Conditions: 40 μM **Ph_2Phen^{2+}**, 25 mM Cl_3AcOH, 1 mM Cp^*_2Fe, 4.05 mM O_2 in MeCN.

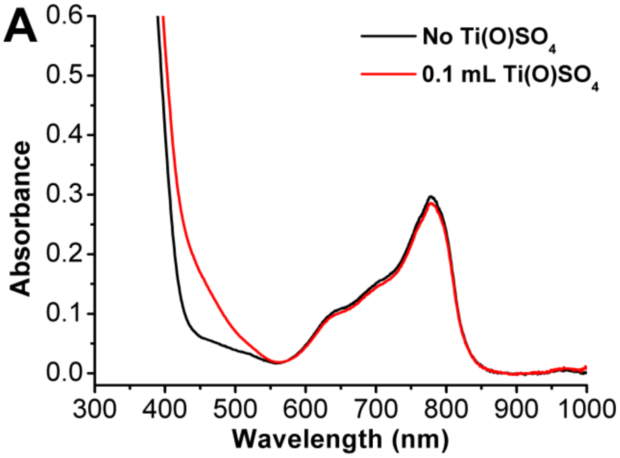

Figure S74. H_2O_2 product quantification of ORR by **Ph_2Phen_{2+}** with Cl_2AcOH after 2 min. (A) UV-vis spectra of catalytic aliquot before (black) and after (red) 0.1 mL of 0.1 M $Ti(O)SO_4$ solution was added. (B) Corrected spectra (red – black trace from A). Conditions: 40 μM **Ph_2Phen^{2+}**, 25 mM Cl_2AcOH, 1 mM Cp^*_2Fe, 4.05 mM O_2 in MeCN.

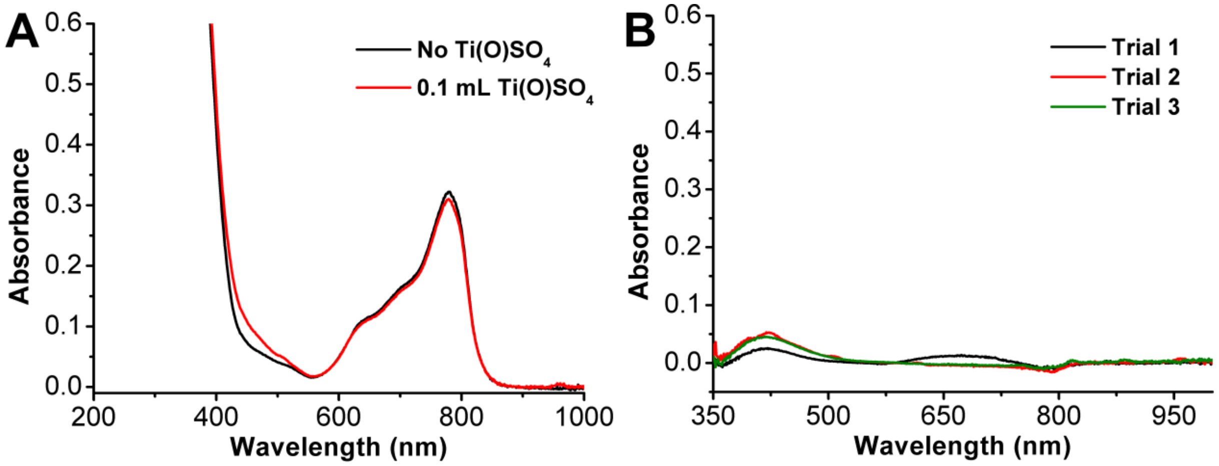

Figure S75. H_2O_2 product quantification of ORR by **Ph_2Phen^{2+}** with ClAcOH after 3 min. (A) UV-vis spectra of catalytic aliquot before (black) and after (red) 0.1 mL of 0.1 M $Ti(O)SO_4$ solution was added. (B) Corrected spectra (red – black trace from A). Conditions: 40 μM **Ph_2Phen^{2+}**, 25 mM ClAcOH, 1 mM $Cp^*{}_2Fe$, 4.05 mM O_2 in MeCN.

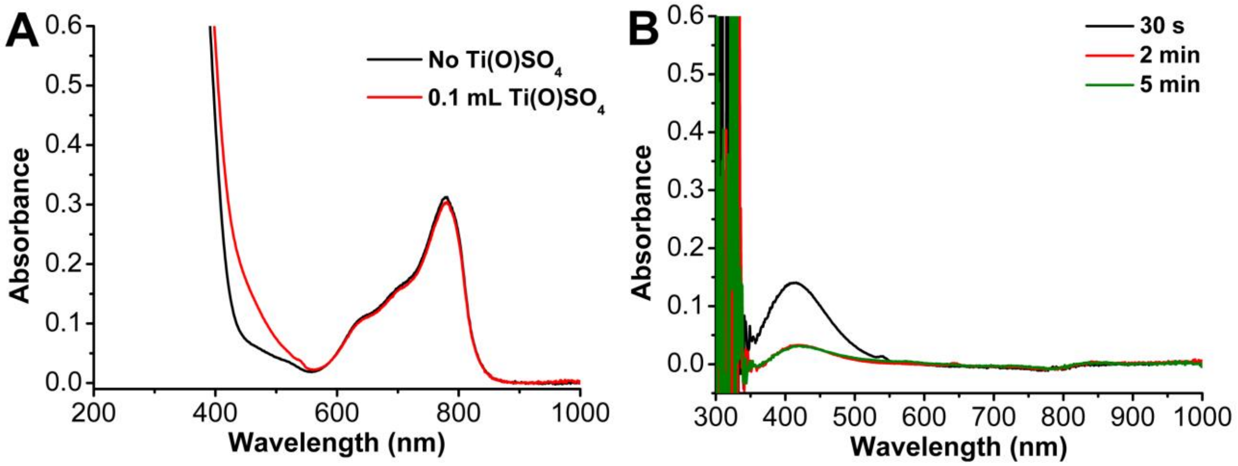

Figure S76. H_2O_2 product quantification of ORR by **Ph_2Phen^{2+}** with ClAcOH after 30 s, 2 min, and 5 min. (A) UV-vis spectra of catalytic aliquot before (black) and after (red) 0.1 mL of 0.1 M $Ti(O)SO_4$ solution was added after 30 s. (B) Corrected spectra (red – black trace from A) after 30 s, 2 min, and 5 min. Conditions: 40 μM **Ph_2Phen^{2+}**, 25 mM ClAcOH, 1 mM $Cp^*{}_2Fe$, 4.05 mM O_2 in MeCN.

Table S4. Summary of ORR selectivity of **Ph_2Phen^{2+}** with ClAcOH at various time points from **Figure S76**.[a]

Time (s)	% H_2O_2
30	67.4 ± 5.9
120	20.6 ± 2.8
300	22.0 ± 5.2

[a]40 μM [**Ph_2Phen^{2+}**], 25 mM [ClAcOH], 4.05 mM [O_2], 1 mM [$Cp^*{}_2Fe$]

H_2O_2 Stability Control Studies

To determine the stability of H_2O_2 in the presence of **Ph_2Phen^{2+}**, acid, and O_2, control studies were conducted. Generally, solutions containing 80 μM [**Ph_2Phen^{2+}** and 50 mM acid (TFAH, Cl_3AcOH, Cl_2AcOH, or ClAcOH) were sparged with O_2 gas and rapidly mixed in a 1:1 ratio with a N_2 saturated 1.76 mM urea•H_2O_2 solution to a final volume of 4 mL (final concentrations: 40 μM **Ph_2Phen^{2+},** 0.88 mM urea•H_2O_2, 25 mM acid, 4.05 mM O_2). The reactions were allowed to reach completion and 2 mL of the reaction solution was added to 2 mL of water. A UV-vis spectrum was taken before and after the addition of 0.1 mL of 0.1 M $Ti(O)SO_4$ solution and the difference at 408 nm was used to determine the amount of H_2O_2 present ($[H_2O_2]_{detected}$). The % recovery was determined according to **Eq S23** from measured $[H_2O_2]_{expected}$ of the H_2O_2 stock solution.

$$\frac{[H_2O_2]_{detected}}{[H_2O_2]_{expected}} \times 100 = \%\ H_2O_2\ recovery \qquad \textbf{(Eq S23)}$$

Table S5. Summary of H_2O_2 Recovery for Stability Control Studies by **Ph_2Phen^{2+}** with each acid.[a]

	**% H_2O_2 Recovered**
**TFAH**	97.6 ± 0.88
**Cl_3AcOH**	101 ± 5.0
**Cl_2AcOH**	95.5 ± 1.7
**ClAcOH**	94.3 ± 2.9

[a] 40 μM [**Ph_2Phen^{2+}**], 25 mM [AH], 4.05 mM [O_2], 0.88 mM [urea•H_2O_2]

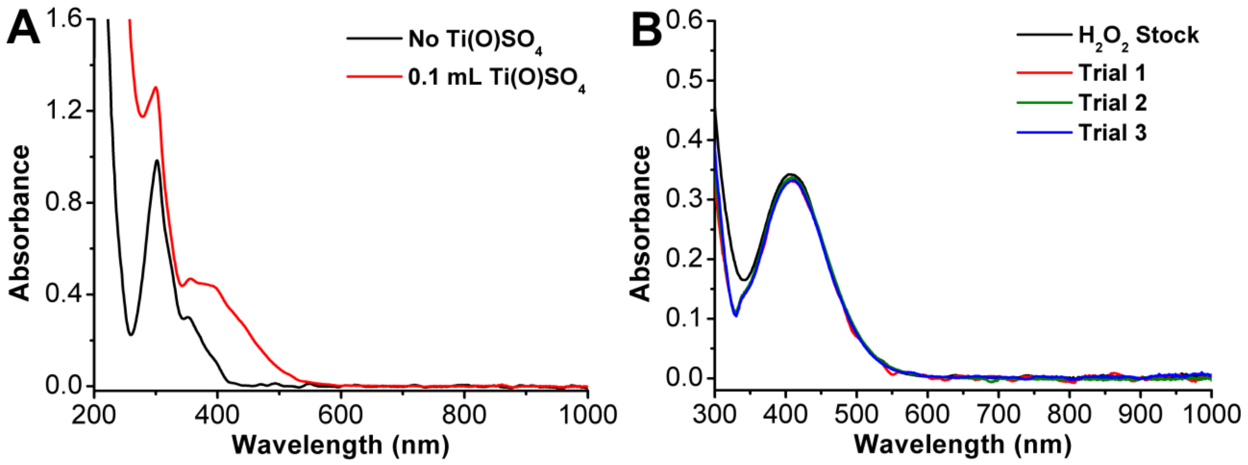

Figure S77. Stability test of urea•H_2O_2 in the presence of **Ph_2Phen^{2+}**, TFAH, and O_2 (A) before (black trace) and after (red trace) the addition of 0.1 mL of 0.1 M $Ti(O)SO_4$ to an aliquot. (B) Corrected UV-vis spectra (red – black from A) of each trial after 90 s with the H_2O_2 stock (black). Conditions: 40 μM Ph2Phen2+, 25 mM TFAH, 4.05 mM O2, 0.88 mM urea•H_2O_2 in MeCN.

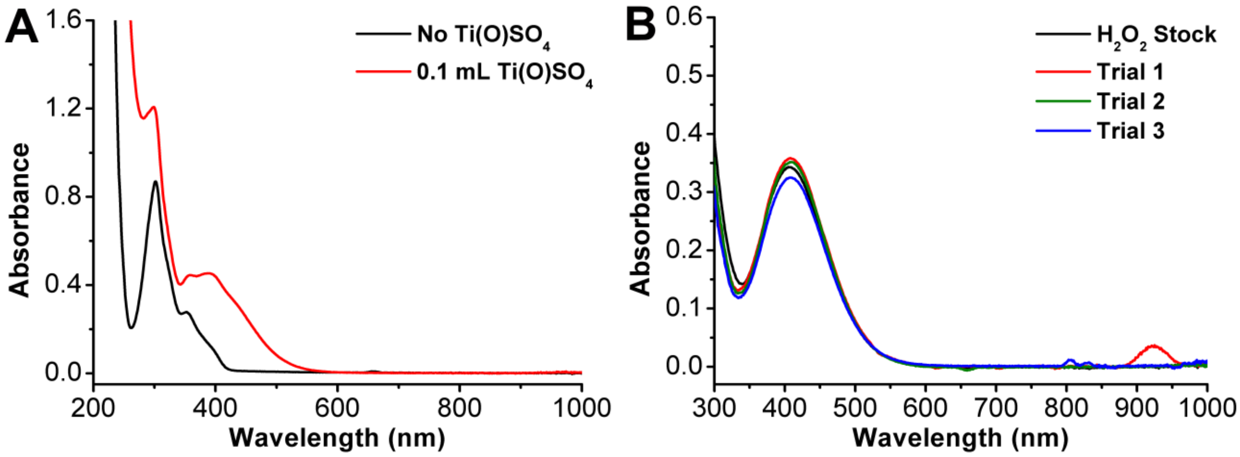

Figure S78. Stability test of urea•H_2O_2 in the presence of **Ph_2Phen^{2+}**, Cl_3AcOH, and O_2 (A) before (black trace) and after (red trace) the addition of 0.1 mL of 0.1 M Ti(O)SO4 to an aliquot. (B) Corrected UV-vis spectra (red – black from A) of each trial after 90 s with the H_2O_2 stock (black). Conditions: 40 µM **Ph_2Phen^{2+}**, 25 mM Cl_3AcOH, 4.05 mM O_2, 0.88 mM urea•H_2O_2 in MeCN.

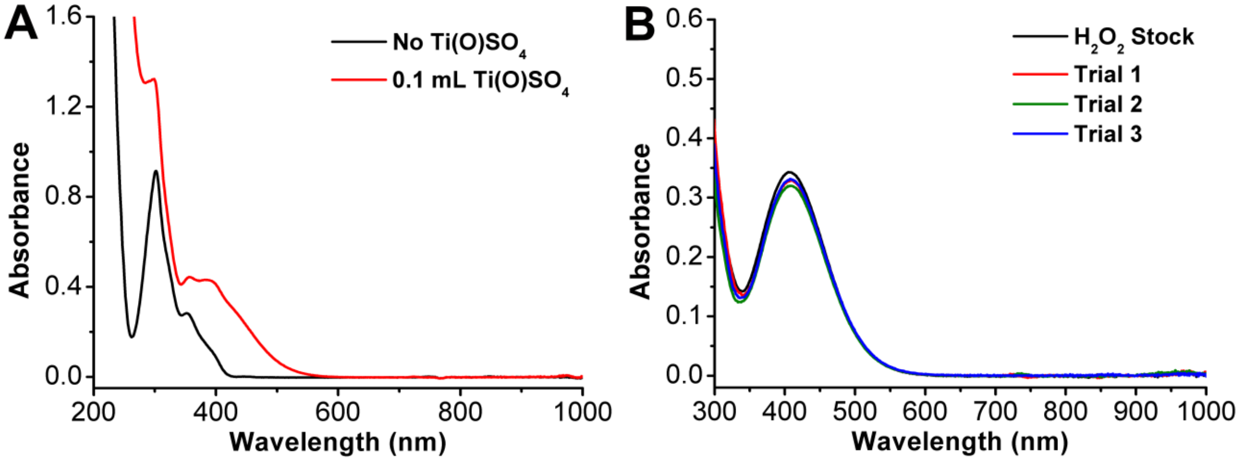

Figure S79. Stability test of urea•H_2O_2 in the presence of **Ph_2Phen^{2+}**, Cl_2AcOH, and O_2 (A) before (black trace) and after (red trace) the addition of 0.1 mL of 0.1 M $Ti(O)SO_4$ to an aliquot. (B) Corrected UV-vis spectra (red – black from A) of each trial after 2 min with the H_2O_2 stock (black). Conditions: 40 µM Ph_2Phen^{2+}, 25 mM Cl_2AcOH, 4.05 mM O_2, 0.88 mM urea•H_2O_2 in MeCN.

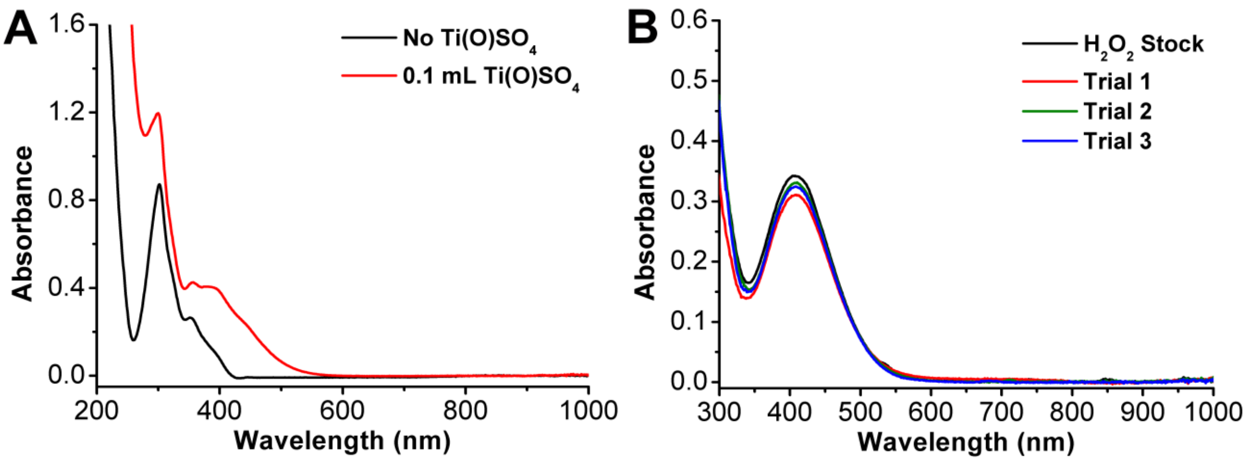

Figure S80. Stability test of urea•H_2O_2 in the presence of **Ph_2Phen^{2+}**, ClAcOH, and O_2 (A) before (black trace) and after (red trace) the addition of 0.1 mL of 0.1 M $Ti(O)SO_4$ to an aliquot. (B) Corrected UV-vis spectra (red – black from A) of each trial after 3 min with the H_2O_2 stock (black). Conditions: 40 μM Ph_2Phen^{2+}, 25 mM ClAcOH, 4.05 mM O_2, 0.88 mM urea•H_2O_2 in MeCN.

Computational Methods

Geometry optimization was done with the Gaussian 16 package[13] at the B3LYP-D3(BJ)/def2-TZVP level[14-21] with a complete structural model. Dispersion and bulk solvent effects (acetonitrile = MeCN; ε = 35.688) were accounted for at the optimization stage, by using Grimme's D3 parameter set with Becke-Johnson (BJ) damping[20, 21] and the SMD continuum model,[22] respectively. The stationary points and their nature as minima (no imaginary frequencies) were characterized by vibrational analysis using the IGRRHO approach as implemented by default in the software package, which also produced enthalpy (H), entropy (S) and Gibbs energy (G) data at 298.15 K. Transition states were generally identified at the same level of theory and all were characterized by a single imaginary frequency corresponding to the forming/cleaving bonds. The doublet monocation **$[ROOR]^+$** and the dimer cleavage transition states **$[ROOR•HA]^{2+\ ‡}$** for TFAH and ClAcOH were too computationally costly and instead located at the SVP level. The radical-ion pair was identified through a potential energy surface scan and estimated by using a constrained carbon-oxygen bond distance, with all other atoms free to equilibrate.

Free energies were corrected (ΔG_{qh}) to account for concentration effects and for errors associated with the harmonic oscillator approximation. Thus, according to Truhlars's quasi-harmonic approximation for vibrational entropy and enthalpy, all vibrational frequencies below 100 cm^{-1} were set to this value.[23] These anharmonic and concentration corrections were calculated with the Goodvibes code.[24] Concentrations were set at 0.001 M for all species unless otherwise indicated, 0.004 M for O_2, 0.500 M for acid, and 18.9 M for MeCN. Single point calculations for refining energy differences were completed with Orca 5.0[25] at the DLPNO-CCSD(T1)/cc-pVTZ level.[19, 26, 27] Both **$[ROOR]^+$** and the dimer cleavage transition states **$[ROOR•HA]^{2+\ ‡}$** for TFAH and ClAcOH were refined first in Gaussian at the TZVP level before additional CCSD refinement. Evaluation of spin density was done at the ωB97M-D4/def2-TZVPPD level.[18, 19, 28-32] The stability of the wavefunction and spin contamination were studied at the triple-zeta level of theory. Reduction potentials from computational data were obtained according to our previous methodology by using the calculated free energy of reduction of the species of interest by $[phenazine]^-$, corrected to the experimental potential of phenazine reduction in MeCN vs Fc^+/Fc.[33]

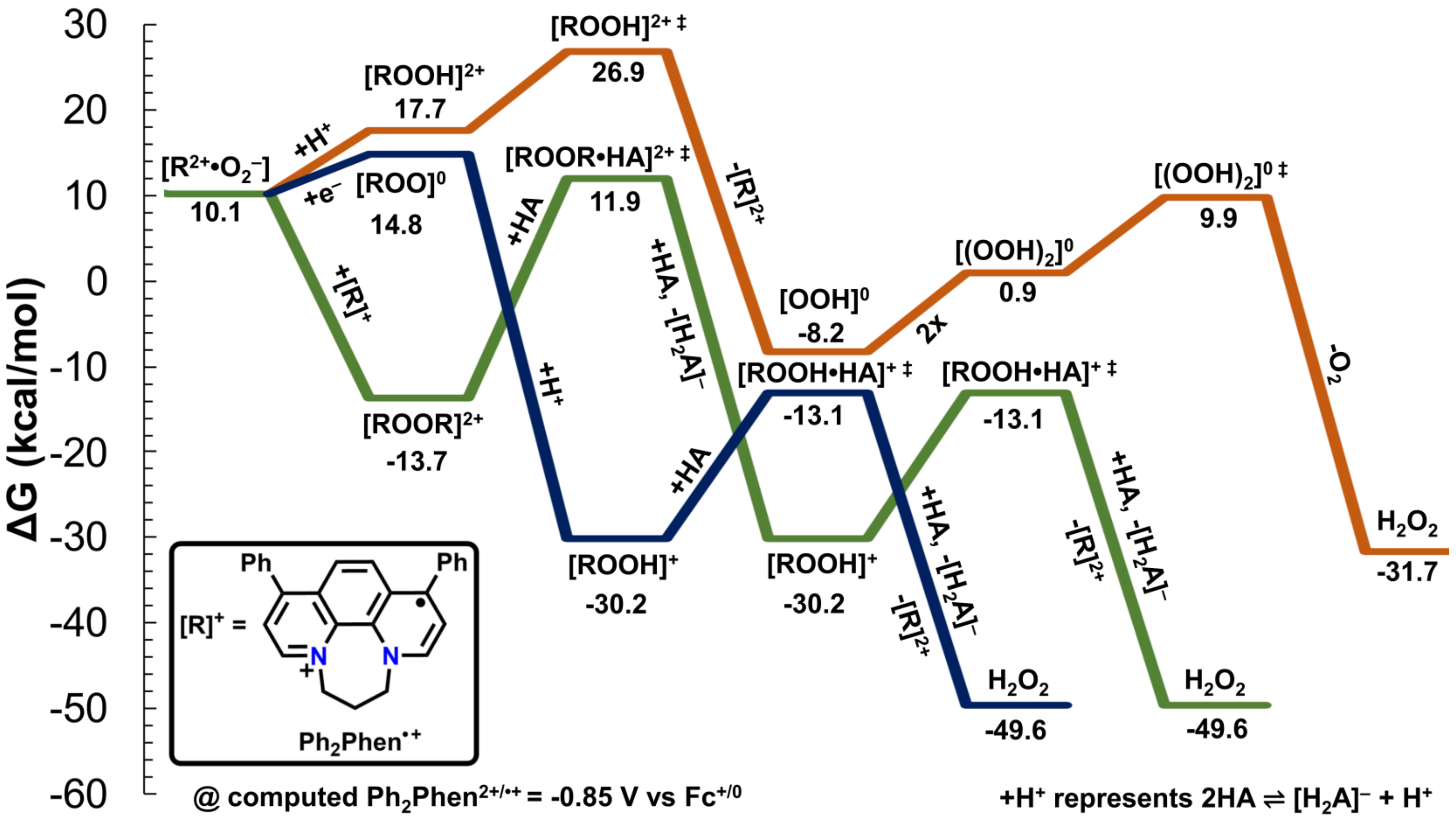

Figure S81. Comparison of computational pathways to H_2O_2 from the radical-ion pair considering TFAH as the acid.

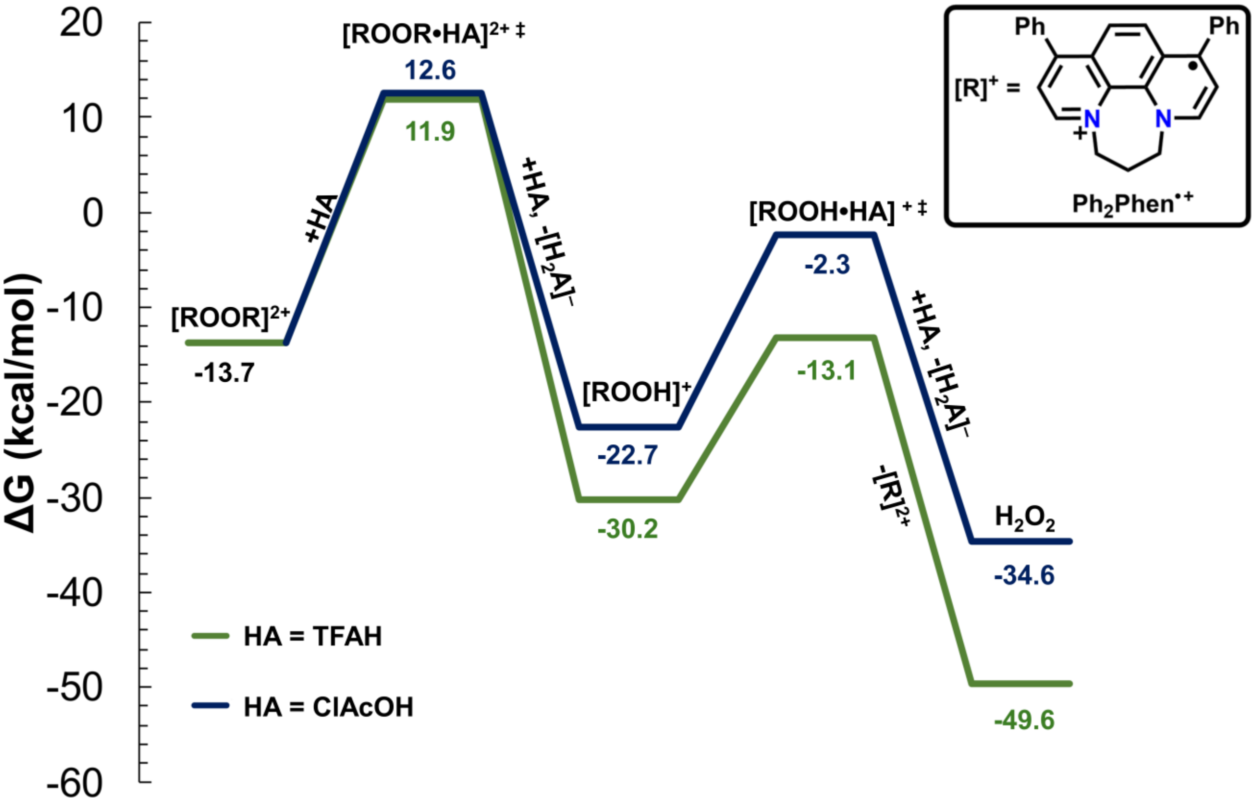

Figure S82. Comparison of computational pathways for acid-mediated dimer cleavage and H_2O_2 production for the strongest (TFAH) and weakest (ClAcOH) acids.

^{1}H-NMR Studies

^{1}H-NMR studies were conducted in order to observe reactivity of reduced **Ph_2Phen^{2+}** with O_2. To increase solubility of Ph_2Phen^{2+}, the Br^- counterions were exchanged for PF_6^- (**$Ph_2Phen(PF_6)_2$**) as described above. $Cp^*{}_2Fe$ was used as a chemical reductant in solution and added in a stoichiometric amount. NMR samples were made in a N_2-filled glovebox. Samples were exposed to air for ~1 h prior to spectra being taken.

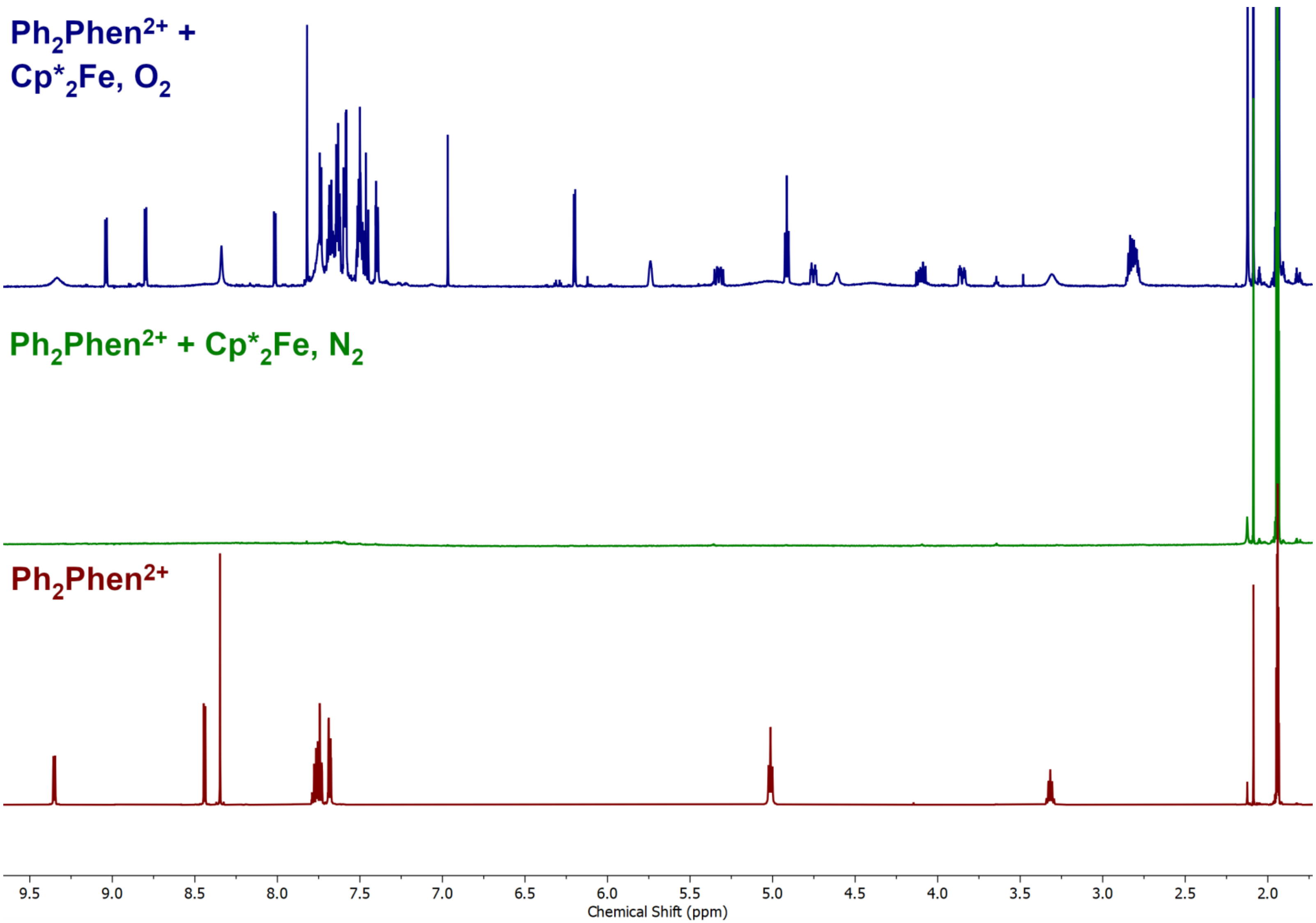

Figure S83. ^{1}H NMR spectra of **$Ph_2Phen(PF_6)_2$** (red) in the presence of $Cp^*{}_2Fe$ under N_2 (green) and exposed to air (blue). Conditions: 3.2 mM [**$Ph_2Phen(PF_6)_2$**], 4 mM [$Cp^*{}_2Fe$]; MeCN-d_3; Varian 600 MHz.

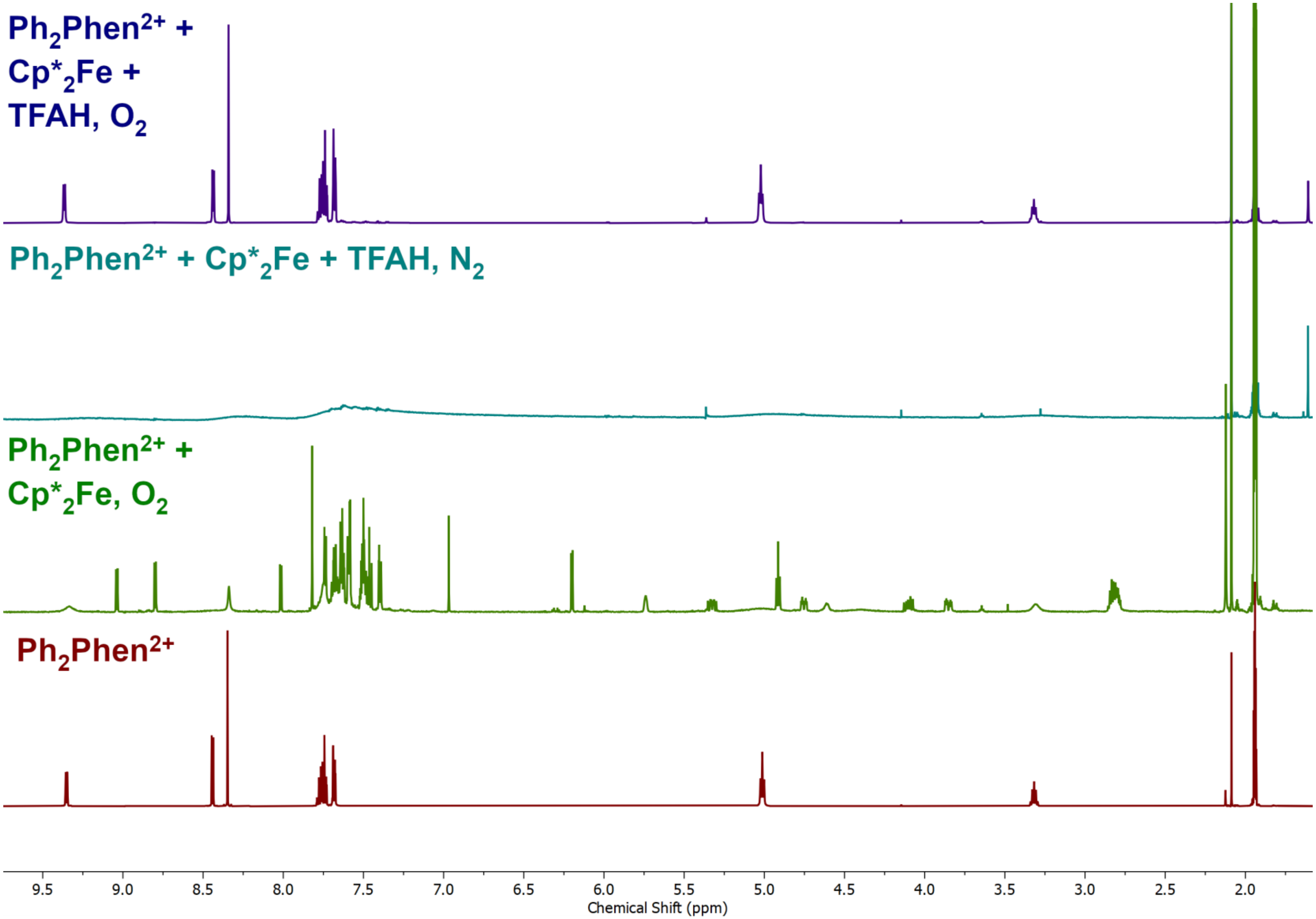

Figure S84. ^{1}H NMR spectra of **$Ph_2Phen(PF_6)_2$** (red) in the presence of $Cp^*{}_2Fe$ exposed to air (green) and TFAH under N_2 (teal) and exposed to air (blue). Conditions: 3.2 mM [**$Ph_2Phen(PF_6)_2$**], 4 mM [$Cp^*{}_2Fe$], 4 mM [TFAH]; MeCN-d_3; Varian 600 MHz.

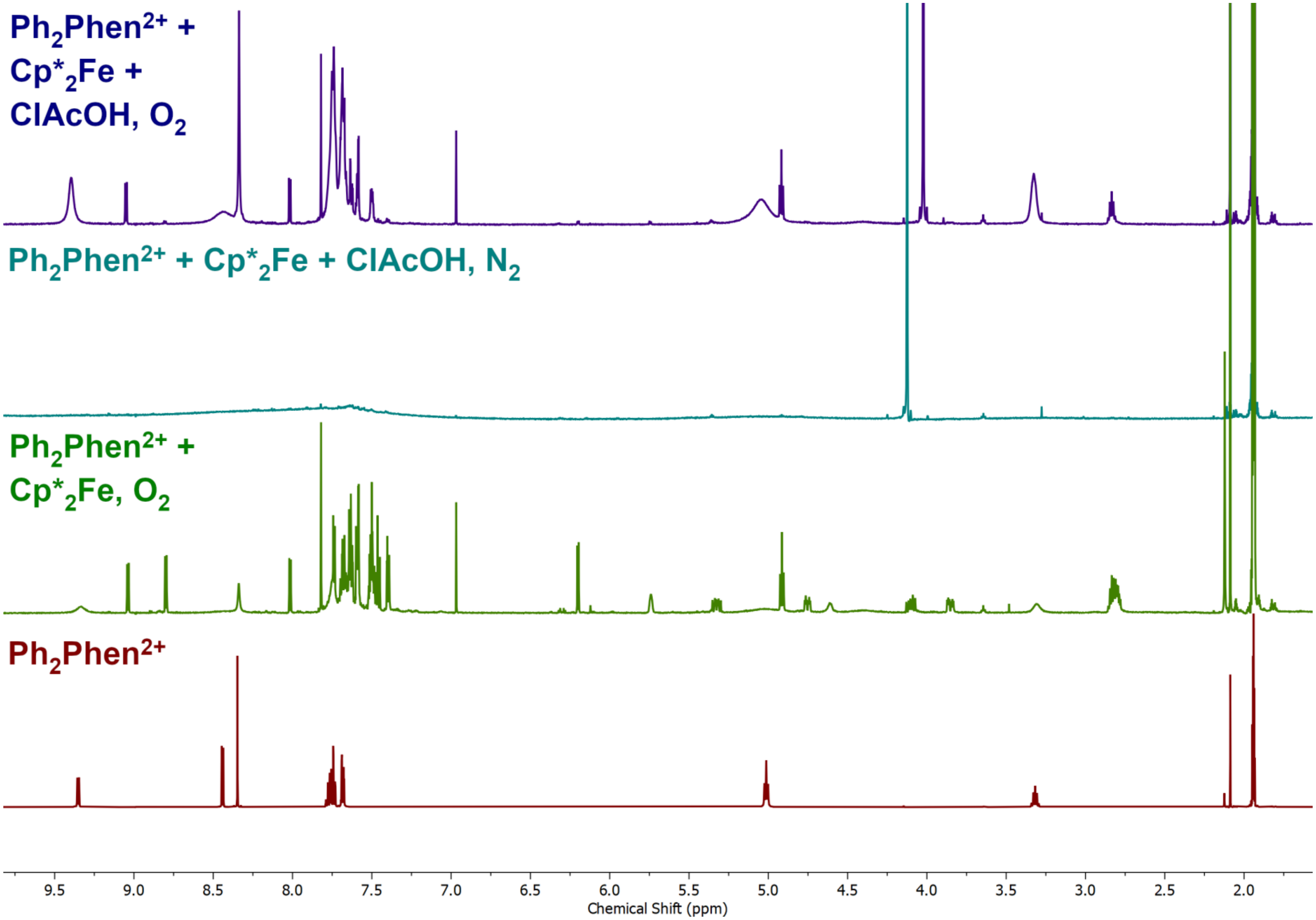

Figure S85. ^{1}H NMR spectra of **$Ph_2Phen(PF_6)_2$** in the presence of Cp^*_2Fe and ClAcOH under N_2 (color) and exposed to air (color). Conditions: 3.2 mM [**$Ph_2Phen(PF_6)_2$**], 4 mM [Cp^*_2Fe], 4 mM [ClAcOH]; MeCN-*d*$_3$; Varian 600 MHz.

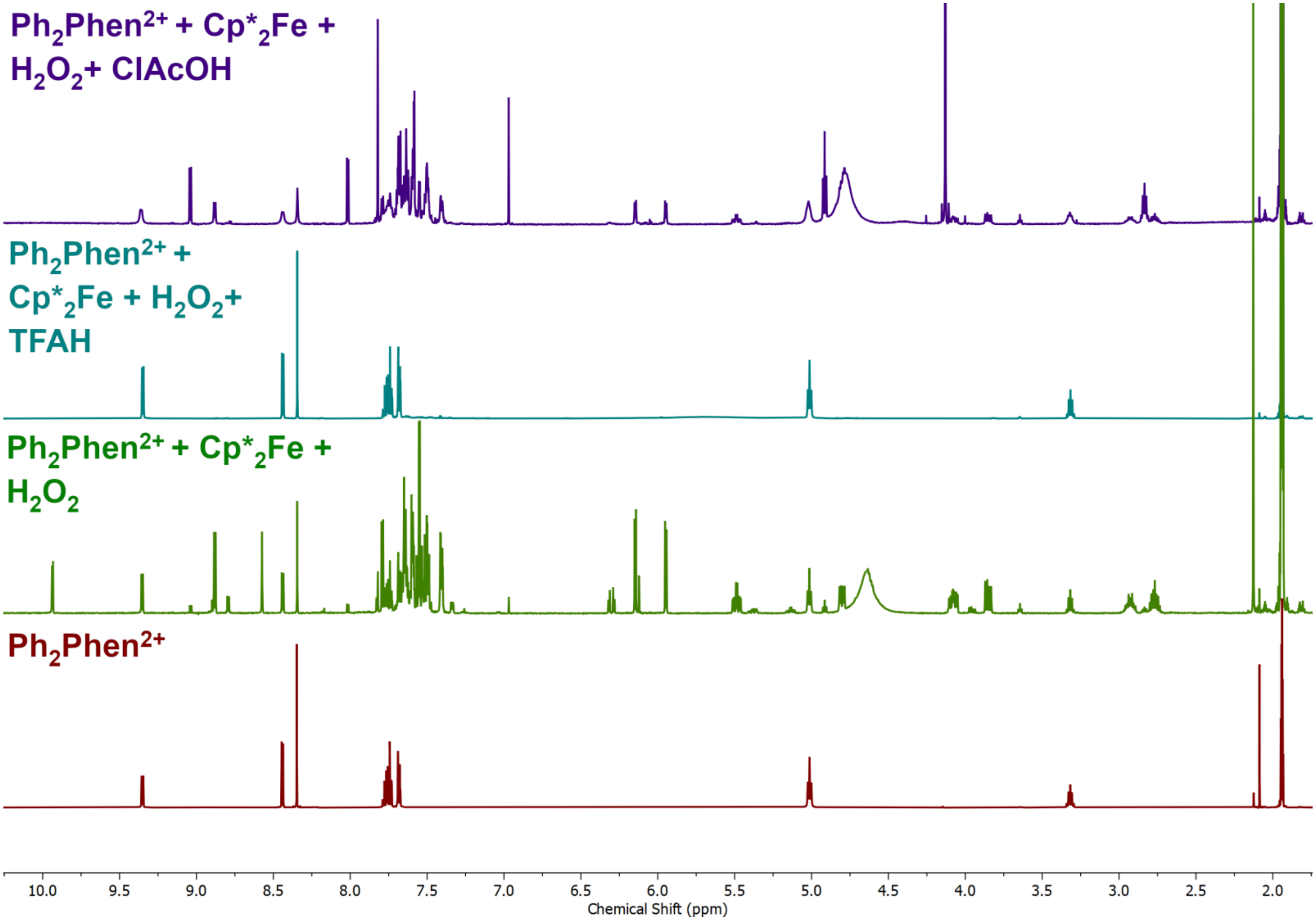

Figure S86. ^{1}H-NMR spectra of **Ph$_2$Phen(PF$_6$)$_2$** (red) in the presence of Cp*$_2$Fe and urea•H$_2$O$_2$ (green) with TFAH (teal) and ClAcOH (purple) under N$_2$ atmosphere. Conditions: 3.7 mM [**Ph$_2$Phen(PF$_6$)$_2$**], 4 mM [Cp*$_2$Fe] if present, 5.3 mM [urea•H$_2$O$_2$] if present, 4 mM [TFAH] if present, 5.3 mM [ClAcOH] if present; N$_2$ atmosphere; MeCN-d_3; Varian 600 MHz.

Table S7. Summary of Best Performing Previously Reported Metal-Free ORR Systems.

Catalyst	Acid (solvent)	E_{cat} (V)	*η* (Product)	Rate Constant	Measurement	Ref.
Ph_2Phen^{2+}	TFAH (MeCN)	–0.72 vs. Fc	0.78 (H_2O_2)	k_{obs} = 1.73 x 10^4 s^{-1}	spectrochemical	This Work
Ph_2Phen^{2+}	Cl_3AcOH (MeCN)	–0.72 vs. Fc	0.47 (H_2O_2)	k_{obs} =1.15 x 10^4 s^{-1}	spectrochemical	This Work
Ph_2Phen^{2+}	Cl_2AcOH (MeCN)	–0.72 vs. Fc	0.39 (H_2O_2)	k_{obs} = 1.06 x 10^2 s^{-1}	spectrochemical	This Work
Ph_2Phen^{2+}	ClAcOH (MeCN)	–0.72 vs. Fc	0.22 (H_2O_2)	k_{obs} = 0.431 x 10^2 s^{-1}	spectrochemical	This Work
Im^+	TFAH (MeCN)	–0.67 vs. Fc	1.09 (H_2O)[a]	k_{obs} = 6.66 x 10^3 s^{-1}	spectrochemical	[34]
MV^{2+}	ClAcOH (DMSO)	–0.45 vs. SCE	--	k_{cat} = 2.3 x 10^5 $M^{-1}s^{-1}$	electrochemical	[35]
$Ac(H)^+$	ClAcOH (DMSO)	–0.503 vs. SCE	--	k_{cat} = 2.0 x 10^{-6} $M^{-1}s^{-1}$	electrochemical	[36]
$[4]^{2+}$	$MeSO_3H$ (MeCN)	–0.53 vs. Fc	--	k_{cat} = 367 $M^{-1}s^{-1}$	spectrochemical	[37]
ImBenz-H	pH 13 Phosphate Buffer	0.39 vs. RHE	--	k_{obs} = 3.3 x 10^3 s^{-1}	electrochemical	[38]

[a]calculated using Eq. S6

X-Ray Crystallographic Details

An orange, block shaped crystal of **Ph_2Phen^{2+}** measuring 0.035×0.045×0.079 mm was coated with Paratone oil and mounted on a MiTeGen micromount. Data for **Ph_2Phen^{2+}** were measured on a Bruker D8 VENTURE dual wavelength Mo/Cu Kappa four-circle diffractometer equipped with a PHOTON III detector and an Incoatec IμS 3.0 microfocus sealed X-ray tube (Mo K_α, λ=0.71073 Å) using a HELIOS double bounce multilayer mirror as monochromator. The crystal temperature was controlled with an Oxford Cryostream 800 low temperature device. Data collection and processing were done within the Bruker APEX5 software suite.[39] All data were integrated with the Bruker SAINT 8.40B software using a narrow-frame algorithm. Data were corrected for absorption effects using a Multi-Scan method (SADABS).The structure was solved by direct methods with SHELXT[40] and refined by full-matrix least-squares methods against F^2 using SHELXL[41] within OLEX2.[40] All non-hydrogen atoms were refined with anisotropically. Hydrogen atoms were placed in geometrically calculated positions with U_{iso} = 1.2U_{equiv} of the parent atom (1.5U_{equiv} for methyl). This report and the CIF file were generated using FinalCif.[42]

Refinement details for Ph_2Phen^{2+}

The relative occupancy of the disordered atoms was freely refined. No constraints or restraints were used.

Table S8. Crystal data and structure refinement for **Ph_2Phen^{2+}**

CCDC number	2346924
Empirical formula	$C_{27}H_{22}Br_2N_2$
Formula weight	534.28
Temperature [K]	100.00
Wavelength [Å]	0.71073
Crystal size [mm^3]	0.035×0.045×0.079
Crystal habit	orange block
Crystal system	triclinic
Space group	$P\bar{1}$ (2)
a [Å]	8.1468(4)
b [Å]	11.9512(7)
c [Å]	13.4202(8)
α [°]	64.647(2)

β [°]	75.441(2)
γ [°]	73.068(2)
Volume [Å^3]	1117.42(11)
Z	2
ρ_{calc} [gcm^{-3}]	1.588
μ [mm^{-1}]	3.645
F(000)	536
2θ range [°]	4.05 to 54.95 (0.77 Å)
Index ranges	$-10 \le h \le 9$<br>$-15 \le k \le 15$<br>$-17 \le l \le 17$
Reflections collected	32293
Independent reflections	5129<br>[R_{int} = 0.0777]
Data / Restraints / Parameters	5129 / 0 / 293
Goodness-of-fit on F^2	1.032
Final R indexes [$I \geq 2\sigma(I)$]	R_1 = 0.0357<br>wR_2 = 0.0777
Final R indexes [all data]	R_1 = 0.0536<br>wR_2 = 0.0854
Largest peak/hole [eÅ^{-3}]	0.83/−0.49

References

(1) Li, Q.; Batchelor-McAuley, C.; Lawrence, N. S.; Hartshorne, R. S.; Compton, R. G. Anomalous solubility of oxygen in acetonitrile/water mixture containing tetra-n-butylammonium perchlorate supporting electrolyte; the solubility and diffusion coefficient of oxygen in anhydrous acetonitrile and aqueous mixtures. *J. Electroanal. Chem.* **2013**, *688*, 328-335. DOI: 10.1016/j.jelechem.2012.07.039.

(2) Kütt, A.; Tshepelevitsh, S.; Saame, J.; Lõkov, M.; Kaljurand, I.; Selberg, S.; Leito, I. Strengths of Acids in Acetonitrile. *Eur. J. Org. Chem.* **2021**, *2021* (9), 1407-1419. DOI: 10.1002/ejoc.202001649.

(3) Harraz, D. M.; Weng, S.; Surendranath, Y. Electrochemically Quantifying Oxygen Reduction Selectivity in Nonaqueous Electrolytes. *ACS Catal.* **2023**, *13* (2), 1462-1469. DOI: 10.1021/acscatal.2c04564.

(4) Hooe, S. L.; Rheingold, A. L.; Machan, C. W. Electrocatalytic Reduction of Dioxygen to Hydrogen Peroxide by a Molecular Manganese Complex with a Bipyridine-Containing Schiff Base Ligand. *J. Am. Chem. Soc.* **2018**, *140* (9), 3232-3241. DOI: 10.1021/jacs.7b09027.

(5) Cook, E. N.; Dickie, D. A.; Machan, C. W. Catalytic Reduction of Dioxygen to Water by a Bioinspired Non-Heme Iron Complex via a 2+2 Mechanism. *J. Am. Chem. Soc.* **2021**, *143* (40), 16411-16418. DOI: 10.1021/jacs.1c04572.

(6) Cook, E. N.; Hooe, S. L.; Dickie, D. A.; Machan, C. W. Homogeneous Catalytic Reduction of O2 to H2O by a Terpyridine-Based FeN3O Complex. *Inorg. Chem.* **2022**, *61* (22), 8387-8392. DOI: 10.1021/acs.inorgchem.2c00524.

(7) Zhou, R.; Zheng, Y.; Jaroniec, M.; Qiao, S. Z. Determination of the Electron Transfer Number for the Oxygen Reduction Reaction: From Theory to Experiment. *ACS Catal.* **2016**, *6* (7), 4720-4728. DOI: 10.1021/acscatal.6b01581.

(8) Sathrum, A. J.; Kubiak, C. P. Kinetics and Limiting Current Densities of Homogeneous and Heterogeneous Electrocatalysts. *J. Phys. Chem. Lett.* **2011**, *2* (18), 2372-2379. DOI: 10.1021/jz2008227.

(9) McKinnon, M.; Rochford, J. Chapter 3.20 - Principles of Electrocatalysis. In *Green Chemistry*, Török, B., Dransfield, T. Eds.; Elsevier, 2018; pp 695-727.
(10) Wang, Y. H.; Pegis, M. L.; Mayer, J. M.; Stahl, S. S. Molecular Cobalt Catalysts for O2 Reduction: Low-Overpotential Production of H2O2 and Comparison with Iron-Based Catalysts. *J. Am. Chem. Soc.* **2017**, *139* (46), 16458-16461. DOI: 10.1021/jacs.7b09089.
(11) Hooe, S. L.; Machan, C. W. Dioxygen Reduction to Hydrogen Peroxide by a Molecular Mn Complex: Mechanistic Divergence between Homogeneous and Heterogeneous Reductants. *J. Am. Chem. Soc.* **2019**, *141* (10), 4379-4387. DOI: 10.1021/jacs.8b13373.
(12) Cook, E. N.; Courter, I. M.; Dickie, D. A.; Machan, C. W. Controlling product selectivity during dioxygen reduction with Mn complexes using pendent proton donor relays and added base. *Chem. Sci.* **2024**, 10.1039/D3SC02611F. DOI: 10.1039/D3SC02611F.
(13) *Gaussian 16, Revision B.01*; Frisch, M.J., Trucks, G.W., Schlegel, H.B., Scuseria, G.E., Robb, M.A., Cheeseman, J.R.; Scalmani, G.; Barone, V.; Petersson, G.A.; Nakatsuji, H.; Li, X.; Caricato, M.; Marenich, A.V.; Bloino, J., Janesko, B.G., Gomperts, R., Mennucci, B., Hratchian, H.P., Ortiz, J.V., Izmaylov, A.F., Sonnenberg, J.L., Williams-Young, D., Ding, F., Lipparini, F., Egidi, F., Goings, J., Peng, B., Petrone, A., Henderson, T., Ranasinghe, D., Zakrzewski, V.G., Gao, J., Rega, N., Zheng, G., Liang, W., Hada, M., Ehara, M., Toyota, K., Fukuda, R., Hasegawa, J., Ishida, M., Nakajima, T., Honda, Y., Kitao, O., Nakai, H., Vreven, T., Throssell, K., Montgomery Jr., J.A., Peralta, J.E., Ogliaro, F., Bearpark, M.J., Heyd, J.J., Brothers, E.N., Kudin, K.N., Staroverov, V.N., Keith, T.A., Kobayashi, R., Normand, J., Raghavachari, K., Rendell, A.P., Burant, J.C., Iyengar, S.S., Tomasi, J., Cossi, M., Millam, J.M., Klene, M., Adamo, C., Cammi, R., Ochterski, J.W., Martin, R.L., Morokuma, K., Farkas, O., Foresman, J.B., Fox, D.J. Gaussian, Inc., Wallingford CT (2016) GaussView 5.0. Wallingford, E.U.A.
(14) Becke, A. D. Density-functional thermochemistry. III. The role of exact exchange. *J. Chem. Phys.* **1993**, *98* (7), 5648-5652. DOI: 10.1063/1.464913.
(15) Lee, C.; Yang, W.; Parr, R. G. Development of the Colle-Salvetti correlation-energy formula into a functional of the electron density. *Phys. Rev. B* **1988**, *37* (2), 785-789.
(16) Vosko, S. H.; Wilk, L.; Nusair, M. Accurate spin-dependent electron liquid correlation energies for local spin density calculations: a critical analysis. *Can. J. Phys.* **1980**, *58* (8), 1200-1211. DOI: 10.1139/p80-159.
(17) Stephen, P. J.; Devlin, F. J.; Chabalowski, C. F.; Frisch, M. J. Ab Initio Calculation of Vibrational Absorption and Circular Dichroism Spectra Using Density Functional Force Fields. *J. Phys. Chem.* **1994**, *98* (45), 11623-11627.
(18) Weigend, F.; Ahlrichs, R. Balanced basis sets of split valence, triple zeta valence and quadruple zeta valence quality for H to Rn: Design and assessment of accuracy. *Phys. Chem. Chem. Phys.* **2005**, *7* (18), 3297-3305. DOI: 10.1039/b508541a.
(19) Weigend, F. Accurate Coulomb-fitting basis sets for H to Rn. *Phys. Chem. Chem. Phys.* **2006**, *8* (9), 1057-1065. DOI: 10.1039/b515623h.
(20) Grimme, S.; Antony, J.; Ehrlich, S.; Krieg, H. A consistent and accurate ab initio parametrization of density functional dispersion correction (DFT-D) for the 94 elements H-Pu. *J. Chem. Phys.* **2010**, *132* (15), 154104-154104. DOI: 10.1063/1.3382344.
(21) Grimme, S.; Ehrlich, S.; Goerigk, L. Effect of the damping function in dispersion corrected density functional theory. *J. Comput. Chem.* **2011**, *32* (7), 1456-1465. DOI: 10.1002/jcc.21759.
(22) Marenich, A. V.; Cramer, C. J.; Truhlar, D. G. Universal solvation model based on solute electron density and on a continuum model of the solvent defined by the bulk dielectric constant and atomic surface tensions. *J. Phys. Chem. B* **2009**, *113* (18), 6378-6396. DOI: 10.1021/jp810292n.

(23) Ribeiro, R. F.; Marenich, A. V.; Cramer, C. J.; Truhlar, D. G. Use of solution-phase vibrational frequencies in continuum models for the free energy of solvation. *J. Phys. Chem. B* **2011**, *115* (49), 14556-14562. DOI: 10.1021/jp205508z.
(24) *Goodvibes v.3.0.1*; 2019. Luchini G., Alegre-Requena J.V., Funes-Ardoiz I. and Paton R.S. GoodVibes: automated thermochemistry for heterogeneous computational chemistry data. *F1000Research* 2020, 9 (Chem Inf Sci):291.
(25) Neese, F. Software update: The ORCA program system—Version 5.0. *WIREs Computational Molecular Science* **2022**, *12* (5), e1606. DOI: 10.1002/wcms.1606.
(26) Dunning, T. H., Jr. Gaussian basis sets for use in correlated molecular calculations. I. The atoms boron through neon and hydrogen. *J. Chem. Phys.* **1989**, *90* (2), 1007-1023. DOI: 10.1063/1.456153.
(27) Weigend, F.; Köhn, A.; Hättig, C. Efficient use of the correlation consistent basis sets in resolution of the identity MP2 calculations. *J. Chem. Phys.* **2002**, *116* (8), 3175-3183. DOI: 10.1063/1.1445115.
(28) Mardirossian, N.; Head-Gordon, M. ωB97M-V: A combinatorially optimized, range-separated hybrid, meta-GGA density functional with VV10 nonlocal correlation. *J. Chem. Phys.* **2016**, *144* (21), 214110. DOI: 10.1063/1.4952647.
(29) Hellweg, A.; Hättig, C.; Höfener, S.; Klopper, W. Optimized accurate auxiliary basis sets for RI-MP2 and RI-CC2 calculations for the atoms Rb to Rn. *Theor. Chem. Acc.* **2007**, *117* (4), 587-597. DOI: 10.1007/s00214-007-0250-5.
(30) Caldeweyher, E.; Bannwarth, C.; Grimme, S. Extension of the D3 dispersion coefficient model. *J. Chem. Phys.* **2017**, *147* (3), 034112. DOI: 10.1063/1.4993215.
(31) Caldeweyher, E.; Ehlert, S.; Hansen, A.; Neugebauer, H.; Spicher, S.; Bannwarth, C.; Grimme, S. A generally applicable atomic-charge dependent London dispersion correction. *J. Chem. Phys.* **2019**, *150* (15), 154122. DOI: 10.1063/1.5090222.
(32) Najibi, A.; Goerigk, L. DFT-D4 counterparts of leading meta-generalized-gradient approximation and hybrid density functionals for energetics and geometries. *J. Comput. Chem.* **2020**, *41* (30), 2562-2572. DOI: 10.1002/jcc.26411.
(33) Moreno, J. J.; Hooe, S. L.; Machan, C. W. DFT Study on the Electrocatalytic Reduction of CO2 to CO by a Molecular Chromium Complex. *Inorg. Chem.* **2021**, *60* (6), 3635-3650. DOI: 10.1021/acs.inorgchem.0c03136.
(34) Cook, E. N.; Davis, A. E.; Hilinski, M. K.; Machan, C. W. Metal-Free Homogeneous O2 Reduction by an Iminium-Based Electrocatalyst. *J. Am. Chem. Soc.* **2024**. DOI: 10.1021/jacs.3c14549.
(35) Andrieux, C. P.; Hapiot, P.; Savéant, J. M. Electron transfer coupling of diffusional pathways. Homogeneous redox catalysis of dioxygen reduction by the methylviologen cation radical in acidic dimethylsulfoxide. *J. Electroanal. Chem. Interf. Electrochem.* **1985**, *189* (1), 121-133. DOI: 10.1016/0368-1874(85)85630-6.
(36) Audebert, P.; Hapiot, P. Preparation and electrochemistry of several substituted 9-(4-R-phenyl)-N-methylacridinium salts.: Kinetic analysis of the O2 catalytic reduction in acidic dimethylsulfoxide and in hydrophobic Nafion® gels. *J. Electroanal. Chem.* **1993**, *361* (1), 177-183. DOI: 10.1016/0022-0728(93)87052-W.
(37) Karimi, M.; Borthakur, R.; Dorsey, C. L.; Chen, C.-H.; Lajeune, S.; Gabbaï, F. P. Bifunctional Carbenium Dications as Metal-Free Catalysts for the Reduction of Oxygen. *J. Am. Chem. Soc.* **2020**, *142* (32), 13651-13656. DOI: 10.1021/jacs.0c04841.
(38) Tanjedrew, N.; Thammanatpong, K.; Surawatanawong, P.; Chakthranont, P.; Chantarojsiri, T.; Unjarern, T.; Kiatisevi, S. Tunable Metal-free Imidazole-Benzimidazole Electrocatalysts for Oxygen Reduction in Aqueous Solutions. *Chem. Eur. J.* **2023**, *30* (5), e202302854. DOI: 10.1002/chem.202302854.
(39) *Saint; APEX5*; Bruker AXS Inc.: Madison, Wisconsin, USA, 2019.

(40) Sheldrick, G. M. SHELXT - Integrated space-group and crystal-structure determination. *Acta Cryst.* **2015**, *71* (1), 3-8. DOI: 10.1107/S2053273314026370.
(41) Sheldrick, G. Crystal structure refinement with SHELXL. *Acta Cryst. C* **2015**, *71* (1), 3-8. DOI: doi:10.1107/S2053229614024218.
(42) Kratzert, D. *FinalCif*. https://dkratzert.de/finalcif.html.
